# High-resolution genome-wide functional dissection of transcriptional regulatory regions and nucleotides in human

**DOI:** 10.1038/s41467-018-07746-1

**Published:** 2018-12-19

**Authors:** Xinchen Wang, Liang He, Sarah M. Goggin, Alham Saadat, Li Wang, Nasa Sinnott-Armstrong, Melina Claussnitzer, Manolis Kellis

**Affiliations:** 1 Department of Biology, Massachusetts Institute of Technology, Cambridge, MA 02139 USA; 2 Broad Institute of MIT and Harvard, Cambridge, MA 02142 USA; 3 Computer Science and Artificial Intelligence Laboratory, Massachusetts Institute of Technology, Cambridge, MA 02139 USA; 4 Division of Gerontology, Department of Medicine, Beth Israel Deaconess Medical Center, Boston, MA 02215 USA; 5 Institute of Nutritional Science, University of Hohenheim, Garbenstrasse 30, 70599 Stuttgart, Germany; 6 Harvard Medical School, Harvard University, Boston, MA 02215 USA; 7 Present Address: Institute for Genomic Medicine, Columbia University, New York, NY 10024 USA

## Abstract

Genome-wide epigenomic maps have revealed millions of putative enhancers and promoters, but experimental validation of their function and high-resolution dissection of their driver nucleotides remain limited. Here, we present HiDRA (High-resolution Dissection of Regulatory Activity), a combined experimental and computational method for high-resolution genome-wide testing and dissection of putative regulatory regions. We test ~7 million accessible DNA fragments in a single experiment, by coupling accessible chromatin extraction with self-transcribing episomal reporters (ATAC-STARR-seq). By design, fragments are highly overlapping in densely-sampled accessible regions, enabling us to pinpoint driver regulatory nucleotides by exploiting differences in activity between partially-overlapping fragments using a machine learning model (SHARPR-RE). In GM12878 lymphoblastoid cells, we find ~65,000 regions showing enhancer function, and pinpoint ~13,000 high-resolution driver elements. These are enriched for regulatory motifs, evolutionarily-conserved nucleotides, and disease-associated genetic variants from genome-wide association studies. Overall, HiDRA provides a high-throughput, high-resolution approach for dissecting regulatory regions and driver nucleotides.

## Abstract (web summary)

Millions of enhancers are predicted, but their validation remains challenging. Here, the authors report genome-wide enhancer function quantification and high-resolution dissection for millions of accessible DNA fragments, revealing driver nucleotides and helping interpret non-coding disease variants.

## Introduction

Precise spatiotemporal control of gene expression is achieved by the interplay between non-coding regulatory elements, including distal enhancers and proximal promoters, and the transcriptional regulators they help recruit or repel, thus modulating the expression of nearby genes^1–3^. Unlike protein-coding genes, which can be readily identified by their sequence properties and evolutionary signatures, gene-regulatory elements lack highly predictive sequence patterns and show only modest evolutionary conservation at the nucleotide level^1,4^. Thus, systematic recognition of gene-regulatory elements has relied on mapping of their epigenomic signatures, including DNA accessibility, histone modifications, and DNA methylation^5–7^. For example, both enhancers and promoters have high DNA accessibility and low H3K27me3, but distal enhancers show relatively higher H3K27ac and H3K4me1 while promoters show relatively higher H3K9ac and H3K4me3^8,9^. However, many regions showing such epigenomic marks do not experimentally drive reporter gene expression, and some regions driving gene expression lack endogenous signatures^10–12^. Moreover, epigenomic signatures are often low-resolution, with important driver regulatory nucleotides comprising only a small subset of the larger regions showing epigenomic signatures^13^.

Experimental dissection of enhancer and promoter regions has been traditionally expensive, laborious, low-throughput, and low-resolution, lacking the resolution to pinpoint individual regulatory driver nucleotides without recourse to extensive mutagenesis. Several recent high-throughput reporter assays for enhancer function enable testing of thousands of distinct DNA sequences simultaneously, by cloning variable DNA fragments into common reporter constructs, and using high-throughput sequencing to quantify fragment activity. Synthesis-based approaches (e.g., MPRA^14^, CRE-seq^15^) use oligonucleotide synthesis technology to generate elements and coupled barcodes. Genome-fragmentation approaches (e.g., STARR-seq^16^, Cap-STARR-seq^17,18^, ChIP-STARR^19^) use DNA fragments collected or captured from genomic DNA. For synthesis-based approaches, technical limitations of oligonucleotide synthesis technology restrict the tested DNA fragment lengths to 130–230 nucleotides (nt), and the number of tested constructs to 100,000–200,000 sequences per array. For genome-fragmentation approaches, random fragmentation of the entire genome results in only shallow coverage of regulatory elements, while synthesis-based capture is limited in the number of regions interrogated due to its reliance on oligonucleotide synthesis, and ChIP-based capture is limited in only one or few transcription factors at a time. To recognize driver nucleotides within tested regions, synthesis-based approaches have used systematic mutagenesis^20^ or tiling at regularly-spaced intervals^13^, but both require synthesis of many constructs for fine-mapping each region, thus reducing the number of regions that can be dissected at high resolution.

Here, we present High-resolution Dissection of Regulatory Activity (HiDRA), a method for high-resolution inference of transcriptional regulatory activity across all accessible regions of the genome. HiDRA overcomes some limitations of previous technologies and combine their advantages, enabling high-throughput and high-resolution inference of regulatory activity. Briefly, we first extract accessible DNA regions using ATAC-seq^21^, size-select for constructs 150–500 nt long, and incorporate them in self-reporting episomal constructs (ATAC-STARR-seq), by insertion in the 3′ untranslated region (3′ UTR) of reporter genes, thus enabling them to drive their own transcription and serve as their own barcodes, providing a quantitative readout of their activity. We then exploit the dense sampling of accessible regions and the partially-overlapping nature of tested fragments for high-resolution inferences using a machine learning approach (SHARPR-RE). Our approach overcomes the construct-length and region-count limitations of synthesis-based technologies at substantially lower cost, and our ATAC-based selection of open chromatin regions concentrates the signal on likely regulatory regions and enables high-resolution inferences. Altogether, in a single experiment we test millions of enhancer constructs of comparable length to low-throughput studies while achieving the high-resolution dissection of systematic perturbation studies. We apply HiDRA to infer genome-wide regulatory activity across ~7 million DNA fragments preferentially selected from accessible chromatin in the GM12878 lymphoblastoid cell line, resulting in ~65,000 discrete genomic regions showing significant regulatory function. These are enriched for endogenous active histone marks (including H3K9ac, H3K27ac), regulatory sequence motifs, and regions bound by immune regulators. Our selection approach resulted in highly-overlapping fragments (~32,000 regions covered by 10+ unique fragments, ~12,500 by 20+ fragments), enabling us to pinpoint “driver” regulatory nucleotides that are critical for transcriptional enhancer activity. We discover ~13,000 of these high-resolution driver elements, which are enriched for regulatory motifs and evolutionarily conserved nucleotides, and help predict causal genetic variants underlying disease from genome-wide association studies. Overall, HiDRA provides a general, scalable, and high-throughput approach for the high-resolution experimental dissection of regulatory regions and driver nucleotides in the context of human biology and disease.

## Results

### HiDRA experimental method overview

**Fig. 1:**
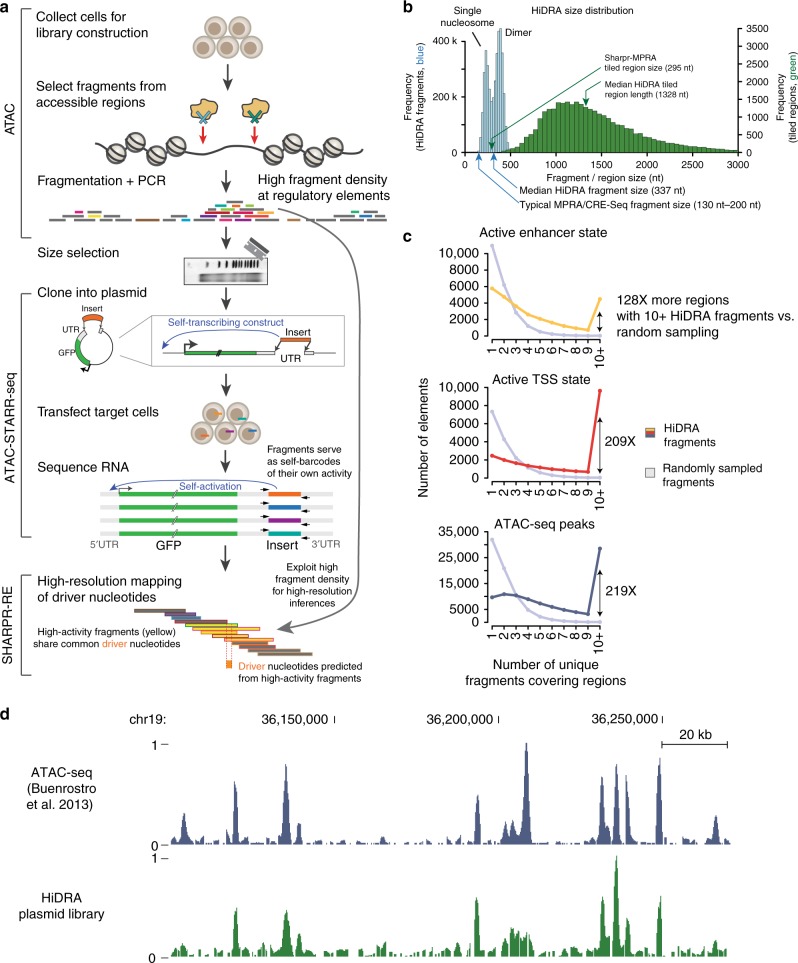
Overview of HiDRA. **a** Cells with the desired genotype and open chormatin patterns are selected for library construction. Tn5 transposase is used to preferentially fragment genomic DNA at regions of open chromatin. Fragments are then size-selected on an agarose gel and mtDNA contamination is removed by selective CRISPR-Cas9 degradation. The fragment library is amplified by PCR and cloned into an enhancer reporter vector. Gel image adapted from Buenrostro et al.^21^. Fragments are cloned into the STARR-seq vector backbone, introduced into target cells (which can differ from cells used to construct the library), and RNA is collected and sequenced. After data processing, the activity of partially-overlapping fragments is compared to identify driver nucleotides using the SHARPR-RE algorithm. **b** Size distribution of HiDRA library fragments (blue) and tiled regions (green). Bimodal shape for library fragment sizes is due to Tn5 preference to cut adjacent to nucleosomes. Fragment bin size = 20 nt, region bin size = 50 nt. **c** Number of ChromHMM-predicted active enhancer, active TSS and ATAC-seq peaks covered by multiple unique HiDRA fragments. **d** HiDRA plasmid library recapitulates the genomic coverage of a conventional ATAC-seq experiment

HiDRA leverages the selective fragmentation of genomic DNA at regions of open chromatin to generate fragment libraries that densely cover putative transcriptional regulatory elements. The experimental component of HiDRA is the combination of ATAC-seq and STARR-seq (i.e., ATAC-STARR-seq): fragments are enriched from open chromatin and regulatory regions using ATAC-seq (Assay for Transposase­Accessible Chromatin with high-throughput sequencing) and subsequently cloned into the 3′ UTR of a reporter gene on the self­-transcribing enhancer reporter vector used in Self-Transcribing Active Regulatory Region sequencing (STARR-seq)^13,16,21^. Fragments with transcriptional regulatory activity promote self­-transcription such that active segments of DNA can be identified and quantified by high–throughput RNA sequencing to produce a quantitative readout of enhancer activity (Fig. 1a). Library construction can be completed in 2–3 days and requires as few as 10^4^­–10^5^ cells as input starting material.

We constructed a HiDRA library with 9.7 million total unique mapping fragments, of which 4 million had a frequency greater than 0.1 reads per million (RPM; non-mitochondrial reads). More than 99% of fragments had lengths between 169 nt and 477 nt (median: 337 nt), with the fragment length distribution showing two peaks spaced by ~147 nt, corresponding to the length of DNA wrapped around each nucleosome (blue bars, Fig. 1b). In contrast to unbiased fragmentation of the genome, our library has much higher efficiency for selectively targeting accessible DNA regions that are more likely to play gene-regulatory roles. Our HiDRA library covers 4486 predicted enhancers and 9631 predicted promoters (“Active Transcription Start Site (TSS)” state^5,6,14^) with more than 10 unique fragments (Fig. 1c, colored lines), a ~130-fold and ~210-fold enrichment compared with 35 enhancer and 46 promoter regions expected to be covered by chance at the same coverage. This indicates that HiDRA library construction successfully targets predicted regulatory regions rather than randomly fragmenting the genome. Even among enhancer and promoter regions and ATAC-seq peaks, those with higher expected activity are preferentially selected by HiDRA, as they show higher accessibility and are thus more likely to be cloned in our library and tested by our episomal reporters (Supplementary Fig. 1).

Our cloning strategy is specifically designed to densely sample regulatory regions, in order to enable high-resolution inference of regulatory activity from highly overlapping fragments. Indeed, we found up to 370 unique fragments per region in our HiDRA libraries, with ~32,000 genomic intervals containing at least 10 overlapping fragments (“tiled regions”, green bars, Fig. 1b) and ~2750 containing at least 50 fragments, compared with 180 and 0 that would be expected by randomly selected fragments, respectively. In addition to clustering of tested fragments within the same region, high-resolution inference relies on partially-overlapping rather than fully-overlapping fragments, which requires a random fragmentation pattern. Indeed, the Tn5 transposase we used here inserts randomly into the genome, and indeed the resulting DNA fragments provide a dense sampling of start and end positions that mirrors the peaks of ATAC-seq experiments (Fig. 1d), indicating that accessible regions most likely to show regulatory activity will have both higher representation in our libraries, and also more starting and ending positions that can help identify driver nucleotides.

### Identification of DNA fragments with regulatory activity

**Fig. 2:**
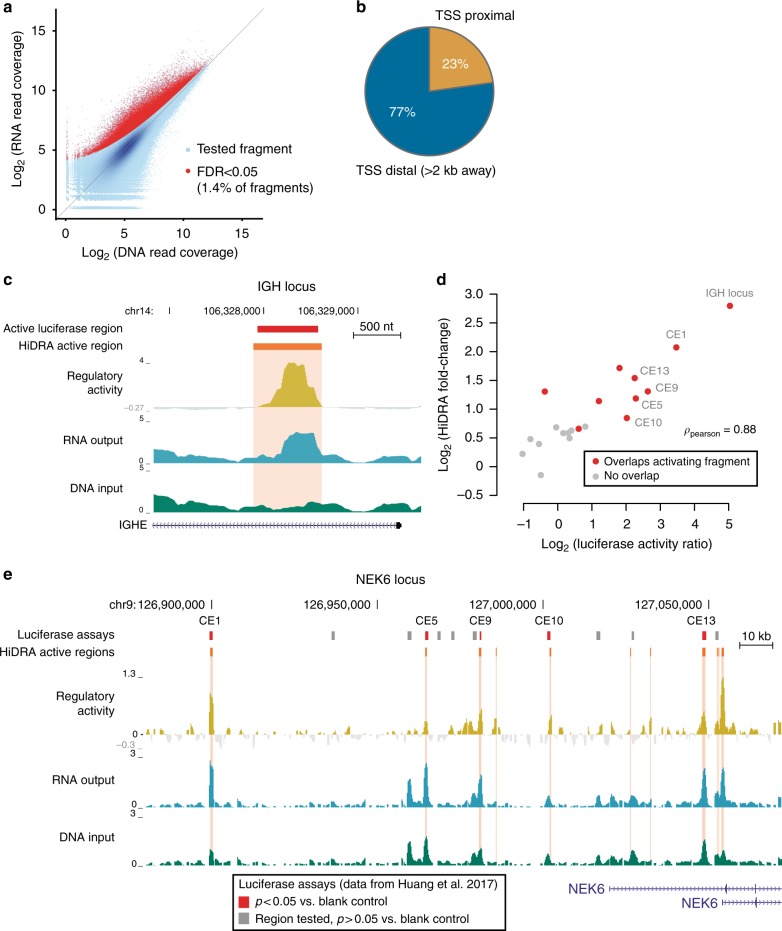
HiDRA identifies transcriptional regulatory elements. **a** Scatterplot of abundances for HiDRA fragments in input (plasmid DNA) and output (RNA) samples. Abundances calculated after merging all five replicates. Active HiDRA fragments called by DESeq2 highlighted with red dots (FDR < 0.05), blue color intensity corresponds to greater density of points. **b** The majority of HiDRA active regions are distal to annotated TSSs ( > 2 kb). **c** HiDRA identifies enhancer activity within an intron in the immunoglobulin heavy chain locus. Red bar, DNA segment active in luciferase assay performed by Huang et al.^23^. Orange bar and highlight, region identified by HiDRA as having transcriptional regulatory activity. **d** Quantitative comparison of luciferase assay activity levels to HiDRA for 21 predicted enhancer elements. HiDRA signal corresponds to maximum activity within the region tested by luciferase, and luciferase value corresponds to median normalized activity over biological replicates. Pearson correlation calculated after log2 transformation. **e** Comparison of HiDRA-called active regions with luciferase assay results for 13 enhancers at the NEK6 locus. Luciferase experiments are colored in red or gray depending on whether DNA fragments drive luciferase activity in GM12878 cells as determined by Huang et al.^23^

To evaluate the ability of each cloned DNA fragment to promote gene expression, we transfected our HiDRA library into GM12878 lymphoblastoid cells, collected RNA 24 h post-transfection, and measured the abundance of transcribed fragments by high-throughput RNA sequencing. We carried out five replicate transfection experiments from the same plasmid library, each into ~120 million cells, and we observed a high degree of correlation in the RNA counts between replicates (0.95 Pearson correlation on average for fragments ≥ 1 RPM; 0.76 for ≥ 0.1 RPM; Supplementary Fig. 2). To quantify the regulatory activity of tested elements, we compared the number of RNA reads obtained for a fragment (corresponding to the expression level of the reporter gene, as the constructs are self-transcribing), relative to representation of that fragment in the non-transfected input plasmid library (thus normalizing the differential abundance of each fragment in our library). We observed a substantial number of fragments that are more prevalent in RNA than DNA, indicating capability of many HiDRA fragments to drive reporter gene expression (Fig. 2a).

Given the intentionally high initial complexity of our HiDRA library, many fragments will be sequenced with a relatively low depth of coverage. We therefore grouped fragments with a 75% reciprocal overlap to boost the read coverage of genomic regions and increase statistical power. This yielded 7.1 million unique “fragment groups” generated from merging 9.7 million HiDRA fragments. In total, we identified 95,481 fragment groups that promote reporter gene expression at an FDR cutoff of 0.05, which we will refer to as “active HiDRA fragments” (Fig. 2a, red dots, see Methods). These 95,481 active HiDRA fragments are located within 66,254 unique genomic intervals that we subsequently refer to as “active HiDRA regions” (Supplementary Data 1). Active HiDRA fragments showed a wide range of input DNA levels in our plasmid library, indicating that regulatory function and DNA accessibility rely on complementary sequence signals, and that DNA accessibility alone is not sufficient to predict episomal regulatory activity. We also found that active HiDRA regions are predominantly distal to annotated transcription start sites (TSSs) (Fig. 2b), validating the utility of HiDRA for pinpointing distal regulatory regions that are particularly challenging to identify.

As proof-of-concept that HiDRA is capable of identifying true enhancer elements, we examined the well-studied immunoglobulin heavy-chain enhancer within the intron of the immunoglobulin heavy constant epsilon (IGHE) gene^22^. We observed that the peak of HiDRA activity is centered precisely within the region previously identified as driving enhancer activity in low-throughput luciferase assays (Fig. 2c). To assess the quantitative accuracy of HiDRA relative to luciferase assays, we compared active HiDRA regions and luciferase results across 21 putative enhancers predicted and tested independently by Huang et al.^23^. We found a 0.88 Pearson correlation between measured luciferase activity and HiDRA activity, confirming the accuracy and quantitative nature of our high-throughput approach (Fig. 2d). A visualization of 14 luciferase-tested enhancers in the serine/threonine kinase NEK6 locus shows a strong correspondence between luciferase assay results and HiDRA active regions (Fig. 2e).

### HiDRA elements are enriched in promoters and enhancers

**Fig. 3:**
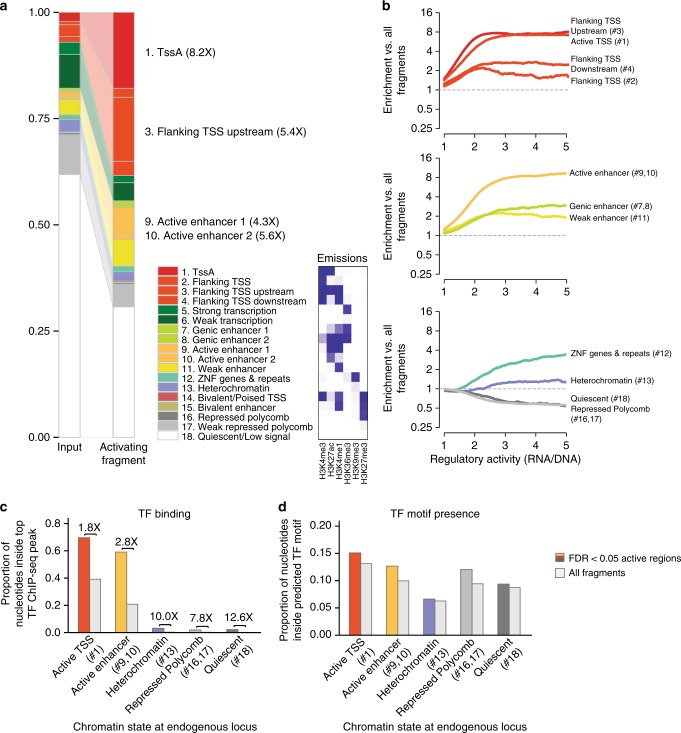
Active HiDRA fragments are enriched in endogenously active regulatory regions. **a** Overlap of active HiDRA fragments with different endogenous chromatin states. Heights correspond to proportion of nucleotides within active HiDRA fragments in each chromatin state. Inset: histone modification enrichments in each of 18 ChromHMM chromatin states **b** HiDRA fragment regulatory activity (fold-change increase in RNA levels) across different chromatin states. Numbers correspond to chromatin state numbers in 18-state ChromHMM model. **c**, **d** Endogenously inactive genomic regions have low levels of TF binding (**c**) but comparable TF motif composition (**d**) to predicted active regions. Colored bars, regions from each chromatin state overlapping active HiDRA regions. Gray bars, regions from each chromatin state overlapping all fragments tested

We surveyed the 95,481 active HiDRA fragments identified in GM12878 to assess shared common genomic or epigenomic characteristics. In comparison with the set of all HiDRA fragments tested, active fragments were eight times more likely to overlap regions annotated as active promoter chromatin states by ChromHMM based on the presence of H3K4me3 and H3K27ac, and five times more likely to overlap annotated Active Enhancer chromatin states, marked by H3K4me1 and H3K27ac (Fig. 3a). By contrast, weak enhancer chromatin states marked by H3K4me1 and absence of H3K27ac have substantially weaker enrichment (1.7-fold) within active HiDRA fragments than active enhancers, consistent with previous literature indicating that the presence of H3K27ac correlates with greater expression of nearby genes (Fig. 3b). Overall, 35% of all predicted active promoters (8355 regions) and 16% of all predicted active enhancers (5276 regions) overlapped at least one active HiDRA fragment.

In addition to active promoter and active enhancer chromatin states, the “TSS Flanking Upstream” chromatin state showed strong enrichment for active HiDRA fragments (7.3-fold higher than expected from the input library). This chromatin state is defined by the presence of both promoter and enhancer histone marks H3K4me1, H3K4me3, and H3K27ac, and was named “TSS Flanking” due its depletion at exactly the TSS position, but its enrichment 400 nt-1kb upstream of annotated transcription start sites^7^. However, 64% of its occurrences are > 2 kb from the nearest transcription start site, suggesting that a portion of genomic regions annotated as “TSS Flanking Upstream” may function biologically as distal enhancers (Supplementary Fig. 3).

When we computed enrichment of chromatin states as a function of HiDRA activity strength, we found a linear quantitative relationship for HiDRA activity levels up to ~2.5-fold RNA/DNA ratios, with increasing activity showing increasing chromatin state enrichment for both promoter and enhancer chromatin states (Fig. 3b). Surprisingly, this enrichment stayed constant thereafter for promoter regions, and increased modestly for enhancer regions, ultimately surpassing the enrichment seen for promoters. In fact, even though promoter chromatin states were more enriched at intermediate HiDRA activity levels, enhancer chromatin states were the most enriched at the highest HiDRA activity levels (*p* = 9.3 × 10^–102^, Mann–Whitney *U* test, Supplementary Fig. 4a), suggesting that enhancer elements have a greater dynamic range of regulatory activity potential, which has implications for the regulatory architecture of genes.

Surprisingly, fragments from heterochromatin-associated chromatin states showed a modest enrichment in active elements, indicating that DNA kept in an endogenous heterochromatic state may contain regulatory signals that become active once taken outside their repressive endogenous chromosomal context. The ZNF/repeats-associated chromatin state (marked by H3K36me3 and H3K9me3) showed a modest enrichment for lower HiDRA activity levels, but continued to increase linearly even at the highest activity levels, possibly due to active repetitive elements, as we discuss below. In contrast, quiescent and polycomb-repressed chromatin states showed a two-fold relative depletion for HiDRA active elements, accounting for the enrichments found in other states. The depletion of polycomb-repressed chromatin states may also reflect polycomb repression on the episomal plasmid.

We also studied the enrichment of HiDRA regions for individual histone marks profiled by the ENCODE project in GM12878^7^. Active promoter- and active-enhancer-associated acetylation marks H3K9ac and H3K27ac, histone turnover-associated H2A.Z, promoter- and enhancer-associated H3K4me3 and H3K4me1, and DNase I accessible chromatin were the most enriched individual marks within active HiDRA regions, while polycomb-repression-associated H3K27me3, heterochromatin-associated H3K9me3, and transcription-associated H3K36me3 were the least enriched compared with the input library (Supplementary Fig. 5).

As these elements are tested outside their endogenous chromatin context, we expect that they drive reporter gene transcription by recruiting transcriptional regulators in a sequence-specific way, and we sought to gain insights into the recruited factors. We calculated the overrepresentation of 651 transcription factor sequence motifs assembled by ENCODE in active HiDRA regions, and found enrichment for many distinct motifs for immune transcription factors (Supplementary Fig. 4b), including IRF, NFKB1, and RELA, corresponding to transcriptional regulators known to function in GM12878 compared with other human cell lines. The motifs enriched in promoter chromatin states were largely distinct from those enriched in enhancer chromatin states, highlighting the differential regulatory control of the two types of regions (Supplementary Figs. 4b–d). High-activity fragments showed distinct motif composition, and were enriched for GM12878 regulators including NF-kB (Supplementary Figs. 4e, f). These differences in motif content indicate that the two types of regions recruit different sets of transcriptional regulators both in their endogenous context and in our episomal assays, consistent with their distinct endogenous chromatin state and their distinct properties in our HiDRA assays.

### Regulatory activity outside promoters and enhancers

Even though HiDRA active regions were most enriched for enhancer and promoter states, they were not exclusive to them. In fact, consistent with recent studies^24,25^, approximately half of active HiDRA regions (52%) showed endogenous epigenomic signatures characteristic of repressed and inactive chromatin states, including quiescent, repressed polycomb, weak repressed polycomb, and heterochromatin.

As active chromatin states were defined based on the profiling of only a subset of known chromatin marks in GM12878, we reasoned that perhaps other marks may be marking these regions active, but that they were perhaps not profiled in GM12878 and thus missed by the reference genome annotations. For example, a recent study identified subclasses of active enhancer elements marked with H3K122ac or H3K64ac but not H3K27ac^12^. While these marks were not profiled in GM12878, inactive chromatin states that showed HiDRA activity were 8-fold to 13-fold more likely to be bound by transcription factors in ChIP-seq experiments in GM12878 than inactive chromatin states that lacked HiDRA activity (Fig. 3d), indicating that our assays can successfully recover active regions even outside active chromatin states, and highlighting the importance of our unbiased survey of open chromatin regions regardless of their endogenous chromatin marks.

As both high-throughput and low-throughput episomal assays test regions outside their endogenous chromatin context, we reasoned that some active HiDRA regions with inactive chromatin signatures may reflect endogenously inactive regions that become active when removed from the influence of nearby repressive effects. We reasoned that these regions would contain sequence motifs of TFs active in GM12878, but that these sequence motifs would be less likely to be bound in their endogenous chromatin context, compared with motifs in active states. Indeed, we found that active HiDRA regions from endogenously inactive chromatin states showed similar enrichments in regulatory motif sequence coverage to that of enhancer and promoter chromatin states (Fig. 3c), but substantial differences in their endogenous TF binding (Fig. 3d), consistent with endogenous repression due to their genomic context. These regions were also ~30% more likely to be active in another human tissue, compared with HiDRA-inactive regions (Supplementary Fig. 6), consistent with cell type-specific repression in their endogenous chromatin context.

In addition to the presence of regulatory motifs for known regulators active in GM12878, we sought additional driver elements that may be responsible for the episomal activity of endogenously inactive regions. In particular, we considered the presence of long-terminal-repeat (LTR) retrotransposons, which have been previously shown to have regulatory activity potential and were enriched in the set of all active HiDRA regions unlike other repetitive elements in the genome (Supplementary Fig. 7a)^13,26^. Indeed, we found that active HiDRA regions from endogenously inactive regions showed substantial enrichment for LTR retrotransposons. In fact, quiescent and heterochromatin states were more enriched for LTR retrotransposons than either Enhancer or Promoter chromatin states (Supplementary Fig. 7b). These regions are endogenously inactive despite their seeming regulatory activity potential, likely due to the effect of repressive chromatin in their endogenous loci. As LTRs are motif-rich and often act as the substrate for recently evolved enhancers, these endogenously inactive but episomally-active HiDRA regions may represent a reservoir for the emergence of new regulatory elements^2^.

### High-resolution mapping of regulatory activity with HiDRA

**Fig. 4:**
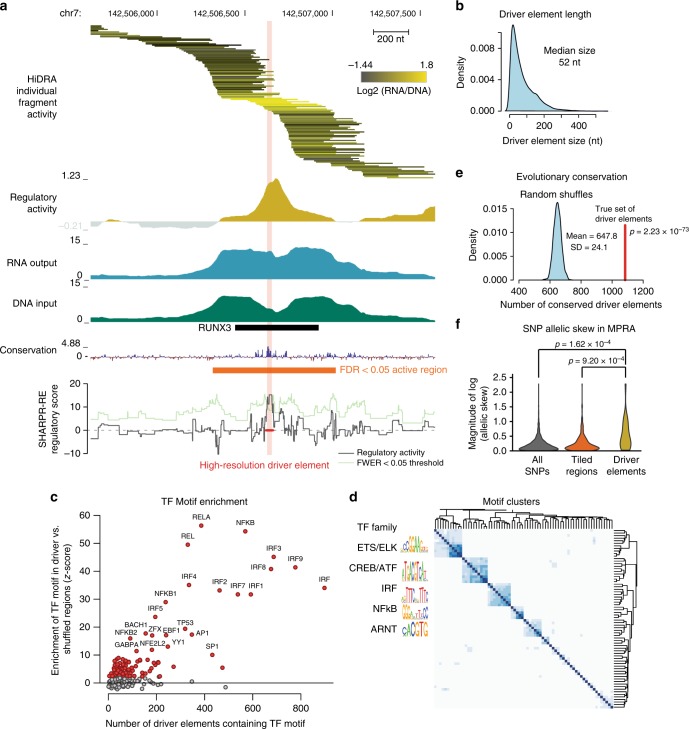
High-resolution mapping of transcriptional regulatory elements with SHARPR-RE. **a** Example region used in high-resolution mapping. Fragment activity shown on log2 scale with two fragments with highest and lowest activity removed for color scale to avoid outliers. The transparent red bar indicates the driver element identified at the regional FWER < 0.05. **b** Size distribution of driver elements. **c** Enrichment of immune-related TF motifs in driver elements compared with shuffled driver elements within tiled regions. **d** TF motifs enriched in driver elements cluster into groups of co-occurring motifs, suggesting diversity of TF motifs involved in transcriptional regulatory activity. **e** Significantly more driver elements are evolutionary conserved compared to shuffled driver elements within tiled regions. Evolutionary conservation cut-off chosen as conservation score for top 5% of shuffled regions (*p *= 2.23 x 10^–73^ vs. Gaussian distribution estimated by random shuffling of driver element positions in tiled regions). **f** SNPs within driver elements have significantly greater allelic skew by MPRA (Tewhey et al.^27^) compared with those within tiled regions or across the genome. *P* value calculated by Mann-Whitney *U* test

We sought to exploit the highly overlapping nature of tested HiDRA fragments to increase the resolution of regulatory inferences by exploiting subtle differences between neighboring fragments that only overlap partially. As an example, we considered a 3 kb region on chromosome 7 that is covered by 134 HiDRA fragments with distinct start and end positions. When we examine every fragment in this region, we observed that fragments overlapping a known RUNX3 motif showed substantially higher regulatory activity (Fig. 4a). This motif is bound by the RUNX3 protein in GM12878 cells and shows increased evolutionary conservation (Fig. 4a)^7^. These properties suggest that the driver regulatory nucleotides within this region are tightly concentrated surrounding the RUNX3 motif, and that on the global level the differential activity of HiDRA-tested segments should enable us to systematically discover these driver nucleotides in an unbiased way based on the relative activity of fragments that do or do not overlap them.

As part of our development of Sharpr-MPRA^13^, we had previously developed the SHARPR algorithm (Systematic High-resolution Activation and Repression Prediction from Reporter assays), a graphical probabilistic model that inferred high-resolution activity from MPRA tiling experiments by reasoning about the differential activity of partially-overlapping microarray spots. Intuitively, SHARPR allowed us to transform measurements from the 145-bp resolution of individually tested tiles to the 5-bp resolution of the offset between consecutive tiles. The SHARPR algorithm relies on synthesized oligos that uniformly tile regions at regularly spaced intervals, and thus is not applicable for the random fragmentation nature of HiDRA experiments where both the length and the spacing of neighboring fragments can vary. To address this challenge, we developed a new algorithm, SHARPR-RE (for SHARPR with Random Endpoints), which estimates regulatory scores underlying any set of randomly positioned and variable-length segments, by appropriately scaling the segments by their varying lengths, and enabling inferences at variable-length offsets between them (Supplementary Methods).

Applying the SHARPR-RE algorithm to the RUNX3 example above, we found that the 3 kb region was narrowed down to a single “driver” element of 27 nt (Fig. 4a). These captured the known RUNX3 motif shown experimentally by ChIP-seq to be bound by the RUNX3 regulator in GM12878^7^, and also the independently determined high-resolution region of evolutionary conservation, even though neither line of evidence was used in our inferences.

Across all ~32,000 “tiled regions” that are covered by at least 10 unique HiDRA fragments (Fig. 1b, Supplementary Fig. 8), SHARPR-RE predicted ~13,000 driver elements of median length 52 nt, using a regional family-wise error rate of 5% (Fig. 4b, see Supplementary Methods and Supplementary Data 2 and 3). The resolution with which driver elements could be resolved increased with the number of unique HiDRA elements spanning a tiled region, reflecting both the increased number of breakpoints in densely tiled regions, and the increased discovery power afforded by the SHARPR-RE algorithm. Regions tiled by 40 or more fragments showed ~20 nt resolution (Supplementary Fig. 9). Regions tiled by fewer fragments (10–20) showed lower resolution (~50 nt), but resolution only increased to ~18 nt with higher fragment density, suggesting the minimum size of driver elements detectable by the HiDRA assay that result in regulatory activity is only slightly longer than individual regulatory motifs. Similar to active HiDRA regions, driver elements were also mostly distal from annotated TSS regions, and were preferentially found in endogenously active chromatin states (active promoters, TSS flanking, and active enhancer regions, Supplementary Fig. 10).

Compared with a background of all tiled regions, which are specifically enriched for GM12878 regulatory regions, we found that predicted driver nucleotides were significantly more enriched for regulatory motifs than shuffled controls (obtained by randomly shuffling driver element positions within the same set of tiled regions with at least 10 unique HiDRA fragments). The enriched motifs consisted of regulators known to be active in GM12878, including several critical B-cell and immune transcription factor including NF-kB and the IRF family (Fig. 4c). A total of 98 motifs were enriched in driver elements (FDR < 0.05 vs. random shuffling of driver elements in tiled regions, see Methods), clustering into several distinct groups with little overlap between groups, suggesting a wide range of distinct transcription factors act to regulate GM12878 gene expression (Fig. 4d). We also found that driver nucleotides are significantly more likely to be evolutionarily conserved across vertebrates than randomly shuffled controls in tiled regions (Fig. 4e), with ~1080 driver elements overlapping conserved regions, compared to only ~650 expected by random shuffling of driver elements within tiled regions (*p* = 2.23 × 10^–73^). Driver elements are also more evolutionarily conserved than equally sized segments residing directly upstream or downstream (Supplementary Fig. 11a), supporting the biological importance of the high-resolution inferences.

We also validated our high-resolution predictions using an independent high-resolution experimental method based on MPRA array synthesis and high-resolution tiling (Sharpr-MPRA^13^). As the original SHARPR algorithm does not include the functionality to call discrete driver elements, we compared Sharpr-MPRA activity scores within driver elements identified in this study compared with equally sized segments shifted upstream and downstream. We found that HiDRA driver elements are much more likely to show Sharpr-MPRA activity than these shifted segments. Sharpr-MPRA activity scores peaked for HiDRA driver elements, and were lower in flanking regions (256 regions tested in both HepG2 and K562, Supplementary Fig. 11b), supporting the functional importance of HiDRA driver nucleotides (Supplementary Fig. 11b, left panels). The agreement was stronger for Sharpr-MPRA scores in K562 than HepG2 (Fig. S11b3), consistent with its higher similarity to GM12878. Specifically distinguishing accessible DNA sites based on their motif content (and thus the trans-acting TFs predicted to target them), we found that predicted targets of K562 and HepG2 TFs that are also expressed in GM12878 showed even higher Sharpr-MPRA scores, whereas targets of TFs that are not expressed in GM12878 showed nearly complete loss of any enrichment signal (Supplementary Fig. 11b, right panels), thus providing a mechanistic explanation for their similarity in activity.

We also evaluated whether genetic variants in predicted driver nucleotides are more likely to result in differential activity between the two alleles, compared with other genetic variants. We used the results of an independent experimental study that quantified allelic enhancer activity for 4335 single-nucleotide polymorphisms (SNPs) across the genome^27^, of which 24 overlap driver elements identified by our assay. Genetic variants inside driver elements indeed showed significantly stronger allelic skews compared with all variants tested by MPRA (*p* = 1.62 × 10^–4^, Mann–Whitney *U* test), and also compared with all tested variants inside HiDRA-tiled regions but outside driver elements (*p* = 9.20 × 10^−4^, Mann–Whitney *U* test) (Fig. 4f), supporting the functional importance of our predictions, and the high-resolution nature of our driver elements.

Taken together, these results indicate that our high-resolution inferences are biologically meaningful and can help pinpoint driver nucleotides among larger regions.

### Characterization of GWAS SNPs affecting enhancer activity

**Fig. 5:**
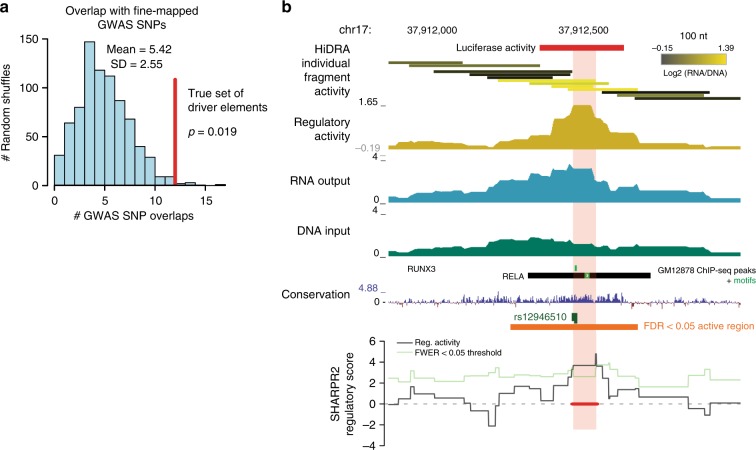
High-resolution driver elements are enriched for fine-mapped GWAS SNPs. **a** Driver elements overlap more GWAS fine-mapped SNPs associated with 21 human immune-related complex traits than randomly shuffled regions. *p*-value calculated empirically by random shuffling of driver element positions within tiled regions. **b** Example locus at rs12946510 that overlaps a high-resolution driver element. Highlighted segment indicates the driver element identified at the regional FWER < 0.05. Red bar at top corresponds to region with luciferase activity as demonstrated by Hitomi et al.^32^

We next sought to use our predicted active regions and driver nucleotides to gain insights into non-coding variation, as past work has demonstrated that disease-associated variants are preferentially localized to regulatory elements^4,28,29^. We studied the overlap between genetic variants associated with immune disorders and our high-resolution predicted driver nucleotides. Even though driver nucleotides only cover 0.032% of the genome, we found 12 cases where they overlap fine-mapped SNPs associated with 21 immune-related traits^30^ predicted to be causal (~5 expected by chance inside tiled regions, *p* = 0.012 vs. random shuffling of driver elements within tiled regions, Fig. 5a). For example, we predict a 76-nt driver element overlapping rs12946510 in the IKZF3 locus associated with multiple sclerosis in a tiled region of 3 kb (Fig. 5b), suggesting this may be the causal variant. The SNP overlaps a 76-nt driver element that contains a RUNX3 motif and a RELA motif, both bound by the respective TFs in GM12878^7^. Indeed, rs12946510 is predicted to be causal based on genetic fine-mapping^30^, with a posterior probability of 0.314 of being causal with the next strongest signal showing only 0.067 posterior probability. rs12946510 is also an eQTL for the nearby IKZF3 gene^30,31^, and was recently shown to disrupt enhancer activity for the surrounding 279-nt region using a luciferase reporter assay^32^, consistent with our prediction that rs12946510 is a causal SNP.

**Fig. 6:**
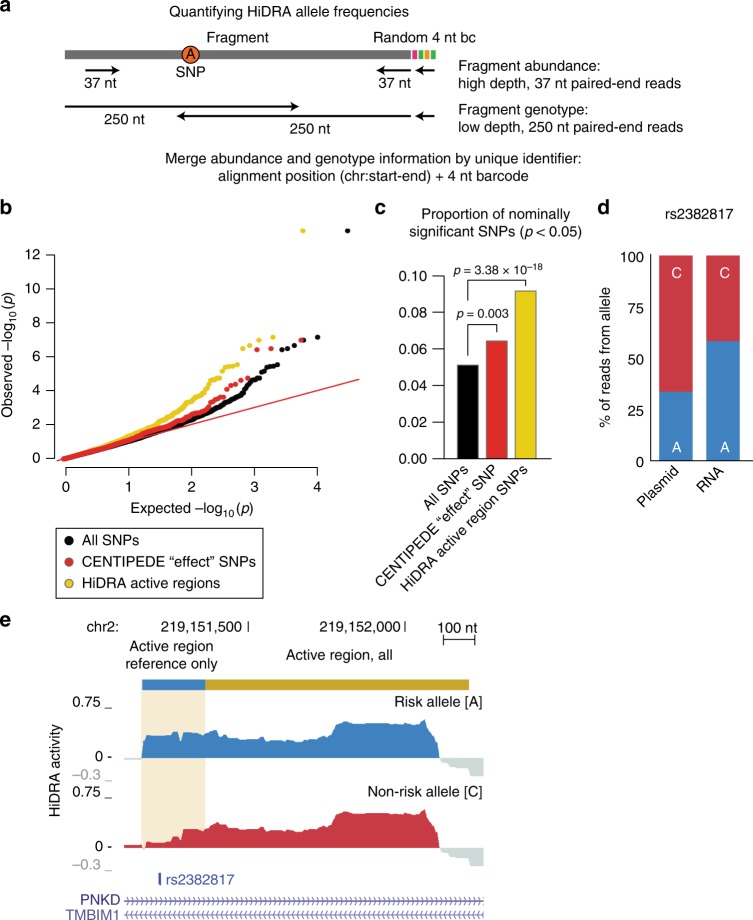
Identification of human genetic variants that alter HiDRA activity. **a** Overview of genotyping approach for HiDRA fragments. HiDRA fragments were originally quantified at high-depth using 37 nt paired-end reads. At this read length the allele composition of fragments is mostly unobserved. As every HiDRA fragment has a unique identifier (genomic alignment position and random 4 nt barcode), long-read re-sequencing of the HiDRA library can assign SNP genotypes to fragments that were previously quantified for activity using short reads. **b** *q*-*q* plot for allelic imbalance at SNPs covered by HiDRA fragments. CENTIPEDE “effect” SNPs were identified by Moyerbrailean et al.^34^. **c** “Effect” SNPs and SNPs within HiDRA active regions are more likely to be nominally significant for allelic imbalance. *p*-values from Fisher’s exact test. **d** The A allele of rs2382817, a SNP associated with inflammatory bowel disease, is more active in the HiDRA assay than the C allele. **e** Alelle-specific HiDRA activity signal tracks for rs2382817

To recognize regions that showed differential activity between risk and non-risk alleles of common genetic variants, we first inferred the genotype of all RNA fragments profiled. As HiDRA is a sequencing-based assay, where the expression of reporter genes is quantified based on the number of sequencing reads, allele-specific differences in HiDRA activity between risk and non-risk haplotypes should be detectable in principle by using heterozygous positions to distinguish reads coming from the paternal or the maternal allele. In practice, however, HiDRA fragments are much longer (~337 median length) than the typical sequencing reads we used for quantification (37 nt, paired end), and thus 78% of genetic variants will not be covered by our sequencing reads (if they fall in the inner ~260 nt not captured by our paired-end sequencing). To overcome this limitation and to determine allele-specific activity scores for all our fragments, we used low-depth re-sequencing of our input library using long reads, thus revealing the genotype associated with each start/end position in our library (Fig. 6a). We augmented this information with 4-nt random i7 barcodes that were added by PCR during the initial HiDRA library construction, thus ensuring that the [start, end, i7] triplet is almost guaranteed to be unique, by resolving the cases where both start and end positions are identical between paternal and maternal alleles. This strategy enabled us to resolve the genotype of all previously quantified HiDRA fragments without having to sequence both the plasmid and RNA libraries to full length at high depth, which would be too costly.

In a proof-of-concept analysis to assess the ability of HiDRA to detect allelic activity, we applied this approach systematically to all heterozygous positions known in the genotyped GM12878 cell lines. We found ~180,000 heterozygous SNPs that were represented by at least one HiDRA fragment at either allele in our library. Detection of allelic activity with random fragmentation is subject to confounders, as fragments carrying the maternal or paternal allele of a SNP may also differ at their start and end positions, which may result in activity differences independent of SNP effects (Supplementary Fig. 12). To minimize such effects, we only compared fragments with 90% mutual overlap, and with driver elements at least 25 nt from fragment ends. Additionally, statistical power to detect allelic differences may be limited for many SNPs. We also only consider SNPs that have >20 read coverage for both fragments (reference and non-reference alleles). In total, ~16,000 SNPs remained after applying these three filters. At an uncorrected nominal *p*-value cutoff of 0.05, we found 880 “allelic” HiDRA SNPs where paternal and maternal alleles showed differences in activity, 25 of which had a corrected FDR < 0.1 (beta-binomial model^33^, Supplementary Data 4). The corresponding SNPs in these 880 allelic HiDRA regions were more frequently found in HiDRA active regions and more frequently predicted to have strong regulatory effects in open chromatin regions by an independent study^34^ (Fig. 6b, c), suggesting they are biologically meaningful. As an example, we found that rs2382817, a SNP associated with inflammatory bowel disease^31^ (GWAS *p* = 1.13 × 10^–13^), shows differential HiDRA activity between paternal and maternal alleles. The risk allele shows increased regulatory activity upstream of a HiDRA-annotated active region (nominal *p* = 8.7 × 10^–4^, FDR = 0.25, *p*-values from QuASAR-MPRA, Fig. 6d, e), illustrating the possibility of using HiDRA to detect SNPs with allelic effects on regulatory activity.

These results indicate that HiDRA can help shed light on disease-associated variants, by either narrowing down the set of candidate causal SNPs using our high-resolution driver nucleotide inferences, or by directly observing differential activity between risk and non-risk alleles using allele-specific activity inferences.

## Discussion

In this paper, we introduced a high-throughput experimental assay, HiDRA, for testing transcriptional regulatory activity across millions of DNA fragments, and for inferring high-resolution driver elements within them. In the experimental component of HiDRA, we capture regions of open chromatin, size selecting them, and inserting them downstream of transcription start in an episomal reporter construct, thus driving their own transcription and serving as their own barcodes (ATAC-STARR-seq). By concentrating the signal on open-chromatin regions, HiDRA enables high-resolution inference of driver nucleotides within these regions using a machine-learning model (SHARPR-RE), by exploiting subtle differences in the reporter activity driven by partially overlapping tested fragments. We refer to the combined SHARPR-RE-ATAC-STARR-seq method as the “High-resolution Dissection of Regulatory Activity”, or HiDRA. By capturing putative regulatory regions directly from open chromatin regions, HiDRA has the advantage of foregoing oligonucleotide synthesis, and thus enabling testing of much longer fragments, and testing many more regions in a single experiment.

We applied HiDRA on the GM12878 lymphoblastoid cell line, revealing a global map of regulatory elements and their sequence-driven effects on transcription. We showed that HiDRA provides a quantitative assay with strong sensitivity and specificity, compared to low-throughput luciferase assays. We showed that fragments with the strongest activity show endogenous promoter and enhancer signatures, contain motifs for immune transcription factors, and show in vivo binding by immune regulators. We also showed that driver nucleotides inferred by our high-resolution mapping are enriched for evolutionarily-conserved regions, known regulatory motifs for immune regulators, and for genetic variants associated with immune traits. We also showed that long-read resequencing of the HiDRA library can distinguish allele-specific activity of risk vs. non-risk fragments derived from heterozygous loci associated with disease, enabling directionality-of-effect inference and providing mechanistic insights on disease loci.

Currently, the only other method that enables the high-resolution dissection of thousands of putative regulatory regions is Sharpr-MPRA^13^. Both Sharpr-MPRA and HiDRA seek to map regulatory regions at high resolution, but they differ in several key respects: Sharpr-MPRA selects regions based on prior computational predictions, requiring microarray-based synthesis. This in vitro synthesis step limits the number of nucleotides and regions tested by Sharpr-MPRA (4.6 Mb in 15,720 regions for Sharpr-MPRA vs. 46.3 Mb in 31,813 regions for HiDRA). Moreover, Sharpr-MPRA uses fixed 5-bp increments (vs. random increments for HiDRA, stemming from the transposase fragmentation pattern), 145-bp fragments (vs. random lengths for HiDRA, 335-bp on average), and Sharpr-MPRA tests elements upstream of the TSS using 3′ UTR barcodes (vs. testing elements downstream, thus enabling them to serve as their own barcodes).

The ATAC-STARR-seq step in HiDRA has many benefits compared with regular STARR-seq without fragment selection. ATAC-STARR-seq preferentially tests the activity of fragments derived from open chromatin, and the HiDRA library we developed achieves 130–220X more highly covered regulatory elements than random genome fragmentation (Fig. 1c). This facilitates both higher confidence discovery of regulatory elements, and the high-resolution dissection of regulatory elements by comparing the relative activity of partially overlapping fragments. This latter application using our newly developed SHARPR-RE algorithm permits the genome-wide mapping of high-resolution driver elements, and would not be possible with regular STARR-seq unless extremely complex fragment libraries are used (at least 15- or 20-fold more complex than this current library). HiDRA can therefore be feasibly and readily applied across a wide range of tissues for high-resolution mapping without the limitations that regular STARR-seq would entail (e.g., > 10^9^ cells per tissue, and high depth of sequencing to quantify a library with ~20-fold more fragments).

The HiDRA approach is general and can be readily adapted for other applications. While we performed our study using ATAC regions from the GM12878 cell line and re-transfection of constructs in the same cell line, the approach is applicable to any cell type, and to combining of different source and target cells. For example, libraries could be generated from limited patient tissue, or a pool of multiple donors to increase heterozygous loci, and subsequently transfected into a relevant immortalized cell line that can be easily grown to high cell quantities. Here, we used transfection to introduce episomal plasmid reporters, but lentiviral methods can be used in cell lines with lower transfection efficiencies. We chose non-integrating episomal reporters to focus more directly on sequence function independently of its broader chromatin context, however the HiDRA approach can be used with an integrating lentiviral vector to also incorporate the effect of chromosomal context, with the understanding that previous analyses used up to ~100 integration sites per tested element to accurately quantify activity^35^. Moreover, integrating reporter techniques can require cell sorting using fluorescent reporter activity, which limits the number of constructs that can be tested^36^.

Several improvements are possible on the existing HiDRA experimental approach. In addition to the minimal SCP1 promoter, the STARR-seq vector has a second active promoter at the bacterial ORI site, whose transcripts are both more abundant and have stronger signal-to-noise ratios at regulatory elements^25^. Thus, to improve detection of modestly-active enhancer elements, future studies may forgo the SCP1 promoter altogether and utilize the ORI site exclusively. Zabidi et al. previously demonstrated that different promoters in *Drosophila* STARR-seq experiments will uncover different classes of enhancer elements (e.g., developmental vs. housekeeping)^37^. Based on Muerdter et al., the pSTARR-seq_human plasmid employed here drives transcriptional initiation from two promoter elements: the SCP1 synthetic core promoter and the bacterial plasmid origin of replication, suggesting that the original pSTARR-seq_human plasmid may identify more active enhancer elements than newer STARR-seq plasmid backbones lacking SCP1^25^. Future HiDRA studies can also increase the diversity of promoter sequences tested to improve the detection of subclasses of enhancer elements. In addition, transfection can induce interferon response in some cell types, which can alter the transcriptional landscape if they do not already express interferon, as is the case for HeLa-S3 cells^25^. The GM12878 cell line used here already expresses interferon-stimulated genes such as IFIT1, ISG15, and IRF7, similarly to other EBV-transformed lymphocyte lines, and thus interferon response does not greatly alter the regulatory landscape. For other cell lines, we recommend following the recommendation by Muerdter et al. and inhibiting relevant IRF signaling kinases during plasmid transfection^25^.

The HiDRA approach can also be specifically tuned for mapping differential allele activity in regulatory regions associated with human disease SNPs from GWAS. At the library construction stage, capture probes can be used to further increase coverage at known polymorphic SNPs of interest, thus increasing fine-mapping resolution, facilitating comparison of fragments with alternate alleles but matching start and end positions, and increasing statistical power to detect differential activity between risk and non-risk alleles. To increase the number of heterozygous sites, future studies can pool cells or tissue from multiple individuals, thus ensuring polymorphic representation of many disease-associated loci. For cancer mutations and other somatic mutations, HiDRA may also be applied to pools of tumor samples, or pools of disease tissue, to identify variants that alter regulatory activity and gene expression^38^. For systematic mutagenesis, HiDRA libraries can also processed to introduce new mutations through error-prone PCR or introduction of mutagens during amplification.

We also envision modifications of the HiDRA assay presented here for testing specific subsets of the genome. A modified HiDRA assay may be used to enrich for fragments from active regulatory regions, by coupling HiDRA with a fragment capture technology similar to those used in Capture Hi-C to selectively test a subset of enhancers or promoters at higher resolution while retaining the advantages of having larger fragment sizes and high library complexity^39^. To test regions associated with specific chromatin states, capture could be performed using the output of chromatin immunoprecipitation experiments (ChIP) using histone modifications, thus preferentially sequencing genomic regions that were also pulled down with ChIP. Finally, the SHARPR-RE high-resolution mapping algorithm developed here can be applied to perform high-resolution mapping of genomic regions for high-complexity libraries with sufficient fragment density (e.g., testing of individual large regions using bacterial artificial chromosome clones with “BAC-STARR-seq”^16^, or high-resolution mapping of transcription factor binding sites with ChIP-STARR-seq^19^). We envision that HiDRA and such modified approaches can be used to quantify the transcriptional regulatory landscape of DNA sequences for a variety of tissues from multiple organisms.

## Methods

### HiDRA library construction

We performed 16 ATAC-seq reactions on 50,000 GM12878 cells, each using a modified protocol based upon Buenrostro et al.^21^ (Supplementary Note 1). Initial steps of ATAC-seq (cell collection, lysis, and Tn5 digestion) followed the protocol in Buenrostro et al.^21^: each batch of 50,000 cells was collected by spinning at 500 g for 5 min in a 4 °C cold room, washed with 50 µL of 1X PBS, and resuspended in ATAC-seq lysis buffer (10 mM Tris-HCl, pH 7.4, 10 mM NaCl, 3 mM MgCl_2_, 0.1% IGEPAL CA−630); a pellet was collected by spinning at 500 g for 10 min, and was resuspended in 25 µL of TD buffer (Illumina #FC-121–1030), 2.5 µL Tn5 transposase (Illumina #FC-121–1030), and 22.5 µL of dH_2_O; the transposition reaction proceeded for 30 min at 37 °C on a shaker (300 rpm). Tn5-fragmented DNA was cleaned up using a MinElute PCR purification kit (Qiagen #28004, four reactions per column eluted in 20 µL of EB buffer) and the resulting 80 µL of eluate was split into 16 PCR reactions (Supplementary Note 2). PCR was performed using custom HPLC-purified primers (F: 5′-TAGAGCATGCACCGGCAAGCAGAAGACGGCATACGAGATNNNNATGTCTCGTGGGCTCGGAGATGT-3′, R**:** 5′-GGCCGAATTCGTCGATCGTCGGCAGCGTCAGATGTG-3′, where NNNN corresponds to a random 4 nt i7 barcode sequence) and NEBNext Ultra II Q5 DNA polymerase master mix (NEB #M0544L). Thermocycler conditions were: 65 °C for 5 min, 98 °C for 30 s, 8 cycles of: 98 °C for 10 s and 65 °C for 90 s. PCR reactions were pooled and cleaned up with a Qiagen MinElute PCR purification kit (two PCR reactions per column eluted in 20 µL of EB buffer) and run on a 1% agarose E-Gel EX with SYBR Gold II stain (Thermo Fisher #G402001). Size selection of ATAC-seq fragments was performed by gel excision using a razor blade to select fragments between 150–500 nt. Gel slabs were pooled into < 300 mg groups and DNA was purified using a MinElute Gel Extraction kit (Qiagen #28604), and eluted in 20 µL of buffer EB per column following modified guidelines described in Box 2 of Taiwo et al.^40^. The resulting size-selected ATAC-seq fragment library was treated with an anti-mitochondrial DNA CRISPR/Cas9 library following the protocol outlined in Montefiori et al. using 10X excess of Cas9 protein (Supplementary Note 3)^41^. We cleaned up the reaction with a Qiagen MinElute PCR purification kit and split into eight PCR reactions for a second round of PCR using the same conditions and primers described above. PCR products were cleaned up using two rounds of AMPure bead selection (0.8X ratio of beads to input) to size-select against small (<150 nt) fragments, eluted in 40 µL of dH_2_O and quantified using a Qubit dsDNA HS Assay kit (Thermo Fisher #Q32854).

The pSTARR-seq_human plasmid used for generating the plasmid library was a gift from Alexander Stark (Addgene plasmid #71509). The linear backbone used for the subsequent cloning steps was generated by digesting 4 µg of circular pSTARR-seq_human for 4–6 h with AgeI and SalI restriction enzymes (NEB #R3552S and R3138S), followed by gel excision under a dark reader transilluminator (Clare Chemical #DR22A) to extract a linear 3.5 kb fragment corresponding to the human STARR-seq plasmid backbone. We performed cloning of the fragment library into the plasmid backbone approximately following the Methods section from Arnold et al.^16^. For each library, we performed 20 individual InFusion HD cloning reactions (Takara Bio #638911) using a 3.5:1 molar ratio of insert to vector backbone, following manufacturer’s instructions (Supplementary Note 4). Each group of five InFusion reactions was collected and cleaned up using the Qiagen MinElute Enzymatic Reaction cleanup kit, eluted in 10 µL of dH_2_O, and transformed into four 20 µL aliquots of MegaX DH10B T1R electrocompetent bacteria. The bacteria were thawed on ice for 10 min and mixed with eluted DNA (five InFusion reactions per 100 µL of bacteria). In total, 22 µL of bacteria/DNA mixture were pipetted into a 0.1 cm electroporation cuvette (Thermo Fisher Scientific #P41050) and tapped repeatedly against a hard surface to remove bubbles. Cuvettes were electroporated using a Bio-Rad Gene Pulser Xcell Microbial Electroporation System (Bio-Rad #1652662) using the conditions: 2.0 kV, 200 Ω, 25 µF (Supplementary Note 5). For high-yield transformations, we observed electroporation time constants between 4.8 and 5.1 ms. After electroporation, bacteria were immediately collected in 750 µL pre-warmed SOC media, pooled, and incubated for 1 h in a 37 °C shaker. After recovery, serial dilutions of bacteria were plated to estimate the number of clones in the library. Recovered bacteria were diluted in 2 L of pre-warmed Luria broth and 100 µg/mL of carbenicillin and grown overnight (8–10 h while shaking). Plasmids were collected from bacteria using the Plasmid Plus MegaPrep kit (Qiagen #12981) following manufacturer’s instructions. Plasmid concentration was quantified using a Nanodrop One machine (Thermo Scientific) and diluted to a 3 µg/µL concentration for subsequent transfection steps. To ensure plasmid library quality and diversity, a small aliquot of the fragment library was amplified by PCR using i5 and i7 primers, run on an Illumina MiSeq machine using the 50-cycle v2 kit as per manufacturer’s instructions, and aligned to the human genome to ensure correct complexity and sufficient proportions of reads within predicted transcriptional regulatory elements (Supplementary Note 6, see subsequent Methods sections for details on processing of sequencing libraries).

### Cell culture and transfections

GM12878 cells were obtained from the Coriell biorepository and grown in RPMI 1640 Medium with GlutaMAX Supplement (Thermo Fisher #61870127), 15% fetal bovine serum (Sigma Aldrich #F2442), and 1% pen/strep at a density of between 2 × 10^5^ and 1 × 10^6^ cells/mL with regular media changes every 2–3 days. Approximately 24 h before transfection, GM12878 cells were split to a density of 4 × 10^5^ cells/mL to ensure the presence of actively dividing cells for increased transfection efficiency. For transfection, cells were collected by centrifugation for 5 min at 300 *g*, washed once with pre-warmed PBS, and collected again for 5 min at 300 *g*. PBS was aspirated and cell pellets were re-suspended in Resuspension Buffer R (Thermo Fisher Scientific #MPK10096) at a concentration of 7.5 million cells per 100 µL. DNA was added to cells at a concentration of 5 µg of plasmid per 1 million cells. In total, we transfected five replicates with 120–130 M million cells per replicate using 100 µL tips from the Neon Transfection System at 1200 V with three pulses of 20 ms. Replicate number was chosen based on other high-throughput reporter assay studies (e.g., Vockley et al.^19^ and Tewhey et al.^27^). Transfected cells were immediately recovered in pre-warmed GM12878 media without antibiotic and recovered at a density of 1 × 10^6^ cells/mL for 24 h. In parallel, we performed two transfections of GM12878 cells with a positive control GFP plasmid to assess transfection efficiency using the same conditions.

### RNA isolation and cDNA generation

GM12878 cells were collected 24 h post-transfection, washed twice in pre-chilled PBS (collecting for 5 min at 300 *g*) and RNA was purified using the Qiagen RNEasy Maxi kit (Qiagen #75162) following manufacturer’s instructions and performing the optional on-column DNase treatment step (Qiagen #79254). Poly A+RNA was extracted from total RNA using the Oligotex mRNA Midi kit (Qiagen #70042, two columns per RNA sample), and any remaining DNA was digested with a second DNase treatment step using Turbo DNase (Thermo Fisher #AM2238) following manufacturer’s instructions (Supplementary Note 7). Treated mRNA was cleaned up and concentrated using the Qiagen RNEasy MinElute Cleanup kit (Qiagen #74204). We generated cDNA from mRNA using Superscript III Reverse Transcriptase (Thermo Fisher #18080085) with a gene-specific RT primer located in the 3′ UTR of the sgGFP reporter gene downstream from the inserted fragments (5′-CAAACTCATCAATGTATCTTATCATG-3′). Reverse transcription was performed following manufacturer’s recommendations except with 2 µg of poly A + mRNA and 1 µL of 12.5 µM primer per 20 µL reaction, and extension was performed for 60 min at 50 °C (Supplementary Note 8). Reverse transcription reactions were cleaned up using a MinElute PCR purification kit (Qiagen #28106, two reactions per column) and eluted in 15 µL of pre-warmed buffer EB.

### Library construction and high-throughput sequencing

We performed a qPCR to test the number of cycles needed for amplification of single-stranded cDNA as well as input material of plasmid DNA needed such that both reactions had the same Ct values. We used 1 µL of ssDNA and dilutions of plasmid DNA similar to the method described by Tewhey et al.^27^. qPCRs were performed in 10 µL reactions with all reagents scaled down proportionally from a normal 50 µL PCR reaction (1 µL of DNA, 5 µL of Ultra II Q5 master mix, 0.4 µL of 25 µM primer mix, 0.2 µL of 10X SYBR dye, 3.4 µL of dH_2_O) with thermocycler conditions: 98 °C for 30 s, 20 cycles of: 98 °C for 10 s, 65 °C for 90 s. We proceeded to perform eight regular 50 µL PCR reactions (each scaled up 5X from the 10 µL PCR reactions) using the same thermocycler conditions except using the Ct value for the cycle number (F: 5′-CAAGCAGAAGACGGCATACGAGAT-3′, R: 5′-AATGATACGGCGACCACCGAGATCTACAC[X8]TCGTCGGCAGCGTC-3′, “X8” sequence corresponds to sample barcode, chosen from Illumina Nextera barcode list). PCR reactions were cleaned up using Qiagen MinElute PCR purification kits and balanced for sequencing using the Kapa Library Quantification Kit (Kapa Biosystems #KK4824, Supplementary Note 9).

Each library batch (five transfected RNA biological replicates, five plasmid controls) was sequenced by the Broad Institute Walk-Up Sequencing Facility on four flowcells on a NextSeq 500 machine using the 75-cycle kit as per manufacturer’s instructions for 2 × 37 nt paired-end reads with 2 × 8 nt barcodes.

### Fragment data processing and calling active fragment groups

Reads were labeled by a random 4 nt P7 barcode and an 8 nt P5 barcode for sample ID. Reads were split into the ten samples (five plasmid replicates and five RNA replicates) by P5 barcode and aligned to the human genome (hg19 assembly) using bowtie2 v2.2.9. Alignment files were filtered to (i) keep only aligned fragments, (ii) remove reads mapping to chrM, (iii) select reads passing the -q 30 filter in samtools, and (iv) remove reads aligning to the ENCODE hg19 blacklist regions (Supplementary Note 10). We identified unique fragments using the bamtobed command in BEDTools (v2.26.0) and filtered to keep only fragments between 100 and 600 nt. A diagram illustrating proportion of reads lost to each filter step is available in Supplementary Fig. 13.

In analyzing results from HiDRA, we track the abundance of each individual fragment between the input (plasmid DNA) and output (RNA). We grouped fragments into “fragment groups” by 75% mutual overlap (bedtools v2.26.0, intersectBed command), removed redundant fragment groups and summed counts of all fragments per fragment group. As we detect active fragments by comparing RNA signal to the non-transfected DNA library, we controlled for the possible length-dependent biases in transfection efficiency of plasmids by splitting fragment groups into separate bins of 100nt (100–200 nt, 200–300 nt, etc.) and used DEseq2 (v1.10.1) to identify FDR < 0.05 significantly up-regulated fragment groups in each bin^42^.

### Analysis of active HiDRA regions

All overlap and shuffle analyses performed using the BEDTools suite, v2.26.0^43^. Most colors for plots chosen with guidance from the wesanderson R package (https://github.com/karthik/wesanderson). For chromatin state annotations, we used the 18-state output model generated by the Roadmap Epigenomics Consortium^6^. Active enhancer states were merged from states #9 and #10 (EnhA1 and EnhA2). ATAC-seq peaks positions were obtained from Buenrostro et al.^21^.

Signal tracks: Signal tracks for regulatory activity calculated as (RNA-DNA)/DNA after adding a pseudocount of 0.1 to both plasmid and RNA samples, so that fragments with no activity have a regulatory activity value of 0. Signal tracks were drawn in UCSC Genome browser showing only means (no whiskers) and with 5-pixel smoothing.

Correlation between RNA samples: We show correlations for fragments selected by four different cutoffs of minimum RPM. Pearson and Spearman correlations were calculated on log2-transformed data. Matrix of graphs drawn using layout and grid.arrange functions in R from the gridExtra library. Scatterplot between RNA samples drawn using the hexbinplot function from the hexbin library in R with xbins = 100.

Proximal vs. distal: TSS regions were defined using the UCSC Genome Browser’s Table Browser tool for hg19. Distances to nearest annotated TSS were taken using closestBed tool in the BEDTools2 suite.

TF motif enrichment: We obtained the hg19 TF motif catalog from the ENCODE project^7^. We only considered motifs corresponding to transcription factors expressed in GM12878 (RPKM > 5 using processed GM12878 RNA-seq data from the Roadmap Epigenomics Consortium). TF motifs in driver elements were compared against motifs found in shuffled driver elements within the same set of tested tiled regions (regions with at least 10 HiDRA fragments).

Random shuffling of driver elements: To assess significance of TF motifs, evolutionary conservation and fine-mapped GWAS SNPs in driver elements, we shuffled the positions of driver elements within tiled regions (genomic segments with at least 10 HiDRA fragments) using shuffleBed with the -incl flag to force driver elements to be shuffled within tiled regions. To assess the significance of enrichment, we performed 1000 shuffles of driver elements and calculated *z*-score of true driver elements compared to shuffled driver elements. The *p*-value of this difference was calculated in R from this *z*-score under a normal distribution (two-sided) with mean and standard deviation calculated from random shuffles.

Activity of HiDRA regions in other tissues: We set a lenient definition for active in other tissues as the union of regions annotated in 97 non-GM12878 tissues from epigenome roadmap predicted with 18-state ChromHMM model. For active regions we considered states “TssA” (state #1), “TssFlnkU” (state #3), and “EnhA” (states #9 and 10).

SHARPR-RE activity plots: Tracks were drawn in the UCSC Genome Browser using “Custom Tracks”. Coloring of individual fragments was performed by setting maximum and minimum colors (RGB 0,0,0 and RGB 255,255,0, respectively) to log2(RNA/DNA) values of 3rd lowest and 3rd highest fragments (two strongest and weakest fragments were removed to avoid strong outliers), and scaling colors of all other fragments linearly between these extremes. We chose to include only ChIP-seq bound TF bars for ChIP-seq experiments performed in GM12878 cells by the ENCODE project and where the motif (green bar) overlapped driver nucleotides.

Comparison of driver elements vs. MPRA allelic skew: We use allelic skew data from Supplemental Table S1 from Tewhey et al.^27^. In total, ~39,500 SNPs were tested by Tewhey et al.^27^ for allelic activity, of these 4335 SNPs had enhancer activity in MPRA fragments containing either allele so that allelic skew can be calculated. 3291 SNPs remained after using dbSNP142 and the corresponding RsMergeArch file to assign coordinates for these SNPs. We used this set of 3291 SNPs to assess the degree of allelic skew inside driver elements.

Comparison of driver elements vs. Sharpr-MPRA activity: We used Sharpr-MPRA activity scores from the basepredictions_*_ScaleUpDesign1and2_combinedP.txt files provided by Ernst et al.^13^. We identified the top Sharpr-MPRA activity score per driver element and compared these to activity scores for control, shifted elements.

### SHARPR-RE identification of high-resolution driver elements

See Supplementary Methods for details and more information on SHARPR-RE.

### Read mapping and data analysis for allele-specific activity

We used vcf-consensus (VCFTools) to mask the hg19 genome assembly by replacing heterozygous nucleotides identified by the Illumina NA12878 Platinum Genome with N’s. 250 nt paired-end MiSeq reads were trimmed using cutadapt to remove Illumina primer sequences, mapped to the NA12878-masked hg19 assembly using bowtie2 v2.2.9 (settings: –end-to-end –phred33 –sensitive -p 7 -N 1 –no-unal), and filtered using the steps described above for 37 nt reads. As some long reads have poor quality scores at their 3′ end, we trimmed low quality sequences (quality value < 38) to reduce the proportion of sequencing errors at SNPs that could lead to incorrect allelic assignment of fragments. Fragments were then assigned to a SNP based on genotype at the position. For comparisons of SNP activity, we only considered fragments with 90% mutual overlap to reduce the confounding effect of fragments that differ by both allele and position. We also removed fragments if either end was within 25 nt of a driver element, as in these cases small differences in end position could artifically lead to large effects. After assigning fragment abundances (from high-depth 37 nt PE read sequencing) to each allele of a SNP, we identified SNPs with significant differential activity using QuASAR-MPRA. CENTIPEDE SNPs were identified by Moyerbrailean et al. using an effect-size cutoff of > 3 or < −3, following the cutoffs used by Kalita et al.^33,34^.

### Method considerations and detailed information

Additional information and considerations in applying the method are are provided in the Supplementary Notes.

## Supplementary Information

Supplementary Information

Peer Review File

Description of Additional Supplementary Files

Supplementary Data 1

Supplementary Data 2

Supplementary Data3

Supplementary Data 4

## Data Availability

All high-throughput sequencing data generated by this study has been deposited in NCBI GEO with accession GSE104001. Processed HiDRA plasmid input, RNA output, activity, as well as active fragments and driver elements can be directly visualized on the UCSC genome browser at: https://genome.ucsc.edu/cgi-bin/hgTracks?hgS_doOtherUser=submit&hgS_otherUserNamexinchenw&hgS_otherUserSessionNameHiDRA_GM12878_092617. These bigWig files are additionally available at the NCBI GEO repository (GSE104001). The SHARPR-RE R package is available on CRAN. All other relevant data supporting the key findings of this study are available within the Article and its Supplementary Information files or from the corresponding authors upon reasonable request.
