# Supplementary material for: High-resolution genome-wide functional dissection of transcriptional regulatory regions and nucleotides in human
Source: Nat Commun. 2018 Dec 19;9:5380. doi: 10.1038/s41467-018-07746-1 (PMC6300699; doi:10.1038/s41467-018-07746-1)
Supplement: Supplementary file 1 — Supplementary Information [file 41467_2018_7746_MOESM1_ESM.pdf]

Supplementary Information

High-resolution genome-wide functional dissection of transcriptional regulatory regions in human

Table of Contents

Supplementary Figures 1-13	2
Supplementary Notes	15
Supplementary Methods: SHARPR-RE methodology	17
Supplementary Figures & Supplementary Tables for SHARPR-RE	25

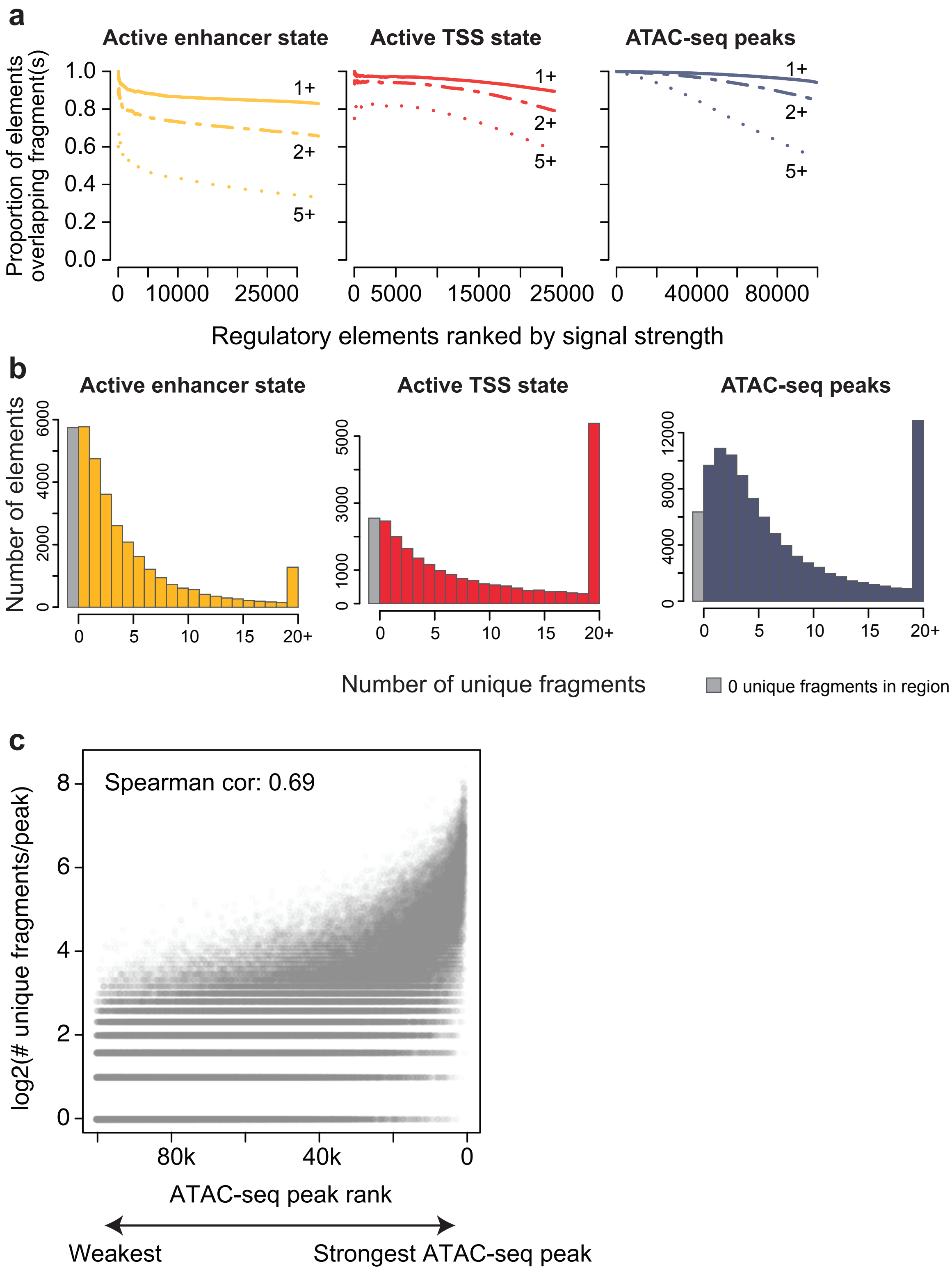

Supplementary Figure 1: HiDRA coverage is greater for highly active regulatory elements. (a) ChromHMM-defined active enhancer and active TSS chromatin states were ranked by H3K27ac signal strength, and ATAC-seq peaks were ranked by density of ATAC-seq reads from Buenrostro et al. (2013). Solid, dashed and dotted lines correspond to coverage with at least 1, 2 and 5 unique HiDRA fragments. (b) Number of unique HiDRA fragments in ChromHMM-defined active enhancer and active TSS elements, and ATAC-seq peaks. (c) Positive relationship between ATAC-seq peak strength and number of unique HiDRA fragments. Discrete bands at bottom of scatterplot represent peaks with 1-6 unique fragments.

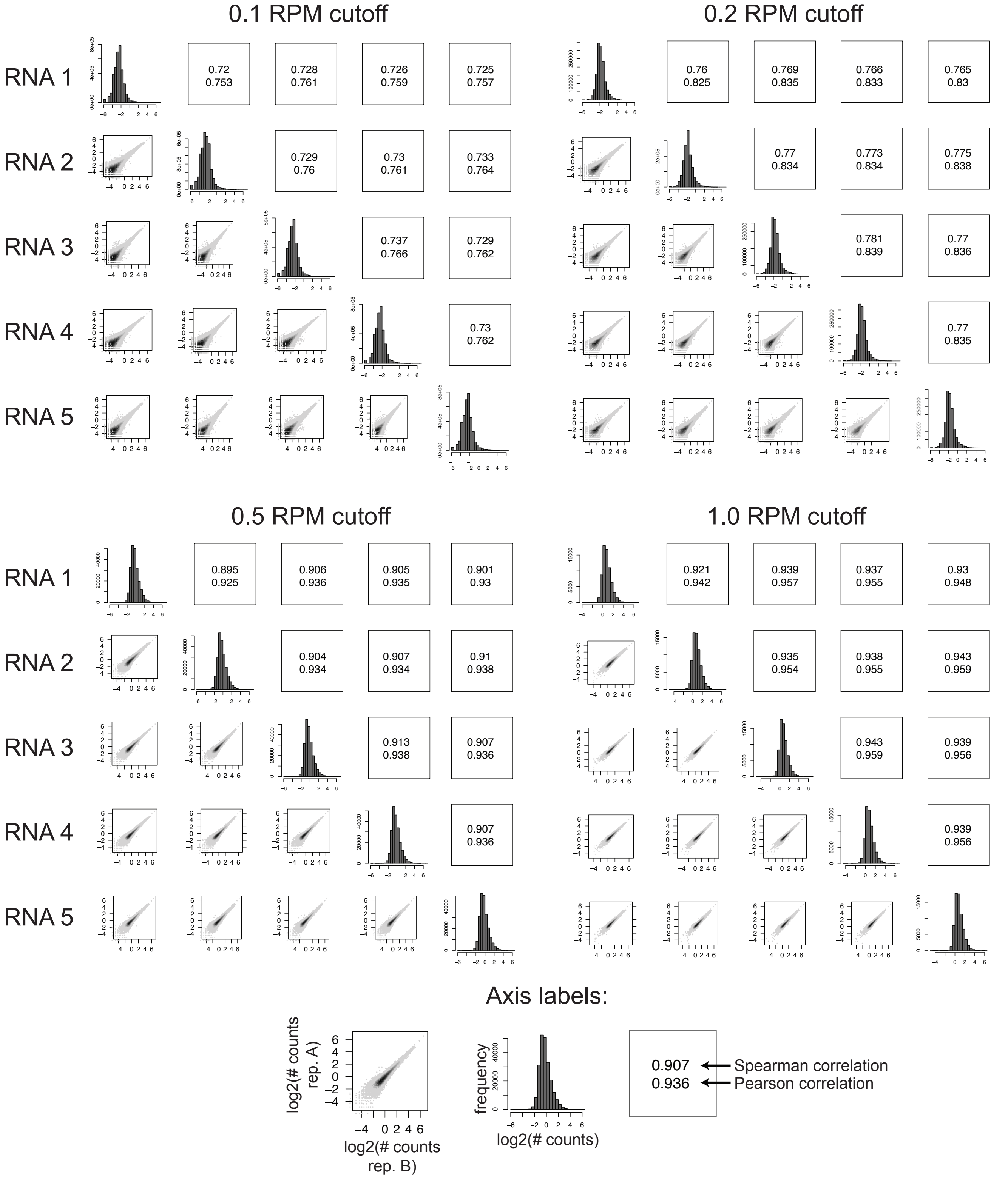

Supplementary Figure 2: Correlation between RNA samples from HiDRA. Correlation is shown at four different RPM cut-offs. Only fragments passing the minimum RPM cut-off in plasmid samples are shown. Unlike gene expression analysis where read counts from many unique fragments are collapsed into one gene expression value, in HiDRA we consider each fragment on its own. Given the high number of unique features in HiDRA, Poisson “shot noise” will decrease correlations between replicates for low RPM cut-offs.

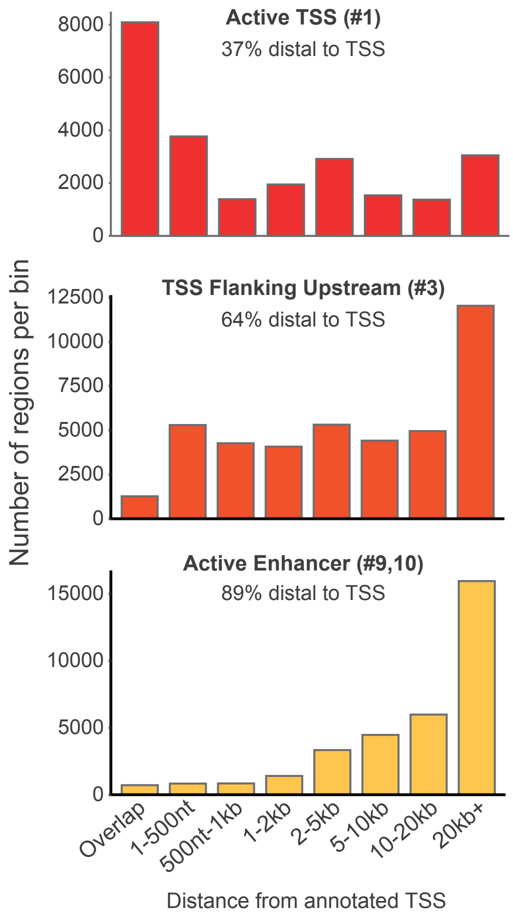

Supplementary Figure 3: Genomic distribution of TSS Flanking Upstream regions. The majority of ChromHMM-predicted TssFlnkUp regions are not near annotated TSSs, but share a similar genomic distribution pattern more similar to predicted active enhancers.

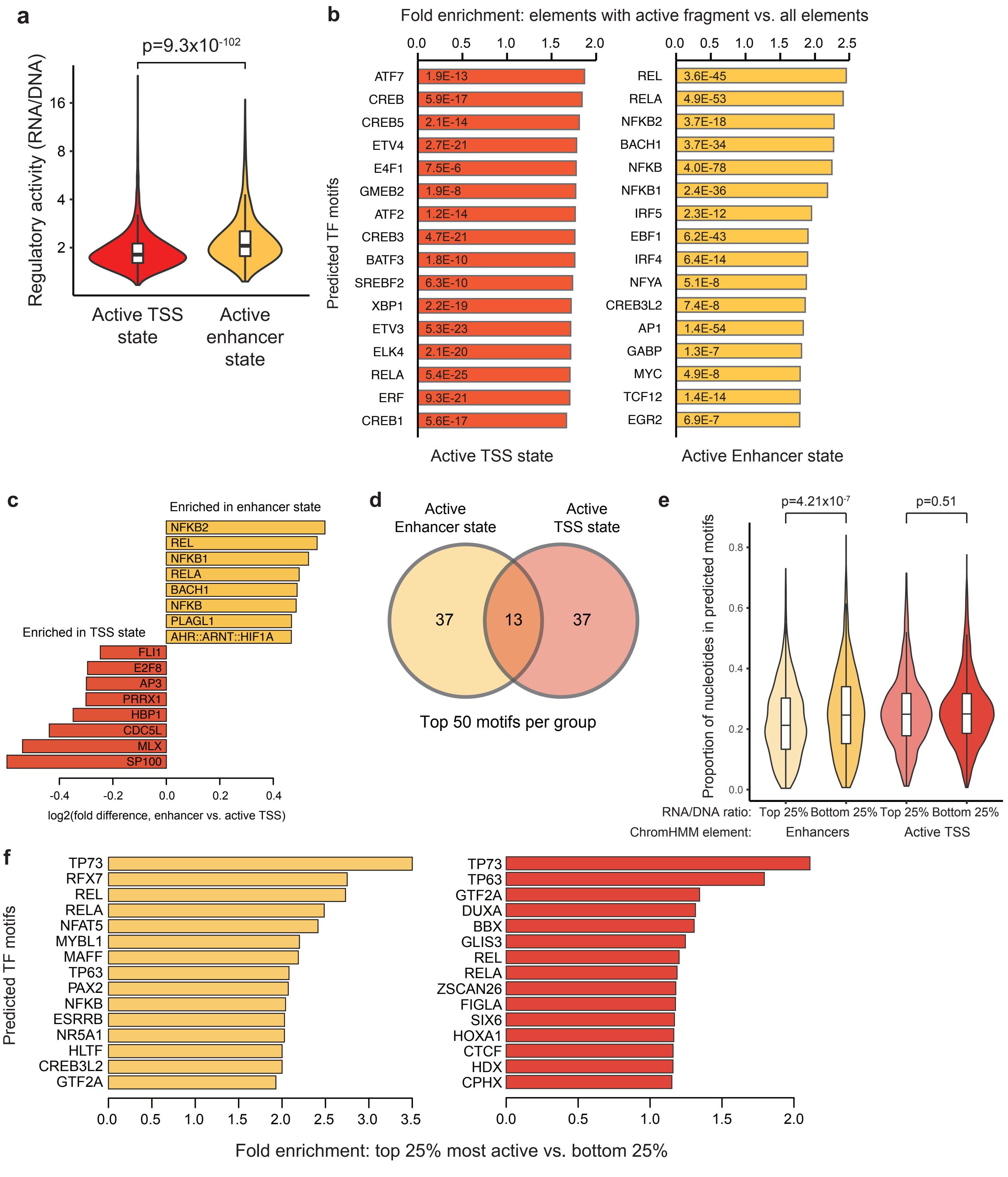

Supplementary Figure 4: Enrichment of motifs in active HiDRA regions. (a) HiDRA fragments within active enhancer state regions have greater regulatory activity than those within active TSS state regions. p-value from Mann-Whitney U test (b) Motif enrichment is calculated separately for active TSS state regions (left) and active enhancer state regions (right) that overlap active HiDRA fragments. Only top 16 motifs are shown for each group after filtering to keep only motifs corresponding to expressed TFs in GM12878 (RPKM>5). Numbers within bars correspond to false discovery rate after Benjamini-Hochberg correction. (c) Top motifs that are differentially enriched in enhancer vs. active TSS states. (d) Top motifs enriched in enhancer and active TSS states are largely distinct from each other (e) Proportion of nucleotides inside motifs for highly and lowly active fragments in enhancers and active TSS regions. p-values calculated by Mann-Whitney U test.
(f) Enriched motifs in highly active enhancer (left) and active TSS (right) regions.

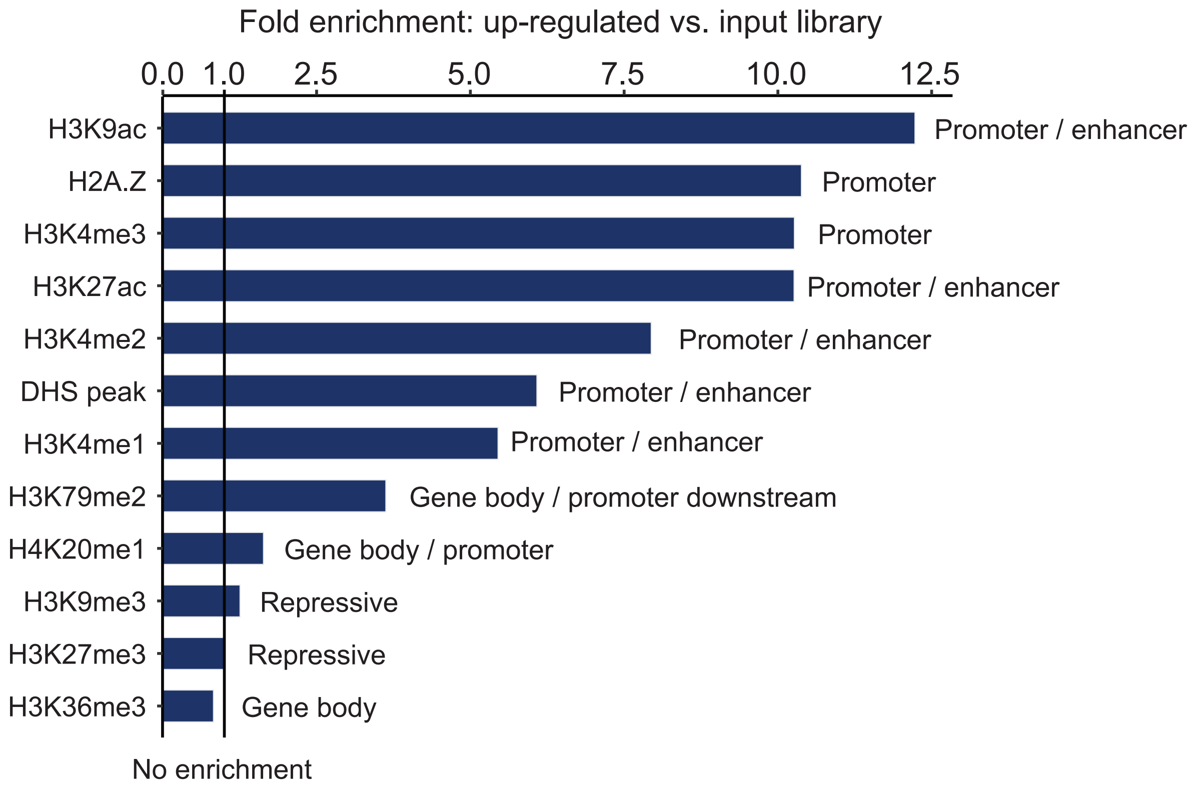

Supplementary Figure 5: Enrichment of histone modifications in active HiDRA fragments. All histone modifications and DHS data were collected from GM12878 cells by the ENCODE or Roadmap Epigenomics projects. Veritical line corresponds to no enrichment (fold enrichment = 1)

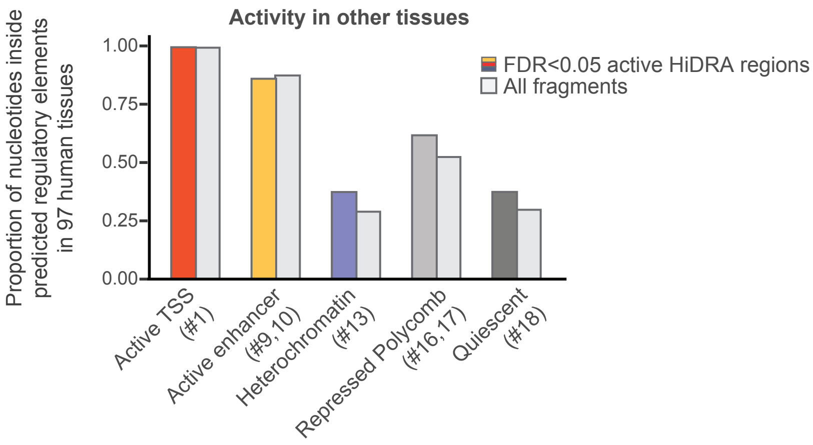

Supplementary Figure 6: Endogenously inactive chromatin states overlapping active HiDRA fragments are more likely to be active in 97 other (non-GM12878) human tissues. No difference observed for endogenously active regions. *Colored bars*, regions from each chromatin state overlapping active HiDRA regions called at FDR<0.05. *Grey bars*, regions from each chromatin state overlapping all HiDRA fragments tested.

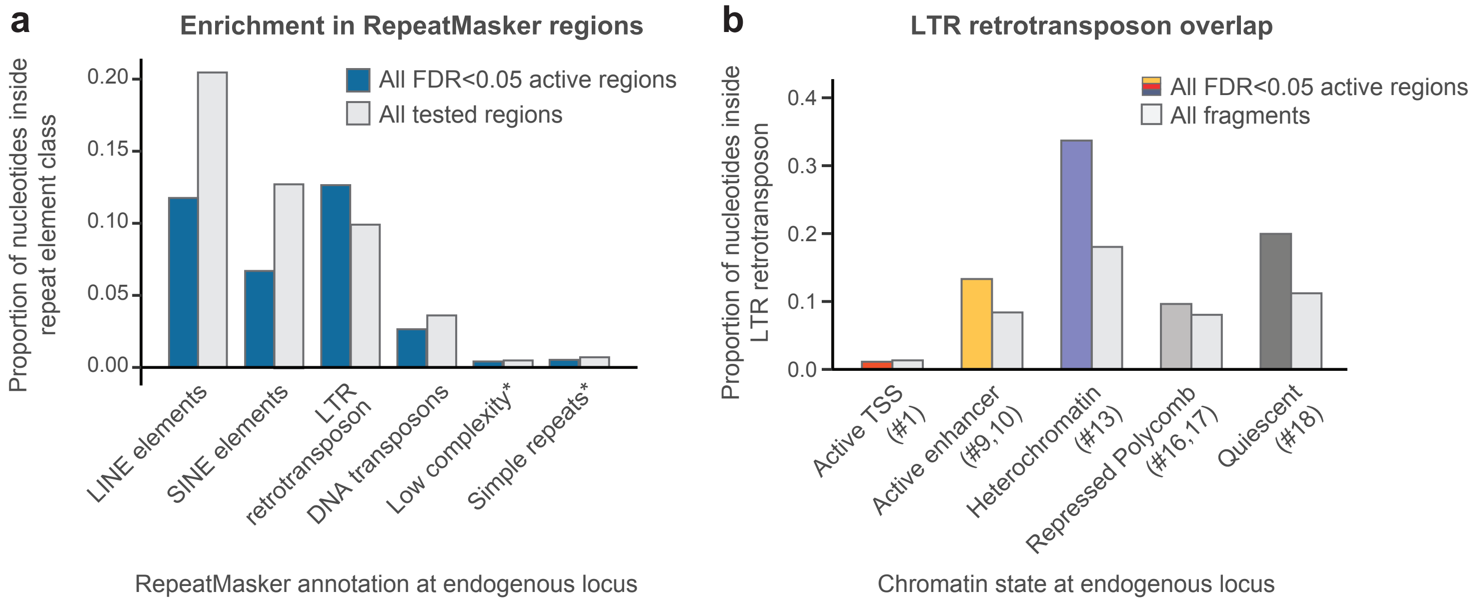

Supplementary Figure 7: LTR retrotransposon repeat elements are enriched within active HiDRA regions in endogenously inactive chromatin states. (a) Elements identified using RepeatMasker annotation of the hg19 human genome. (*) Low complexity and Simple Repeat classes are artificially low due to pre-filtering to remove HiDRA fragments mapping to multiple genomic locations. (b) Endogenously inactive chromatin states overlapping active HiDRA fragments are enriched for LTR retrotransposons compared to endogenously active regions. *Colored bars*, regions from each chromatin state overlapping active HiDRA regions called at FDR<0.05. *Grey bars*, regions from each chromatin state overlapping all HiDRA fragments tested.

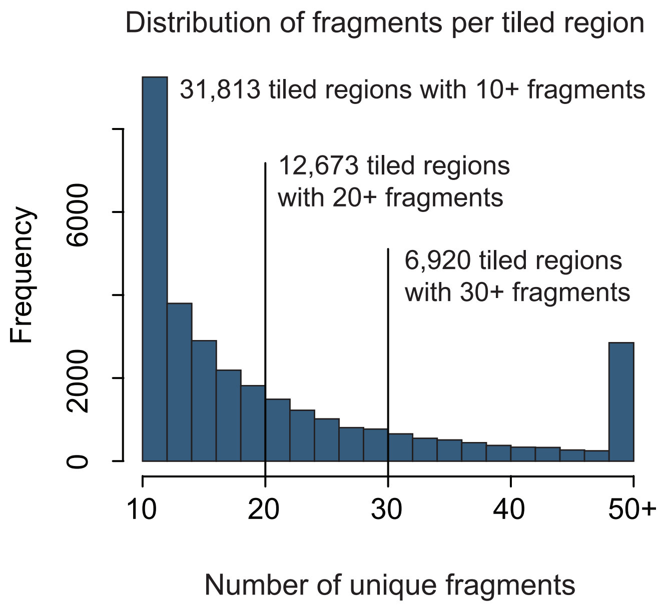

Supplementary Figure 8: Number of unique fragments per tiled region for high-resolution mapping.

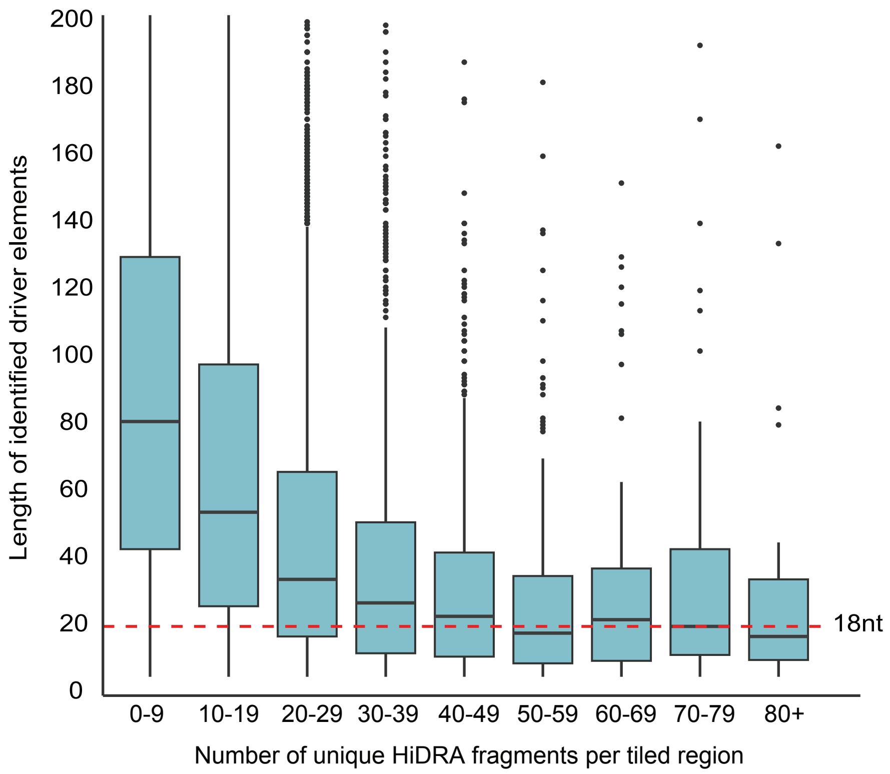

Supplementary Figure 9: Length of high-resolution driver elements depends on coverage. Driver elements identified by SHARPR-RE are smaller in size for tiled regions covered by more fragments. Decrease in driver element size plateaus around 40-50 HiDRA fragments, to reach an expected minimum size of ~18nt.

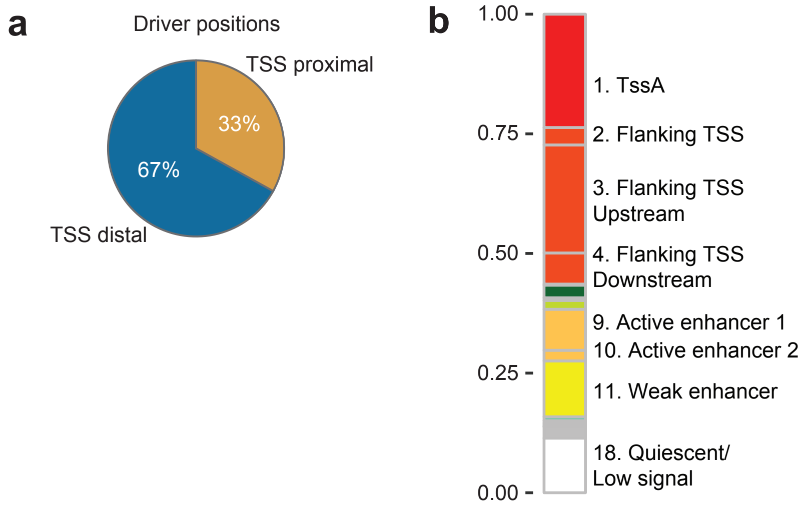

Supplementary Figure 10: Genomic distribution of HiDRA driver elements. (a) The majority of driver elements are distal to annotated transcription start sites (b) Genomic distribution of driver elements reveals that majority of driver elements are found in TSS, TSS-flanking and predicted enhancer regions.

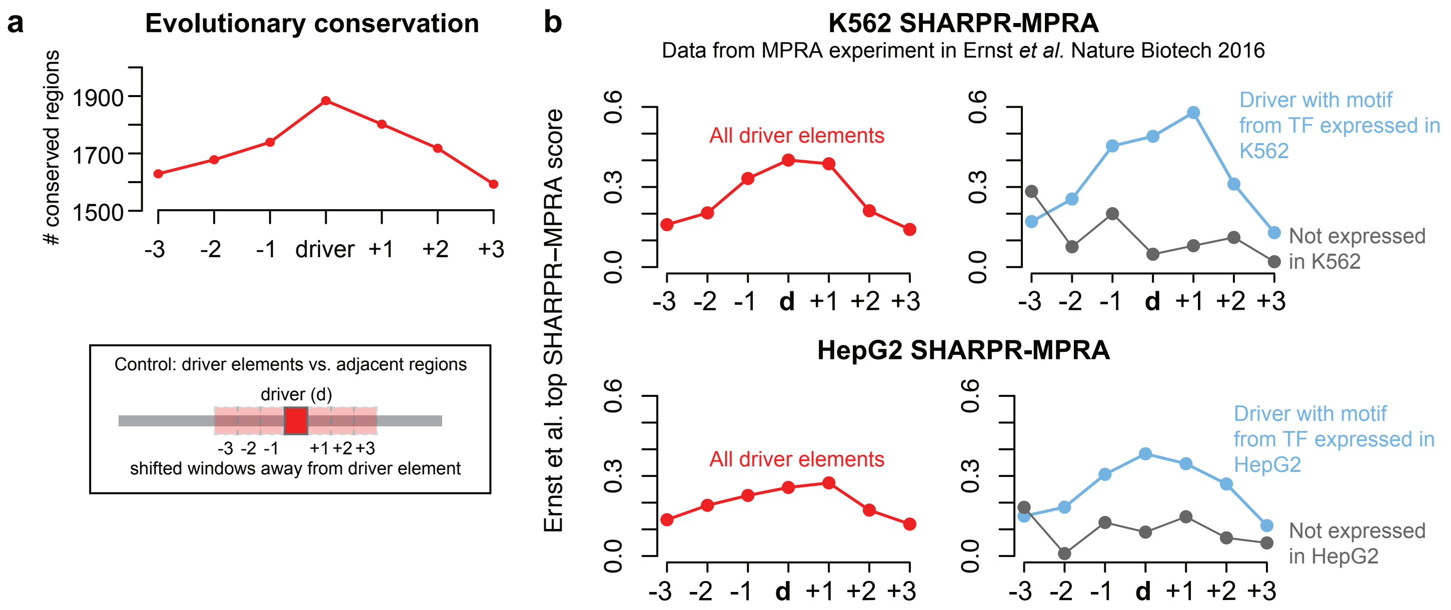

Supplementary Figure 11: Additional functional properties of driver elements. (a) Driver elements have greater evolutionary conservation compared to adjacent regions both upstream and downstream. +/- 1,2,3 values represent control windows of equal size to the driver element shifted upstream (- values) and downstream (+ values) by the length of the driver element. (b) *Left*, Driver elements show greater functional importance scores from independent SHARPR-MPRA experiment in both K562 and HepG2. +/- 1,2,3 values correspond to controls used in panel a. *Right*, functional importance scores for driver elements in K562 and HepG2 driven by drivers containing motifs from TFs expressed in respective cell lines.

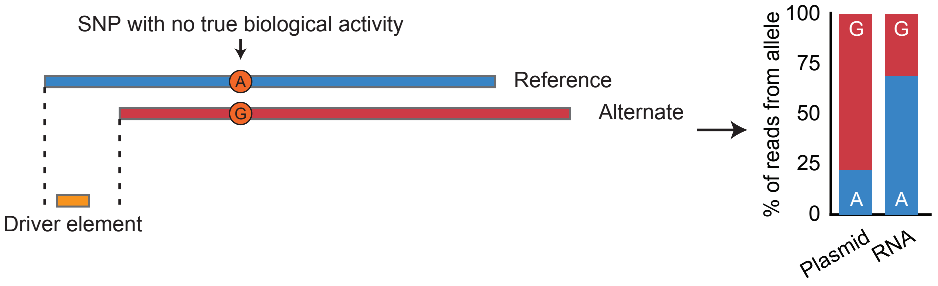

Supplementary Figure 12: Confounding effect of differing fragment positions for allelic activity analyses
As HiDRA relies on random fragmentation of the genome, fragments carrying different alleles at a SNP might have differential activity due to the position of their ends, rather than due to allelic activity. In this hypothetical example, a SNP with no true allelic activity is mistakenly called as active because the fragment containing the reference allele overlaps a driver element not present in the alternate fragment.

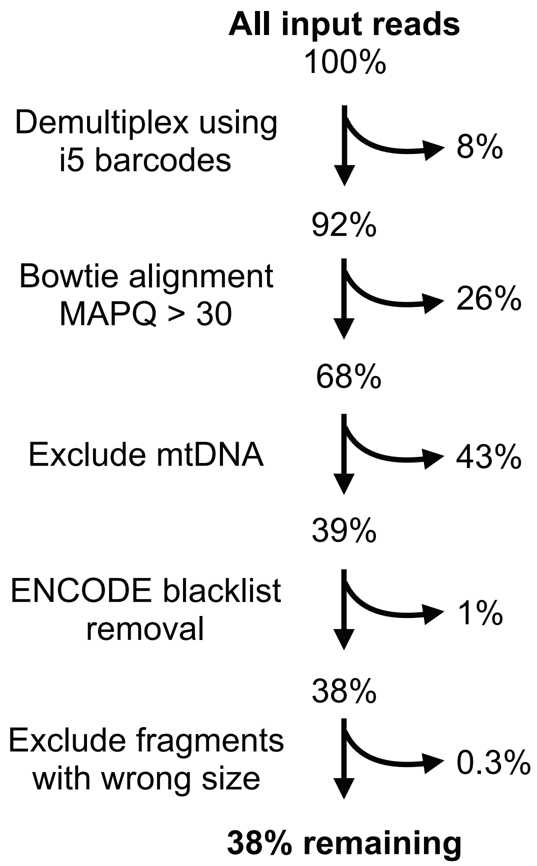

Supplementary Figure. 13: Proportion of reads lost by each processing filter for HiDRA library.

Supplementary Notes

0) Note on HiDRA insert size - we recommend caution before trying very large fragments (e.g. 800nt and above) in HiDRA or STARR-seq. If sequencing on an Illumina machine, fragments of this size do not cluster efficiently and can lead to poor sequencing results. We tried a library with a wide length distribution of 600-1.5kb, and found that large fragments (800nt and above) were very poorly represented in plasmid samples. Surprisingly, some of the strongest "active" regions in RNA output were large fragments (1kb and above) - this is likely an artificial signal due to internal splicing of some large fragments, creating a smaller fragment that is very efficiently sequenced. A more detailed study of this artifact could yield insights into RNA splicing of non-genic regions and the evolution of new genes.

1) We performed 16 ATAC-seq reactions on 50,000 GM12878 cells each. We chose to perform extra ATAC-seq reactions to ensure high library complexity, but performing so many reactions is not necessary if initial cell/tissue amount is an issue.

2) 16 reactions chosen to maintain high library complexity and allow for low cycle number based on slide 67 from https://www.broadinstitute.org/files/shared/illuminavids/SamplePrepSlides.pdf. We did not quantify library complexity with fewer reactions, but if reagents or time are an issue, reducing number of reactions will probably have minimal effect on the library.

3) Mitochondrial fragment depletion was useful, however for future studies we recommend designing a denser set of gRNAs to achieve greater amounts of depletion to save on high-throughput sequencing costs later.

4) In subsequent tests we found that in our hands that the NEBuilder HiFi DNA Assembly enzyme (NEB #e5520) yielded approximately 8-10X more bacterial colonies per reaction using the same primers as described here, in our hands. Based on manufacturer's' literature, primers with longer homology arms (20-25 nt overlaps) should yield even greater efficiency.

5) We recommend using MegaX DH10B T1R cells, or performing extensive tests if changing to a different line of bacteria. In our hands, we experienced substantially lower transfection efficiencies and greater degree of arcing when using other electrocompetent cells (e.g. NEB 10-beta).

6) The most important consideration for HiDRA library preparation is the expected complexity (number of unique fragments). If the complexity is too low, there will be insufficient fragments in most regulatory regions for high-resolution mapping. If library complexity is too high, more cells may need to be transfected for reliable activity readings, and DNA and RNA libraries will need to be sequenced to greater depth. While developing HiDRA, we were able to generate plasmid libraries with over 30-50 million unique fragments (almost an order of magnitude greater than the data presented here), however this would require very large sequencing runs. In our experience, library complexity can be controlled in the (bottleneck) homology-based cloning step. Before proceeding through time-consuming and expensive transfection, RNA collection & RNA sequencing steps, we recommend sequencing the plasmid library (MiSeq or spiking into a larger run) to estimate library complexity and proportion of reads within interesting regions (enhancers, promoters, etc).

7) As the Qiagen Oligotex mRNA kits are fairly expensive, another option is to synthesize biotin-labelled capture probes against the reporter gene transcript and perform streptavidin bead pull-down, as described by Tewhey *et al*. (Cell, 2016), but modifying the probe sequences to match the sgGFP reporter gene on pSTARR-seq_human.

8) The Superscript III RT manual recommends using no more than 500ng of polyA RNA for reverse transcription reactions. As we are only reverse transcribing a single gene and not the entire transcriptome, we reasoned we could use add more polyA RNA per reaction. We selected 2ug of polyA RNA after performing reverse transcription reactions with increasing amounts of RNA followed by reaction cleanup and 6-cycle PCR to quantify yield.

9) If possible we recommend balancing libraries on either a MiSeq or by spiking in on a sequencing run. We have tried Kapa kits, Bioanalyzer and Qubit/Nanodrop, and balancing by MiSeq/sequencing is the best option.

10) We always recommend filtering against the ENCODE blacklist regions, especially for ATAC-seq or ATAC-seq-esque libraries due to the presence of a pseudo-mitochondrial region near the beginning of chr1 that will otherwise substantially affect downstream analyses. Adapter removal is also important if read length is greater than minimum fragment size.

11) The experimental approach we used to sequence the RNA output could conceivably also include false positive results from promoters that have broad, dispersed initiation (i.e. initiation on the STARR-seq plasmid occurs immediately upstream of the inserted fragment, rather than the minimal promoter). In our dataset, promoters that appear to have the ability to promote transcription initiation in a broad upstream region (based on CAGE-seq data from the FANTOM consortium) do not have substantial activity (26 possible cases with FDR<0.05 out of ~66,000 active regions), however this possibility should be considered for future studies using this experimental approach.

Supplementary Methods

SHARPR-RE methodology

A1. Model specification

Basic model

We define a “tiled region” as a continuous region in which each position is covered by at least one HiDRA fragment. Suppose that a tiled region containing P positions is covered by R fragments. The regulatory activity of each fragment j with a length $l_j, j \in \{1, \dots, R\}$ is measured by the ratio $\frac{\#RNA_j}{\#DNA_j}$ between the counts of sequenced RNA and DNA. For a design with multiple replicates, the ratio can be calculated from the average counts of RNA and DNA across the replicates. In this paper, we calculated RNA/DNA ratios for each fragment after normalization of RNA & DNA by DESeq2 with the library split into 100nt bins (100-200nt, 200-300nt, etc). We expect that the ratio for a fragment containing one or more functional driver element site is larger than those not overlapping a driver element. For the downstream analysis, we use the transformed observation M_j^o by taking the log-transformation with base *e* of $\frac{\#RNA_j}{\#DNA_j}$, i.e., $M_j^o = \ln(\frac{\#RNA_j}{\#DNA_j})$. For the HiDRA library described in the Methods section, we observed that the empirical distribution of M_j^o across the whole genome (approximately 4 million fragments after quality control and filtering for minimum expression) is nearly symmetrically centered at zero but with heavy tails that indicate regulatory activity (Supplementary Figure 14).

In HiDRA, the length of a tiled region is generally much larger than the number of fragments ($P \gg R$). The basic idea of SHARPR-RE is to use a shrinkage prior to tackle this large *p* small *n* problem. We first compute a centered variable M_j for each fragment j by subtracting μ_a, the mean of the background signal ($i.e., \ M_j = M_j^o - \mu_a$). The mean of the background signal μ_a is the average signal intensity from fragments not overlapping a driver element. We estimate μ_a by the mean of the observations taken from all tiled regions covered by <5 fragments across the whole chromosome, with the assumption that the majority of these tiled regions do not contain a driver element. More specifically, suppose that there are K tiled regions on a chromosome and each tiled region is covered by R_k fragments each of which has an observation M_{jk}^o, $j \in \{1, \dots, R_k\}$ and $k \in \{1, \dots, K\}$. Thus, we have $\hat{\mu}_a = \sum_{k \in B} \sum_{j=1}^{R_k} M_{jk}^o \,/\, \sum_{k \in B} R_k$, where B is the set of all tiled regions covered by <5 fragments ($B = \{k | R_k < 5\}$).

Within one tiled region, we assume that M_j (we omit the index k whenever the formula only involves a specific tiled region) follows an *i.i.d.* normal distribution with a mean equal to a scaled sum of those regulatory scores A_i that are covered by fragment j, that is,

$$M \sim \mathcal{N}(L^{-1} T A, \Sigma_m), \qquad (1)$$

where $T \in \{0,1\}^{R \times P}$ is an indicator matrix, i.e., $T_{ij} = 1$ if position i, $i \in \{1, \dots, P\}$, is covered by fragment j; otherwise $T_{ij} = 0$, and $L \in \mathbb{R}^{R \times R}$ is a diagonal matrix for scaling each fragment. Note that this specification of T assumes that each position in the tiled region contributes identically to the regulatory activity measurement of the fragments. If, for example, driver elements at the ends of a fragment may contribute less to the regulatory activity, smaller weights can be assigned according to its distance to the middle of the fragment. For the purpose of regularization, we impose an ℓ_2 penalty on A, which is equivalent to a normal prior from the Bayesian perspective. Generalizing SHARPR-MPRA[1] from 5nt to 1nt, the regulatory score A_i at each position i, which is a latent variable, is assigned by a univariate normal prior

$$A_i \sim \mathcal{N}(0, \sigma_a^2), \qquad (2)$$

where σ_a^2 is a hyper-parameter, which is defined by users and is tested for specific values in SHARPR-MPRA [1]. In SHARPR-MPRA, it is assumed that $L_{jj} = l_j$. Because each fragment has the same length in SHARPR-MPRA, we end up with $L = lI$ and $M \sim \mathcal{N}(\frac{TA}{l}, \Sigma_m)$ [1], where I is the identity matrix. In contrast, each fragment has a different length in HiDRA ranging from 150nt to 500nt. In SHARPR-RE, we choose a uniform scale coefficient $L_{jj} = \bar{l}$, where $\bar{l} = \sum_{k=1}^{K} \sum_{j=1}^{R_k} l_{jk} / \sum_{k=1}^{K} R_k$ is the average length of all fragments on the chromosome. Under this modeling of L, the signal of a fragment depends only on the sum of the regulatory scores at all positions that the fragment covers but not on the fragment length. $\Sigma_m \in \mathbb{R}^{R \times R}$ is a covariance matrix with non-zero diagonal elements equal to σ_m^2, which is set to be the sample variance of M_j in SHARPR-MPRA [1]. Thus, the marginal distribution of M after integrating out A from (1) follows

$$M \sim \mathcal{N}(0, L^{-1}T\Sigma_a(L^{-1}T)' + \Sigma_m), \qquad (3)$$

where $\Sigma_a = \begin{pmatrix} \sigma_a^2 & \cdots & 0 \\ \vdots & \ddots & \vdots \\ 0 & \cdots & \sigma_a^2 \end{pmatrix}$ is a diagonal matrix and the prime stands for transpose. Thus, the ridge estimate or the posterior mean of A given the observed M is

$$\hat{A} = \Sigma_a(L^{-1}T)'(L^{-1}T\Sigma_a(L^{-1}T)' + \Sigma_m)^{-1}M. \qquad (4)$$

After some rearrangement to merge Σ_a and Σ_m, we end up with the following equation

$$\hat{A}_\lambda = ((L^{-1}T)'L^{-1}T + \lambda I)^{-1}(L^{-1}T)'M \qquad (5)$$

where $\lambda = \frac{\sigma_m^2}{\sigma_a^2}$ is the penalizing coefficient.

Selection of penalizing coefficient

Instead of letting σ_a^2 and thus the penalizing coefficient λ be defined by users as in Ernst *et al.*[11], we select λ in a data-driven way. This is because the choice of λ substantially affects the estimates and the performance of the following hypothesis testing procedure. This means that λ should be selected carefully. Note that although the formula (4) is essentially the same as the posterior mean in the Bayesian framework used in SHARPR-MPRA, we instead regard (5) as a ridge estimate under the classical framework in SHARPR-RE. In this case, we only assume that (1) is the true model in which A are parameters rather than random variables, and (2) is used for the purpose of regularization. Note that in this case the choice of λ has significant influence on the estimation of A. If λ is too small, the estimates would be unstable, while an overly large λ would bring more bias. A handful of strategies have been proposed to select an optimal and stable λ, including cross-validation[2], the Hoerl-Kennard-Baldwin plug-in method[3-5], and a Markov chain Monte Carlo (MCMC) method[6]. In SHARPR-RE, we select λ by following the strategy proposed by Cule and De Iorio, 2013, which generalizes the idea of Hoerl *et al.* 1975[5] to the large *p* small *n* problem and shows fast and stable estimation in simulation and real data studies. More specifically, we first perform a singular value decomposition (SVD) for $L^{-1}T$:

$$L^{-1}T = UDV',$$

where D is a diagonal matrix with t non-zero diagonal elements d_{jj}, and $t \leq \min(P, R)$. We select $r^* \in \{1, \ldots, t\}$, so that

$$r^* = \operatorname{argmin}_r \, r - \sum_{j=1}^{t} \frac{{d_{jj}}^4}{({d_{jj}}^2 + \lambda_r)^2},$$

where we have

$$\lambda_r = \frac{r\hat{\sigma}_r^2}{\hat{\eta}_r'\hat{\eta}_r},$$

$$\hat{\eta} = D^{-2}V'(L^{-1}T)'M,$$

and $\hat{\sigma}_r^2 = \frac{(M-L^{-1}TV_r\hat{\eta}_r)'(M-L^{-1}TV_r\hat{\eta}_r)}{R-r}$,

where $\hat{\eta}_r$ is an r-vector of the first r elements in $\hat{\eta}$, and V_r is the first r column of V.

Given r^*, we choose λ as $\lambda_{r^*} = \frac{r^*\hat{\sigma}_{r^*}^2}{\hat{\eta}_{r^*}'\hat{\eta}_{r^*}}$, and the estimate of A in SHARPR-RE is

$$\hat{A}_{\lambda_{r^*}} = ((L^{-1}T)'L^{-1}T + \lambda_{r^*}I)^{-1}(L^{-1}T)'M = H_{\lambda_{r^*}}M, \quad (6)$$

where $H_{\lambda_{r^*}} = ((L^{-1}T)'L^{-1}T + \lambda_{r^*}I)^{-1}(L^{-1}T)'$ is the hat matrix. For HiDRA datasets, it is often the case that the number of fragments R is much smaller than the length of a tiled region P. To make the computation more efficient, we apply SVD to the hat matrix to avoid the inversion of a large-scale matrix, so that we have

$$\begin{aligned} H &= ((L^{-1}T)'L^{-1}T + \lambda_{r^*}I)^{-1}(L^{-1}T)' \\ &= (VD'U'UDV' + \lambda_{r^*}VV')^{-1}VD'U' \\ &= V(D'U'UD + \lambda_{r^*}I)^{-1}V'VD'U' \\ &= V(D'U'UD + \lambda_{r^*}I)^{-1}D'U', \end{aligned}$$

in which the computation of UD is dramatically faster as D has at most R non-zero diagonal elements. In the analysis of the example HiDRA library, we observed that this algorithm of selecting λ_{r^*} produced stable estimates of the regulatory scores. We also noticed that the algorithm would produce an overly small λ_{r^*} if two or more fragments in a tiled region are mapped to almost the same position (the difference is only a couple of nucleotides) and have large opposite values of $\ln(\frac{\#RNA_j}{\#DNA_j})$. This phenomenon may suggest a potential data problem. Note that this algorithm estimates a unique λ_{r^*} for each tiled region, and thus the estimated regulatory scores cannot be compared directly across tiled regions. If the comparison across regions is the major concern (e.g., using the estimated regulatory scores as a training set in deep learning such as convolutional neural networks (CNN) for other downstream analysis), studentized estimates $Z_{\lambda i}$ can be used (described in the next section).

Accuracy of estimation

To measure the accuracy of the estimates, we compute the pointwise mean square error (MSE) of $\hat{A}_\lambda$. As we assume that (1) is the true model, $\hat{A}_\lambda$ is a biased estimate of A if $A \neq 0$, and the MSE of $\hat{A}_\lambda$ should take into account both variance and bias. That is, we are interested in finding not only $Var(\hat{A})$ but $E(\hat{A}_\lambda - A)^2$ as well. Note that the MSE can be decomposed into

$$MSE(\hat{A}_\lambda) = Var(\hat{A}_\lambda) + Bias(\hat{A}_\lambda)^2,$$

where $Bias(\hat{A}_\lambda) = E(\hat{A}_\lambda) - A$ measures the bias between the true value of A and the mean of $\hat{A}_\lambda$. The bias term is given by

$$Bias(\hat{A}_\lambda)^2 = (E(\hat{A}_\lambda) - A)(E(\hat{A}_\lambda) - A)'$$

$$= (((L^{-1}T)'L^{-1}T + \lambda I)^{-1}(L^{-1}T)'L^{-1}T - I)AA'(((L^{-1}T)'L^{-1}T + \lambda I)^{-1}(L^{-1}T)'L^{-1}T - I)'$$

$$= (W_\lambda - I)AA'(W_\lambda - I)',$$

where $W_\lambda = H_\lambda L^{-1}T$.

The variance $Var(\hat{A}_\lambda)$ can be shown as

$$Var(\hat{A}_\lambda) = Var(((L^{-1}T)'L^{-1}T + \lambda I)^{-1}(L^{-1}T)'M)$$

$$= ((L^{-1}T)'L^{-1}T + \lambda I)^{-1}(L^{-1}T)'Var(M)(((L^{-1}T)'L^{-1}T + \lambda I)^{-1}(L^{-1}T)')'$$

$$= \sigma_m^2((L^{-1}T)'L^{-1}T + \lambda I)^{-1}(L^{-1}T)'L^{-1}T(((L^{-1}T)'L^{-1}T + \lambda I)^{-1})'$$

$$= \sigma_m^2 H_\lambda H_\lambda{}'.$$

The true value of σ_m^2 is unknown, but can be estimated from the residuals

$$\hat{\sigma}_m^2 = \frac{(M - L^{-1}T\hat{A}_\lambda)'(M - L^{-1}T\hat{A}_\lambda)}{\text{df}},$$

where $\text{df} = R - 2tr(H_\lambda) + tr(H_\lambda H_\lambda{}')$ is the residual degrees of freedom[7] and $tr()$ stands for the trace. Plugging in the ridge estimate (5) to A and the sample estimate $\hat{\sigma}_m^2$ to σ_m^2, the estimated MSE is

$$\widehat{MSE}(\hat{A}_\lambda) = \widehat{Var}(\hat{A}_\lambda) + \widehat{Bias}(\hat{A}_\lambda)^2 \quad (7)$$
$$= \hat{\sigma}_m^2 H_\lambda H_\lambda{}' + (W_\lambda - I)\hat{A}_\lambda \hat{A}_\lambda{}'(W_\lambda - I)',$$

Pointwise confidence intervals (CIs) can be calculated from $\widehat{Var}(\hat{A}_\lambda)$, e.g., $95\%\text{CI} \approx \hat{A}_\lambda \pm 1.96 \times \sqrt{\widehat{Var}(\hat{A}_\lambda)}$. Note that the bias term $Bias(\hat{A}_\lambda)$ is non-zero if A or λ is non-zero. Therefore, it is not straightforward to interpret the CIs obtained from $Var(\hat{A}_\lambda)$. Instead, the following adjusted 95%CI

$$\text{CI}_{\text{adj}} = \hat{A}_\lambda - \widehat{Bias}(\hat{A}_\lambda) \pm 1.96 \times \sqrt{\widehat{Var}(\hat{A}_\lambda)}$$

is proposed [8], which adjusts for the bias. One problem of the adjusted CI is that the true bias is unknown and its estimate $\widehat{Bias}(\hat{A}_\lambda)$ might not be accurate.

A2. Identifying high-resolution driver elements

Regional FWER controlling procedure

Given the estimated regulatory scores $\hat{A}_\lambda$ for each nucleotide within a specific tiled region, we then aim at finding a regional threshold to declare significant regulatory regions, which we term as high-resolution "driver" elements at which an active motif is located. More specifically, we need to make the inference for each nucleotide i by testing the following hypothesis,

$$H_0: A_i = 0 \; vs. H_a: A_i > 0.$$

For this hypothesis testing, we focus only on finding activating regulatory elements but not repressive ones; however, generalization to a two-sided test is straightforward. For a specific tiled region containing P positions, we want to find a cutoff c_i so that the family-wise error rate

(FWER) α is bounded below a given value (e.g., 0.05). The value of α can be set differently among different tiled regions. This amounts to a multiple testing problem of performing P one-sided tests of the estimated regulatory scores $\hat{A}_\lambda = (\hat{A}_{\lambda 1}, \ldots, \hat{A}_{\lambda P})' = 0$ simultaneously. One way can be computing a p-value for each $\hat{A}_{\lambda i}$ and using the simple Bonferroni correction to obtain a local significance level $\alpha_i = \frac{\alpha}{P}$ from which c_i can be computed. This approach would be overly conservative as $\hat{A}_{\lambda i}$ was not independent of each other in this case. A more accurate cutoff should take into account the correlation structure of the estimated regulatory scores. On the other hand, performing a permutation test for each tiled region would be too time consuming for a library comprising the whole genome albeit more accurate. Following the strategy described by [9,10], we thus propose a fast multiple testing procedure based on Gaussian copula to find region-specific cutoffs for controlling FWER α. Note that under the null hypothesis $A_i = 0$, the bias term in (7) disappears. We use the studentized estimate as the test statistics

$$Z_{\lambda i} = \frac{\hat{A}_{\lambda i}}{\sqrt{\widehat{Var}(\hat{A}_{\lambda i})}} = \frac{\hat{A}_{\lambda i}}{\hat{\sigma}_m \sqrt{\operatorname{diag}(H_\lambda H_\lambda')_i}},$$

where $\operatorname{diag}()_i$ stands for the ith element in the vector of the diagonal elements of a matrix. It has been shown that under the null hypothesis, $Z_{\lambda i}$ follows a Student *t*-distribution and can be approximated by a standard normal distribution under a large sample size [11,12]. Cule *et al.* 2011 find through simulation studies that the type I error rate and the statistical power using the normality approximation are comparable to those from permutation tests for a wide range of λ. This observation motivates us to assume that under the null hypothesis, Z_λ approximately follows a multivariate normal distribution

$$Z_\lambda = \hat{\sigma}_m^{-1} (H_\lambda H_\lambda' \odot I)^{-\frac{1}{2}} \hat{A}_\lambda = S\hat{A}_\lambda \sim \mathcal{N}(0, S\widehat{Var}(\hat{A}_\lambda)S), \tag{8}$$

where $\odot$ is the Hadamard product and $S = \hat{\sigma}_m^{-1}(H_\lambda H_\lambda' \odot I)^{-\frac{1}{2}}$. In the simulation studies provided in the next section, we investigate the empirical FWER based on this multivariate normal approximation under small sample size and high-dimensional cases. Denote by $F_i(x_i)$ the marginal cumulative density function (CDF) of Z_λ, which is continuous. According to Sklar's theorem[13], there exists a unique copula $\mathcal{C}: [0,1]^P \to [0,1]$ such that $\forall\, (x_1, \ldots, x_P)' \in \mathbb{R}^P: F(x_1, \ldots, x_P) = \mathcal{C}\big(F_1(x_1), \ldots, F_P(x_P)\big)$, where $F(x_1, \ldots, x_P)$ is the joint CDF. Hence, for the one-sided test we have

$$\begin{aligned} \alpha = \mathrm{FWER} &= \mathbb{P}_{H_0}\left(\bigcup_{i=1}^{P} Z_{\lambda i} > c_i\right) \\ &= 1 - \mathbb{P}_{H_0}\left(\bigcap_{i=1}^{P} Z_{\lambda i} \le c_i\right) = 1 - \mathbb{P}_{H_0}(Z_{\lambda 1} \le c_1, \ldots, Z_{\lambda P} \le c_P) \\ &= 1 - \mathcal{C}\big(F_1(c_1), \ldots, F_P(c_P)\big) \end{aligned}$$

Under the multivariate normality approximation of (8), we have

$$\alpha = 1 - \mathcal{C}\big(F_1(c_1), \ldots, F_P(c_P)\big) = 1 - \mathcal{C}_{S\widehat{Var}(\hat{A}_\lambda)S}\big(\Phi_1(c_1), \ldots, \Phi_P(c_P)\big), \tag{9}$$

where $\mathcal{C}_{S\widehat{Var}(\hat{A}_\lambda)S}(u_1, \ldots, u_P)$ is a Gaussian copula with a correlation parameter matrix of $S\widehat{Var}(\hat{A}_\lambda)S$, and $\Phi(\mathrm{c})$ is the CDF of a standard normal distribution. Given a specific value of α, there are infinite many solutions $(u_1, \ldots, u_P) = \mathcal{C}^{-1}_{S\widehat{Var}(\hat{A}_\lambda)S}(1-\alpha)$. However, if we treat every

position as equally important and pursue a single-step common-quantile cutoff (Dudoit and van der Laan, 2008, Chapter 4) c, i.e., $c_1 = \cdots = c_P = c$, we can find a unique solution

$$u^* = \mathcal{C}^{-1}_{S\widehat{Var}(\hat{A}_\lambda)S}(1-\alpha), at\ u_1 = \cdots = u_P = u^*,$$

and

$$c^* = \Phi^{-1}(u^*).$$

So, we reject H_0 for the positions in $\mathcal{H} = \{i \in (1, \dots, P): Z_{\lambda i} > c^*\}$. The common-quantile cutoff c^* can be calculated, for example, by the function *qmvnorm* in the R package *mvtnorm*[14]. The similar idea can also be used to obtain adjusted p-values for controlling regional FWER as shown in Conneely and Boehnke, 2007[15]. In real data analysis, the estimated covariance matrix $\widehat{Var}(\hat{A}_\lambda)$ is often degenerated and the estimates of adjacent positions are completely correlated when $P > R$. Therefore, we trim the number of the estimates by selecting one position from each group in which the estimates for the positions are completely correlated. This also dramatically reduces the computational intensity for finding the solution to (9). After identifying the driver elements, we can further attempt to pinpoint the location of the most possible occurrence of a 20nt "core" driver element (see section A4 below for rationale for choosing ~20nt as the estimated "core" region). We predict the center position i_m of a 20nt core driver element by the highest regulatory scores over its 20nt flanking region, i.e.,

$$i_m = \text{argmax}_{i \in \mathcal{H}} \frac{\sum_{n=(i-9)\vee 1}^{(i+10)\wedge P} \hat{A}_{\lambda n}}{(i+10)\wedge P - (i-9)\vee 1 + 1}.$$

Supplementary Figure 16 gives an illustration of the significant regulatory region and the predicted motif region. In this example, the true motif is located at position 400-420nt and is covered by an identified significant driver element by SHARPR-RE (highlighted in red). The predicted core driver region (highlighted in purple) further pinpoints the location of the motif at ~400nt.

Global FDR controlling procedure

The above regional procedure calls significant driver elements for a specific tiled region. If we want to identify driver elements across an entire genome, it may be preferable to control the global false discovery rate (FDR). We thus propose a global multiple testing correction procedure for this purpose by taking into account the p-values observed from the whole genome. We first calculate the pointwise p-values for all positions in each tiled region across the genome based on the *t*-distribution

$$Z_{\lambda i} = \frac{\hat{A}_{\lambda i}}{\sqrt{\widehat{Var}(\hat{A}_{\lambda i})}} \sim t_{R - tr(H_\lambda)},$$

where $R - tr(H_\lambda)$ is the sample size minus the effective degrees of freedom. As mentioned in the local controlling procedure, we select one position from a consecutive region in which the estimates for these positions are completely correlated. Then, we apply the Benjamini-Hochberg procedure to the pointwise p-values to control the global FDR at level α. As p-values from different tiled regions are independent, the p-values across the genome can be regarded being dependent in finite blocks if the size of the largest tiled region is limited. More specifically, we assume that the ratio between max(R_k) and the total number of fragments $\sum_{k=1}^{K} R_k$ goes to zero as $\sum_{k=1}^{K} R_k \to \infty$ (i.e., $\frac{\max(R_k)}{\sum_{k=1}^{K} R_k} \to 0\ as\ \sum_{k=1}^{K} R_k \to \infty$, and R_k is used instead of P_k because the

number of tests in a tiled region is related to R_k when $P_k \gg R_k$). Thus, under this assumption, which is biologically reasonable, the estimate of FDR is consistent[16,17].

A3. Evaluation of empirical statistical power and FWER

Simulation settings

We assessed the performance of the proposed SHARPR-RE algorithm in terms of empirical statistical power estimated from our simulation studies. To mimic the current version of the HiDRA library, we randomly generated a number R of fragments (R between 25-100) in a tiled region with $P = 1kb$. The length of each fragment was sampled from a uniform distribution $l_j \sim U(175,450),\ j \in \{1, \dots, R\}$. We randomly selected a 20nt driver element from a 400nt window in the middle of the tiled region. For any fragment that covers the driver element, we generated its signal from a normal distribution $\mathcal{N}(\mu_{driver} = S_m, \sigma_{driver} = 0.1)$, where S_m is the true signal varying across different simulation scenarios. For the rest of the fragments, we generated signals from a normal distribution $\mathcal{N}(\mu_{noise} = 0, \sigma_{noise} = 1)$. We defined the signal-to-noise-ratio (SNR) as $\mathrm{SNR} = \frac{S_m}{\sigma_{noise}}$. We examined the empirical FWER and empirical statistical power under different SNR and numbers of fragments. Under each simulation setting, we generated 500 replicates to obtain the estimates of the empirical FWER and statistical power.

Evaluation of empirical type I error rate

Our results in Table 1 show that generally the empirical regional FWER was controlled at ~5%, which was the theoretical FWER, when the number of fragments was above 50. We observed mild inflation of the empirical FWER especially in the case of small sample size (e.g., 25), but the inflation diminished with the sample size increasing in most situations. This inflation can be due to the discrepancy between the true null distribution of the statistics and the asymptotic multivariate normal distribution at the tails as shown in [18]. This indicates that the error introduced by the multivariate normality approximation should be taken into account when the sample size is overly small (for example, by using a similar scaling procedure as proposed in [18] or by setting a more stringent cutoff for a tiled region covered by a small number of fragments).

Evaluation of empirical statistical power

Next, we examined the statistical power for pinpointing a driver element under the condition of $\alpha = 5\%$, i.e., the FWER <5%. In this investigation, a true positive is counted if an identified driver element or a predicted 20bp functional motif region overlapping the true driver element region. The results in Supplementary Figure 17 show that the statistical power for both regions consistently increases with respect to the number of fragments and the SNR. If there are 100 fragments in a tiled region, SHARPR-RE can achieve more than 80% power under SNR=1. When the number of fragments is small (e.g., 25), SNR>1.5 is needed to achieve a power of 80%. Higher SNR requires that the biological experiments have higher precision and sensitivity, so that significantly more RNAs can be sequenced when the DNA region covers a true driver element.

A4. Analysis of an HiDRA library

We applied SHARPR-RE to an HiDRA library prepared from the GM12878 lymphoblastoid cell line. The library contains 3,896,416 fragments after quality control, with the length of fragments ranging from 100-600nt (99% of fragments between 168-473nt). We first identified 645,936 tiled regions that were covered by at least two fragments across the whole genome, among which 28,092 regions were covered by more than 10 fragments. The distribution of the signals (ln(#RNA/#DNA)) of these fragments are almost symmetrically centered at zero with heavy tails (Supplementary Figure 14). The average and the variance of the signals are constant across

the length of HiDRA fragments after normalization (Materials and Methods, Supplementary Figure 15).

We estimated the regulatory scores for the 22 chromosomes separately and called driver elements based on a cutoff controlling regional FWER<0.05 for the positions in each tiled region. We found that the tiled regions covered by larger numbers of HiDRA fragments were more likely to have a driver element called, which is likely a combination of greater statistical power and enrichment for regions more likely to contain drivers.

As shown in Figure 5C, most driver elements are found within active TSS, TSS Flanking Upstream and active enhancer chromatin states. The median size of driver elements identified from the tiled regions covered by >10 fragments was 52nt after filtering to remove drivers smaller than 5nt. The average size of drivers decreased with an increase in number of fragments in a tiled region, suggesting that more complex libraries with greater numbers of unique fragments should be able to detect shorter driver elements (Supplementary Figure 9). The average size of a driver element converges to ~18nt after the depth of unique HiDRA fragment coverage reaches 50 fragments/kb (Supplementary Figure 9).

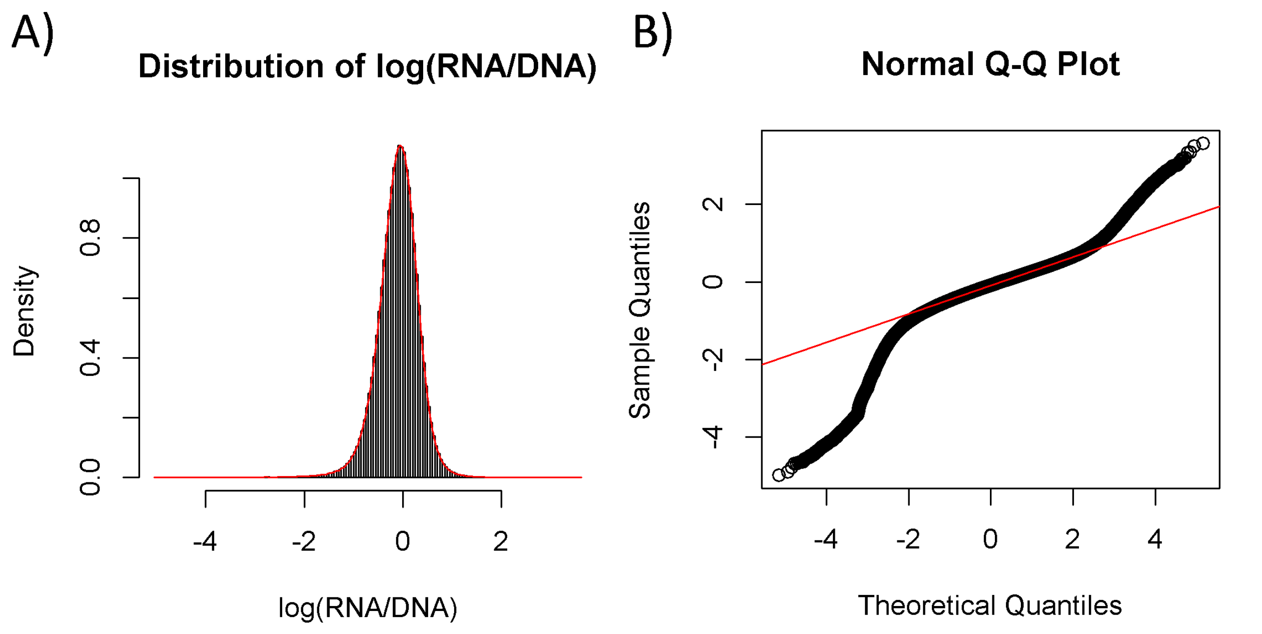

Supplementary Figure 14: A histogram with a density curve of ln(#RNA/#DNA) of the fragments from the library described in the method section. The distribution of ln(#RNA/#DNA) is closer to a normal distribution. The exclusion criteria for the fragments are length<100 or length>600. The Q-Q plot suggests that this is a heavy-tailed distribution.

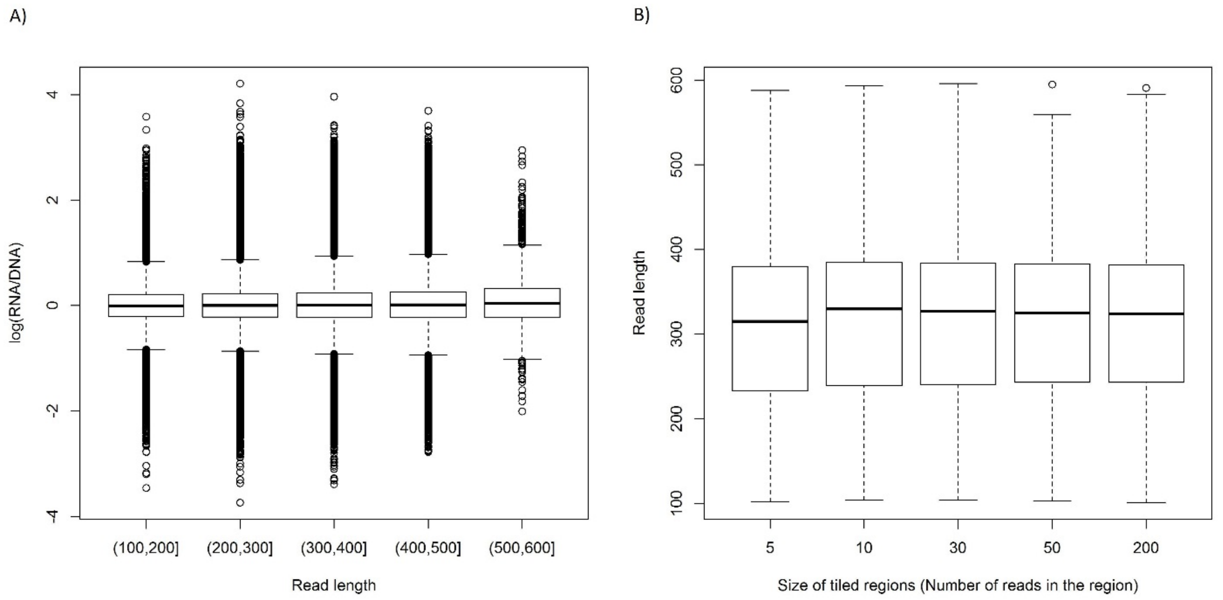

Supplementary Figure 15: Relationship between ln(#RNA/#DNA), fragment length and size of tiled regions. A): the distribution of ln(#RNA/#DNA) after normalization with respect to fragment

length. In the plot, the fragment length is categorized into five groups. B): the distribution of fragment length with respect to the size of the tiled region in which the fragment is located. The size of tiled regions is defined by the number of fragments in the tiled region. The plots are based on the library described in the main text in which the fragments are ranged between 100-600nt (with 99% between 168-473nt)

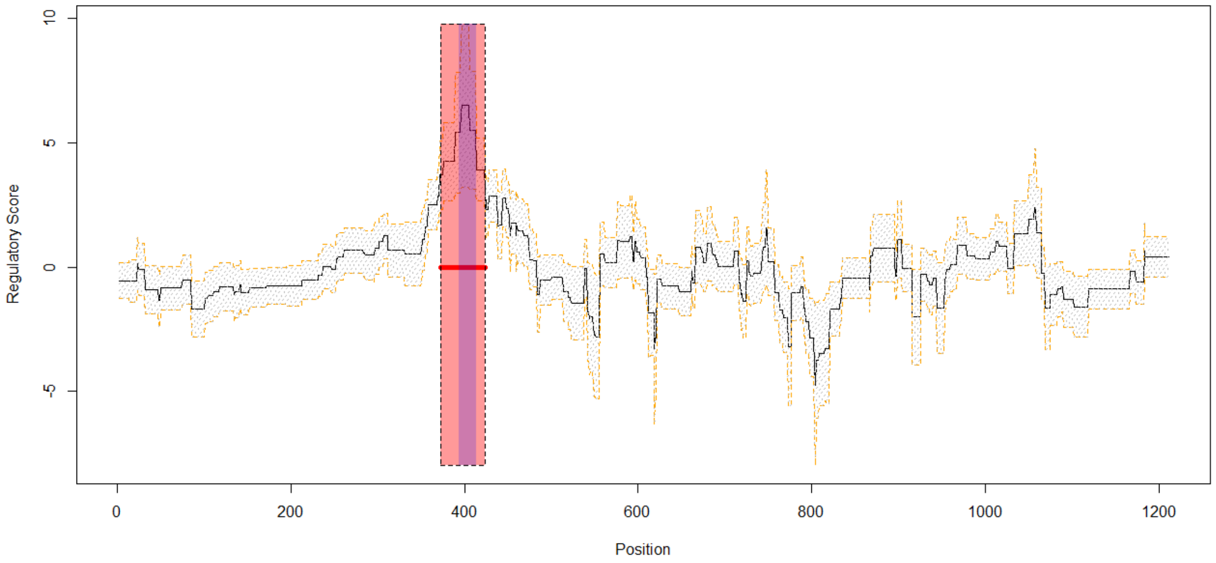

Supplementary Figure 16: An example of estimated regulatory scores from a simulated tile region of 1200nt. The data is generated within a 1200nt tile region with 50 unique HiDRA fragments ranging from 175nt to 450nt. The significant regulatory region (FWER<5%) is highlighted in red. The predicted motif region is highlighted in purple. The yellow dashed lines are the estimated scores ± MSE.

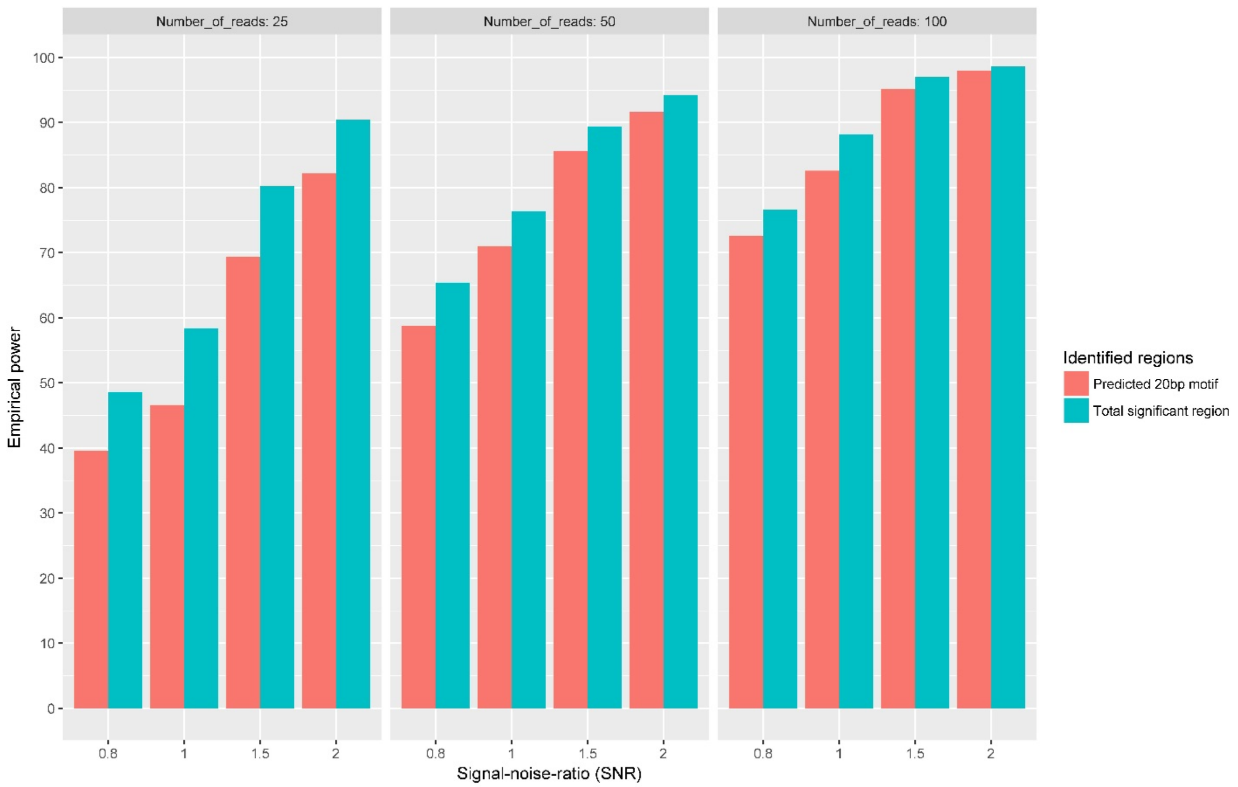

Supplementary Figure 17: Empirical statistical power according to different numbers of fragments and different SNR. Y-axis: empirical power (%). X-axis: Signal-to-noise-ratio defined by $SNR = \frac{S_m}{\sigma_{noise}}$. The red bars are the empirical power for the predicted 20nt core driver element. The blue bars are the empirical power for the identified drivers with a significant regulatory score based on a regional FWER=5%.

Supplementary Tables for SHARPR-RE

Supplementary Table 1: Empirical FWER for the proposed local multiple testing procedure. The empirical FWER was calculated from 500 replicates under each setting. The theoretical FWER α is 5%. We examined the empirical FWER with respect to a max tiled region length (between 900nt and 1500nt) and the number of fragments in the tiled region.

Number of fragments	Max length of a tiled region			
	900	1100	1300	1500
**25**	7.6%	9.0%	7.0%	7.6%
**50**	7.2%	6.0%	4.8%	4.0%
**75**	7.0%	5.2%	5.8%	6.4%
**100**	6.8%	5.0%	6.0%	6.7%
**125**	6.4%	5.4%	6.4%	5.0%

Supplementary References

1. Ernst, J. *et al.* Genome-scale high-resolution mapping of activating and repressive nucleotides in regulatory regions. *Nat Biotechnol* **34,** 1180–1190 (2016).
2. Golub, G. H., Heath, M. & Wahba, G. Generalized Cross-Validation as a Method for Choosing a Good Ridge Parameter. *Technometrics* **21,** 215 (1979).
3. Cule, E. & De Iorio, M. Ridge regression in prediction problems: automatic choice of the ridge parameter. *Genet. Epidemiol.* **37,** 704–714 (2013).
4. HOERL, A. E. & KENNARD, R. W. Ridge Regression - Biased Estimation for Nonorthogonal Problems. *Technometrics* **12,** 55–& (1970).
5. HOERL, A. E., KENNARD, R. W. & BALDWIN, K. F. Ridge Regression - Some Simulations. *Communications in Statistics* **4,** 105–123 (1975).
6. Denison, D. G. T. *Bayesian Methods for Nonlinear Classification and Regression*. (John Wiley & Sons, 2002).
7. Hastie, T., Tibshirani, R. & Friedman, J. *The Elements of Statistical Learning*. (Springer Science & Business Media, 2013).
8. De Brabanter, K., De Brabanter, J., Suykens, J. A. K. & De Moor, B. Approximate confidence and prediction intervals for least squares support vector regression. *IEEE Trans Neural Netw* **22,** 110–120 (2011).
9. Dickhaus, T. & Gierl, J. Simultaneous test procedures in terms of p-value copulae. *Economic Risk* (2012). Available at: http://sfb649.wiwi.hu-berlin.de/papers/pdf/SFB649DP2012-049.pdf. (Accessed: 11 October 2012)
10. Stange, J., Bodnar, T. & Dickhaus, T. Uncertainty quantification for the family-wise error rate in multivariate copula models. *Asta-Advances in Statistical Analysis* **99,** 281–310 (2015).
11. Cule, E., Vineis, P. & De Iorio, M. Significance testing in ridge regression for genetic data. *BMC Bioinformatics* **12,** 372 (2011).
12. Halawa, A. M. & Bassiouni, El, M. Y. Tests of regression coefficients under ridge regression models. *Journal of Statistical Computation and Simulation* **65,** 341–356 (2000).
13. Nelsen, R. B. *An Introduction to Copulas*. (Springer Science & Business Media, 2013).
14. Genz, A. & Bretz, F. Comparison of methods for the computation of multivariate t probabilities. *J. Comput. Graph. Stat* 950–971 (2002).
15. Conneely, K. N. & Boehnke, M. So many correlated tests, so little time! Rapid adjustment of P values for multiple correlated tests. *American Journal of Human Genetics* **81,** 1158–1168 (2007).
16. Schwartzman, A. & Lin, X. The effect of correlation in false discovery rate estimation. *Biometrika* **98,** 199–214 (2011).
17. Storey, J. D., Taylor, J. E. & Siegmund, D. Strong control, conservative point estimation and simultaneous conservative consistency of false discovery rates: a unified approach. *Journal of the Royal Statistical Society Series B-Statistical Methodology* **66,** 187–205 (2004).
18. Han, B., Kang, H. M. & Eskin, E. Rapid and Accurate Multiple Testing Correction and Power Estimation for Millions of Correlated Markers. *PLoS Genet* **5,** (2009).
