# Supplementary material for: High-resolution genome-wide functional dissection of transcriptional regulatory regions and nucleotides in human
Source: Nat Commun. 2018 Dec 19;9:5380. doi: 10.1038/s41467-018-07746-1 (PMC6300699; doi:10.1038/s41467-018-07746-1)
Supplement: Supplementary file 3 — Description of Additional Supplementary Files [file 41467_2018_7746_MOESM3_ESM.pdf]

Description of Additional Supplementary Files

Supplementary Dataset #1:

List of 66,254 active regions discovered in GM12878 by HiDRA (BED file, hg19)

Supplementary Dataset #2:

List of 31,813 tiled regions used for high-resolution mapping by SHARPR-RE (BED file, hg19)

Supplementary Dataset #3:

List of 12,946 high-resolution driver elements discovered in GM12878 (BED file, hg19)

Supplementary Dataset #4:

List of 880 SNPs with allele-specific activity (raw p-value < 0.05) (text file table of SNP and unadjusted p-value)
