# Supplementary material for: High-resolution genome-wide functional dissection of transcriptional regulatory regions and nucleotides in human
Source: Nat Commun. 2018 Dec 19;9:5380. doi: 10.1038/s41467-018-07746-1 (PMC6300699; doi:10.1038/s41467-018-07746-1)
Supplement: Supplementary file 2 — Peer Review File [file 41467_2018_7746_MOESM2_ESM.pdf]

Reviewer #1 (Remarks to the Author):

In this work, Wang and colleagues developed a new high-throughput reporter strategy to assess enhancer activity. The assay, named HiDRA, combined the ATAC protocol, to enrich for accessible chromatin regions, with the STARR-seq reporter assay. HiDRA thus allow a straightforward and cost-effective approach to assess enhancer activity from putative regulatory regions. The authors demonstrated the efficiency of the assays and developed an algorithm to pinpoint the key regulatory regions ("driver" elements). They also show how the assay could be implemented to assess the effect of regulatory variants.

The HiDRA assay has the potential to easy the study of regulatory regions and to be implemented by researchers interested in gene regulation. Overall the study is well conducted and comprehensive analyses were performed. However, I think several issues should be addressed before the manuscript could be accepted for publication, as detailed below.

Specific remarks

1. The authors claim that they obtained a high level of coverage within the ATAC accessible regions. However, they provided the maximum of coverage (370 fragments per region), which is not really informative (abstract and lines 48 and 81); more than half of the fragments are, actually, represented by less than 10 fragments (line 82). Instead, the authors should provide the average of fragments per region). It will be also important to provide a supplemental figure sowing the coverage distribution among the ATAC peaks.

2. The values provided at the end of the introduction ("~95,000 active fragments clustering in ~65,000 regions showing significant regulatory function"; "We discover ~13,000 high resolution driver elements") are somehow confusing because the different terms are not yet defined. These paragraph should be re-writing in a more clear way.

3. From the results provided by the authors it is difficult to assess how well the ATAC peaks are represented in the HiDRA library. Figure 1d shows a screenshot comparing published ATAC-seq with the HiDRA library, but this is not sufficient. The authors should provide a correlation plot between the two datasets in order to pinpoint (or exclude) potential bias.

4. One potential concern of the HiDRA assay is that the results might be biased by the relative accessibility of each region, which does not necessarily correlate with the strength of enhancer activity. For instances, a highly accessible region will be overrepresented in the library and therefore

provide a more reliable result than a less accessible region. One way to assess this potential bias will be to compute the % of active regions in function of chromatin accessibility (obtained from the ATAC-seq signal).

5. As the authors shows in figure 1b, the fragment size of the HiDRA library display a bi-modal distribution (as expected). It will be of potential interest to assess the relative enhancer activity between the small and large fragments. Are active fragments preferentially found within the smaller or larger sizes? For a given driver element, is the length of the fragment somehow affecting the enhancer strength?

6. Given the recent finding that many core promoters display enhancer activity in high-throughput reporter assays (e.g. Nat Genet 49(7): 1073-1081; Nat Methods 15(2): 141-149; Nature 518(7540): 556-559; Genome Res 26(8): 1023-1033), it will be interesting to precisely know what percentage of the regions overlapping TSS of coding genes actually display enhancer activity (instead of using the chromatin state definitions of promoters and TSS-flanking regions, which are ambiguous) and how this percentage compare with the TSS-distal regions.

7. Their findings that some active elements originate from heterochromatin-associated states should be discuss in the light of two recent whole genome reporter assays (Nat Methods 15(2): 141-149; Genome Biol 18(1): 219) reporting similar findings.

8. Related to the above issue, it is surprising that active regions are detected within heterochromatin regions as these are not expected to contain ATAC peaks and therefore should not be present in the HiDRA library. Could be possible that these "active regions" come from background regions with low coverage and therefore less accurate calling? In any case this issue should be discussed.

9. The identification of driver elements is one of the key aspects of the HiDRA assay. However, the functional relevance of these driver elements should be tested by classical reporter assays where the 'driver" region is either tested alone or deleted from the whole enhancer region.

10. The example of the SNP rs2382817 (Figure 6) is awkward. Indeed, the risk allele (A) is associated with increased enhancer activity, but lower expression of the associated gene (TMBIM1). Because this SNP is present in the GM12878 cell line, the authors could directly assess the allele-specific expression of the gene. In any case, this apparent contradiction should be explained.

11. The authors should provide the list of the 880 allelic regions along with the relative allele activity as a supplementary dataset.

12. The list of active fragments and driver elements should also be provided as a supplementary dataset (in addition to the UCSC link).

13. In the Methods section, the size selections performed by gel excision and AMPure beads during library preparation should be indicated.

14. During the library construction a random 4nt P7 barcode is added. It is not clear whether this barcode is actually used and whether it helps to remove PCR artefacts or to assess consistency of the results.

15. Typo: line 330: "enabled"

Reviewer #2 (Remarks to the Author):

I previously reviewed this manuscript for another NPG journal. I have therefore compared this submission with the previous one, and concluded that it is essentially the same paper I reviewed before, and that the authors resubmitted it with minimal changes, most of them in the Discussion, which is shortened. I will therefore repeat my previous comments:

The manuscript by Wang et al describes a method for high-throughput interrogation of enhancer activity of a pre-selected subset of human genomic sequences. The authors limit the assayed sequence space by using ATAC-seq to enrich for open-chromatin regions, which contain most (but not all) known regulatory elements, and assay around 7 million DNA fragments in total. This richly covers the open chromatin regions, enabling fine mapping of key functional parts of the elements and mutations that affect their function.

- One fact that the authors are insufficiently upfront about is that HiDRA is basically a variant of STARR-seq (Arnold et al Science 2013) - indeed, the plasmid used for the assay was obtained from

Alexander Stark, whose laboratory developed STARR-seq. For STARR-seq in human cells, there is an apparently unresolved methodological issue. In Drosophila, there seem to be two classes of enhancers ("developmental" and "housekeeping", Zabidi et al Nature 2015) which are revealed by using different promoter types in the reporter construct. In human, those two classes have not been resolved because the human STARR-seq plasmid prefers to use the CpG-rich bacterial ORI sequence as promoter instead of researcher-chosen ones (https://www.biorxiv.org/content/early/2017/07/17/164590). The authors of this paper do not comment on possible consequences of this for the results presented in this paper and their interpretation.

- The principal difference with respect to the original STARR-seq protocol, which assayed the entire Drosophila genome in an unbiased manner, is the pre-selection of most likely regulatory sequences using ATAC-seq. This idea itself is not particularly novel - Vockley et al (Cell 2015) described an approach they called ChIP STARR-seq in which they performed STARR-seq assay on regions enriched for glucocorticoid receptor binding.

- The ATAC-seq library is dominated by the fragments that overlap predicted promoter sequences, and many of them are among the fragments giving the strongest signal enrichment in the final HiDRA signal. According to the default interpretation of the STARR-seq assay, not questioned by the authors, this would mean that promoter sequences can act as enhancers for the reporter gene promoter when placed in the gene's 3' UTR. This would be an interesting observation, which has not been convincingly demonstrated before. However, since no full-length reporter transcripts are sequenced, how can the authors be sure that placing a promoter-containing fragment in 3' UTR does not results in its - promoter activity? In other words, can the authors exclude the possibility that this results in short non-coding transcripts that start at the cloned promoter (instead of at the reporter gene promoter) and extend into the part of the reporter gene 3' of the cloning site (which is the part targeted by the primer for reverse transcription)?

- In Figure 4, the most active region seems to be the least accessible. Is it because the transcription factor binding shields the sequence from the transposes, or because the DNA input is enriched for mononucleosome-length fragments?

- The motif and GWAS signal analysis give unsurprising results compared what the analysis of ATAC-seq regions themselves would yield, given what is known about the GM12878 cells.

- (cca lines 93-100 in the manuscript) Is the comparison of RNA signal with non-transfected DNA input library the right thing to do? Transfection efficiency can be significantly different for different DNA fragments, depending on both their size and nucleotide composition. Many human promoters

are very GC and CpG rich, unlike the majority of enhancers and a minority of tissue-specific promoters. This may introduce bias if untransfected library is used as baseline. If it is not possible to sequence the transfected DNA library, some proxy checks should be required (e.g. whether longer fragments or more GC rich fragments give systematically higher RNA signal).

- While the correlation of HiDRA signal with the luciferase assays of known enhancers is strong, the fact that heterochromatin regions are modestly enriched in HiDRA signal and that e.g. Polycomb-repressed regions are depleted means two things: first, that some fragments in the plasmid are more accessible/exposed than in their native chromatin context. This is not unexpected, but does lead to false positives if the signal is taken as a measure of what actually happens in the genome. Secondly, and for me more interesting, some DNA fragments, such as Polycomb-repressed fragments, apparently contain enough information to attract Polycomb repression even in the plasmid; I cannot see other reason why they should be further depleted relative to input. These issues should be commented on in the paper.

Reviewer #3 (Remarks to the Author):

The manuscript by Wang et al. describe the use of an episomal reporter assays to directly identify regions from open chromatin that are capable of driving reporter gene expression. The authors accomplish by using the novel approach of inserting an ATAC-seq library, captured from a lymphoblastoid cell line, into a STARR-seq vector and testing activity using a slightly modified STARR protocol. This approach is advantageous due to the use of ATAC-seq as a starting material which can be more cost effective compared to using a whole genome or BAC based library or synthesized oligos. By selecting only ATAC-seq fragments the authors are enriching for elements thought to drive regulatory activity while maintaining a "whole-genome" approach. This allows a large number of elements to be evaluated by the assay providing a comprehensive survey of where episomal assays are capable of characterizing regulatory activity and where the assay underperforms. This knowledge, the strength and limitations of reporter assays, is lacking and dearly needed as the assay gains in popularity. Finally, the authors further extend their assay to recover heterozygote alleles allowing them to evaluate allelic differences in regulatory activity.

The paper is well written, the analysis is comprehensive and thoughtfully executed. The approach presented here is novel within the family STARR based assays and one that will likely represent a significant proportion of STARR-seq assays going forward. This work, the first to my knowledge that uses ATAC-seq, will serve as a valuable blueprint for future studies. Most of my comments are either clarifications that would help the flow of the paper or additional analysis I would be curious to see

and would further strengthen an already strong study. I only have several concerns that I would consider critical.

Critical comments:

-A sticking point for me is the new name (HiDRA) the authors provide for a preexisting assay. I would strongly recommend the authors adhere to the conventional naming and describe their assay as "atac-STARR" or "ca-STARR" (chromatin accessible) coupled with an analysis using SHARPR2. An additional acronym would unnecessarily fragment the field and add confusion to those new to the field.

-Lines 63-66: The authors describe pricing differences between capture-based reporter assays (STARR-seq) and methods that use oligo synthesis as the input source. In its current form, the comparison only accounts for library preparation and not the entire experiment. To perform a proper comparison, one needs to consider both sequencing costs (paired and single-end) as well as uniformity of the libraries which impacts the sequencing depth required for adequate coverage. Price comparisons are always tricky; I would recommend removing it completely, significant simplification of the statement or provide a detailed breakdown of the budget in the supplemental which includes labor, sequencing and all reagent costs.

-Lines 250-255: Specific claims are made regarding the ability to map regions down to the individual nucleotides responsible for activity. There are several things I would like to see to support the observations and claims by the authors. (i) It would be nice to have either an MPRA (using synthesized oligos) or individual luciferase assays of several regions with and without the active regions identified by the SHARPR2. This will demonstrate how well SHARPR2 can pinpoint the active region and more importantly the proportion of signal that region contributes to the overall activity. In other words, are the SHARPR regions responsible for all of the signal or contribute in an additive (or non-additive) manner in conjuncture with additional undetected regions? Without this it is difficult to make the claim on line 222-223 that driver elements are slightly larger than regulatory motifs. It is unclear if this is a biological observation or a limitation of the assay and analysis.

Minor comments and extensions:

-How was the library size of 9.7M estimated? In addition, it would also be helpful to have table in the supplemental describing how many ATAC-seq reads were lost for each filter (e.g. mapping score, mitochondria, etc).

-Figure 1b, it is unclear if this figure is displaying the library from Buenrostro et al. or the HiDRA library. If it is the prior it should be changed to show the fragment distribution for the HiDRA library.

-Figure 1c, if space permits in the figure legend please briefly state where the enhancer and TSS states are taken from (chromHMM?).

-Paragraph at line 145: I found the observation of higher activity of enhancers relative to promoters to be interesting and one that could be unpacked a bit more in the paper. Instead of looking at enrichment of TF sites (Fig S4b) are there specific TFs that contribute to the higher activity observed in enhancers or is there a greater density of TFs? It would seem that you have enough data here to tease this observation apart.

-Line 175: Can you provide a supplemental figure describing the lack of overlap of enriched TFs found in active elements annotated as enhancers and promoters? What elements are enhancer and promoter specific?

-Paragraph at line 195: I would suggest carefully reviewing terminology in this paragraph. The use of in vivo at line 199 should probably read "in its chromosomal context". The use of occupancy in line 201 is suggestive of a physical interaction.

-Paragraph at line 206: Is there any difference in the LTRs between enhancer/promoter and the quiescent/heterochromatin regions that could explain the observed activity differences? For example, are the quiescent LTRs younger and thus less divergent?

-For the driver elements identified by SHARPR2, where are they positioned within the ATAC-seq peaks? (e.g. Is there a trend for the active region to be centered at the summit of the peak)

-Paragraph at line 300: This is a wonderful extension of the dataset but one I fear is underpowered for any general conclusions. What is the depth of coverage for the 16,000 filtered variants? There doesn't appear to be a coverage filter applied to this set. If you were to only take the alleles that were well powered to detect a 20-50% allelic skew how many 'allelic' SNPs are identified?

-What is the relationship between chromatin accessible QTLs (caQTLs), as measured by allelic skew in your plasmid libraries or ATAC-seq library, and allelic skew measured by RNA. Is there a trend where caQTLs tend to show a greater proportion of expression difference between alleles and if so is the direction of effect shared between the caQTL and STARR-seq signal?

Response to reviewers: NCOMMS-18-01211

We wish to sincerely thank all three reviewers for taking their time to provide the very helpful comments on our manuscript. We incorporated many of the comments and suggestions, which have considerably strengthened the paper. Below, we have attached our response to comments and questions raised by the reviewers. Moreover, in our revised manuscript, we include more discussion on the similarities and differences between our assay and STARR-seq.

Regarding the name of the method, we changed the acronym of our assay from HiDRA to HDRA (High-Definition Reporter Assay) to emphasize its conceptual similarity to MPRA (Massively Parallel Reporter Assay). This naming scheme follows other high-throughput reporter assays, including the GPRA (Gigantic Parallel Reporter Assay, doi.org/10.1101/224907). As we discuss in our response to Reviewer #3, we chose a name and acronym similar to MPRA to emphasize the functional similarity between our assay and the family of MPRA approaches, which were developed for testing and dissection of regulatory elements, in contrast to STARR-seq approaches which were developed to discover new regulatory elements rather than to perform high-throughput experimental dissection of regulatory element function.

The reviewer comments are listed in **black font**, while our response is listed in **blue font**. Where we include text from our manuscript, we use either **green font** for text present in our original submission, or **red font** for newly added or edited text.

Our response to Reviewer #1 is on page 1, Reviewer #2 on page 15, and Reviewer #3 on page 22.

Reviewer #1 comments:

In this work, Wang and colleagues developed a new high-throughput reporter strategy to assess enhancer activity. The assay, named HiDRA, combined the ATAC protocol, to enrich for accessible chromatin regions, with the STARR-seq reporter assay. HiDRA thus allow a straightforward and cost-effective approach to assess enhancer activity from putative regulatory regions. The authors demonstrated the efficiency of the assays and developed an algorithm to pinpoint the key regulatory regions ("driver" elements). They also show how the assay could be implemented to assess the effect of regulatory variants.

The HiDRA assay has the potential to easy the study of regulatory regions and to be implemented by researchers interested in gene regulation. Overall the study is well conducted and comprehensive analyses were performed. However, I think several issues should be addressed before the manuscript could be accepted for publication, as detailed below.

Specific remarks

1. The authors claim that they obtained a high level of coverage within the ATAC accessible regions. However, they provided the maximum of coverage (370 fragments per region), which is not really informative (abstract and lines 48 and 81); more than half of the fragments are, actually, represented by less than 10 fragments (line 82). Instead, the authors should provide the average of fragments per region). It will be also important to provide a supplemental figure sowing the coverage distribution among the ATAC peaks.

We thank the reviewer for this comment. The text discussing “370 fragments per region” refers to the regions used for the SHARPR2 algorithm to map short driver elements. The distribution of unique fragments per tiled region has a long tail (many regions with few fragments due to random fragmentation), so we believe it is most meaningful to report the number of unique regions with high fragment coverage that can be used for high-resolution mapping. We have now changed the text to read:

> “Our selection approach resulted in highly-overlapping fragments (~32,000 regions covered by 10+ unique fragments, ~12,500 by 20+ fragments), enabling us to pinpoint “driver” regulatory nucleotides that are critical for transcriptional enhancer activity.” (lines 47-50)

In addition to the text changes, we have added a plot showing the distribution of unique fragments per tiled region used for as an input to SHARPR2. This is included as Supplemental Figure S8.

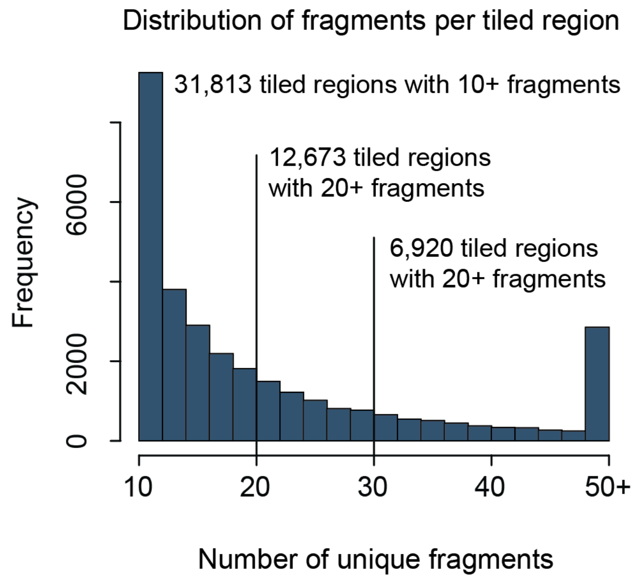

Supplemental Figure S8: Number of unique fragments per tiled region for high-resolution mapping.

Regarding the reviewer's comment on the depth of coverage within ATAC peaks, Supplemental Figure **S1a** in the original submission shows the proportion of regulatory elements (active TSS, active enhancer and ATAC peaks) with 1+, 2+ and 5+ unique HDRA fragments. In our revised manuscript, we also include a second set of plots that show the distribution of unique fragments in each class of regulatory elements. This new panel has been added as Supplemental Figure **S1b** and is reproduced below.

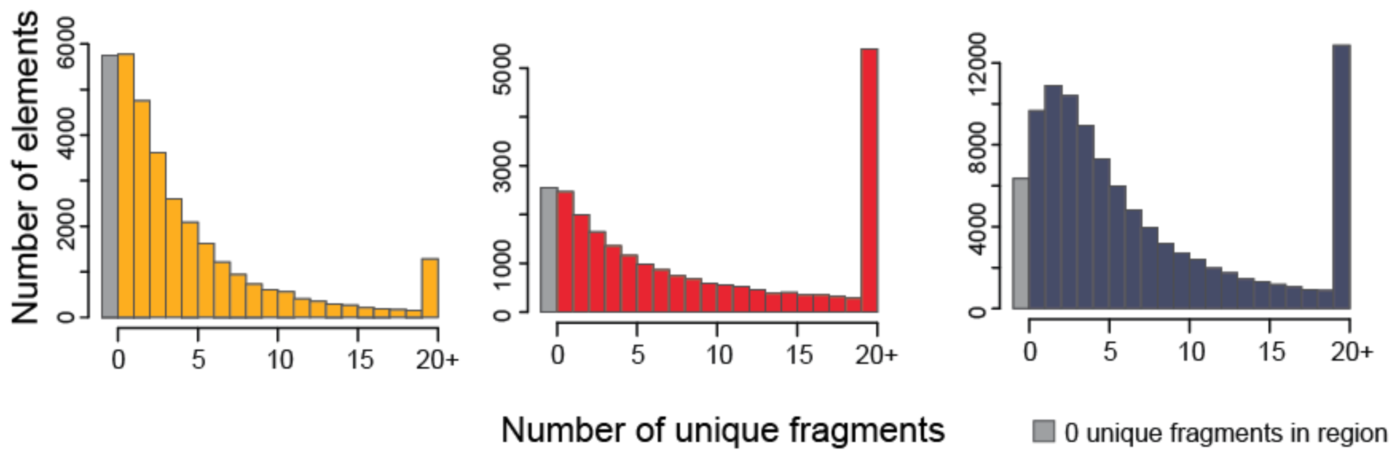

2. The values provided at the end of the introduction ("~95,000 active fragments clustering in ~65,000 regions showing significant regulatory function"; "We discover ~13,000 high resolution driver elements") are somehow confusing because the different terms are not yet defined. These paragraph should be re-writing in a more clear way.

We have re-written the text to be more clear and have pasted the amended text below:

> "We applied HDRA to infer genome-wide regulatory activity across ~7 million DNA fragments preferentially selected from accessible chromatin in the GM12878 lymphoblastoid cell line, resulting in ~65,000 discrete genomic regions showing significant regulatory function." (lines 43-45)

And

> "Our selection approach resulted in highly-overlapping fragments (~32,000 regions covered by 10+ unique fragments, ~12,500 by 20+ fragments), enabling us to pinpoint "driver" regulatory nucleotides that are critical for transcriptional enhancer activity. We discover ~13,000 of these high-resolution driver elements, which are enriched for regulatory motifs and evolutionarily conserved nucleotides, and help predict causal genetic variants underlying disease from genome-wide association studies." (lines 47-51)

3. From the results provided by the authors it is difficult to assess how well the ATAC peaks are represented in the HiDRA library. Figure 1d shows a screenshot comparing published ATAC-seq with the HiDRA library, but this is not sufficient. The authors should provide a correlation plot between the two datasets in order to pinpoint (or exclude) potential bias.

We thank the reviewer for this comment. Supplemental Figure **S1a** shows the proportion of ATAC-seq peaks that contain 1+, 2+ and 5+ unique HDRA fragments, ranked by strength of the peak (this panel has been pasted below). In total, 94.1% of ATAC-seq peaks contain at least one unique HDRA fragment. We also observe that the strongest ATAC-seq peaks have a greater number of unique fragments: 97.5% of the top 10,000 ATAC-seq peaks contain 5+ fragments, compared to 54.0% of all ATAC-seq peaks. As we mention in the response to point #1, we have also added a new panel (Supplemental Fig. **S1b**) showing the distribution of unique fragments in enhancers, TSSs, and ATAC-seq peaks:

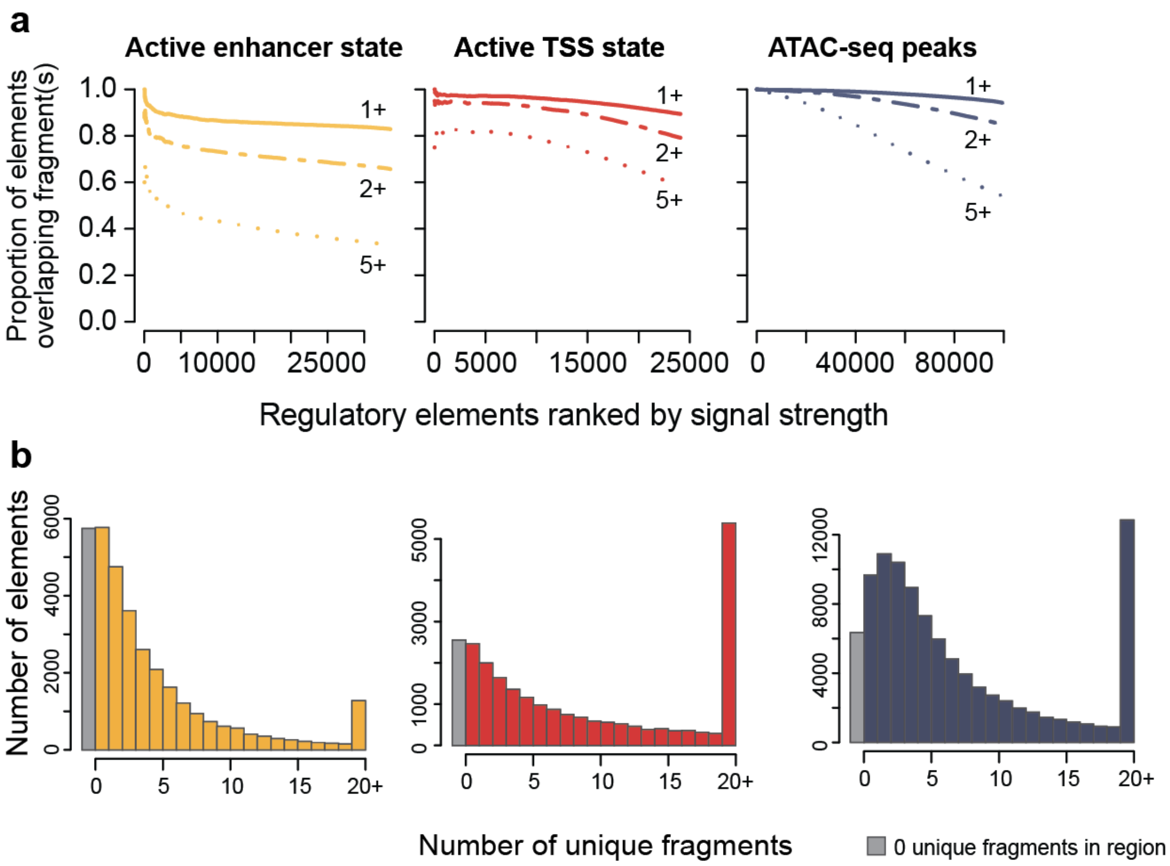

Supplemental Figure S1: HDRA coverage is greater for highly active regulatory elements. (a) ChromHMM-defined active enhancer and active TSS chromatin states were ranked by H3K27ac signal strength, and ATAC-seq peaks were ranked by density of ATAC-seq reads from Buenrostro et al. (2013). Solid, dashed and dotted lines correspond to coverage with at least 1, 2 and 5 unique HiDRA fragments. (b) Number of unique HDRA fragments in ChromHMM-defined active enhancer and active TSS elements, and ATAC-seq peaks.

Additionally, as the reviewer suggested, we include a new correlation plot between the strength of an ATAC-seq peak and the representation of fragments within HDRA library, finding a strong correlation (Spearman coefficient = 0.69) between ATAC-seq peak strength and the number of unique HDRA fragments covering the peak:

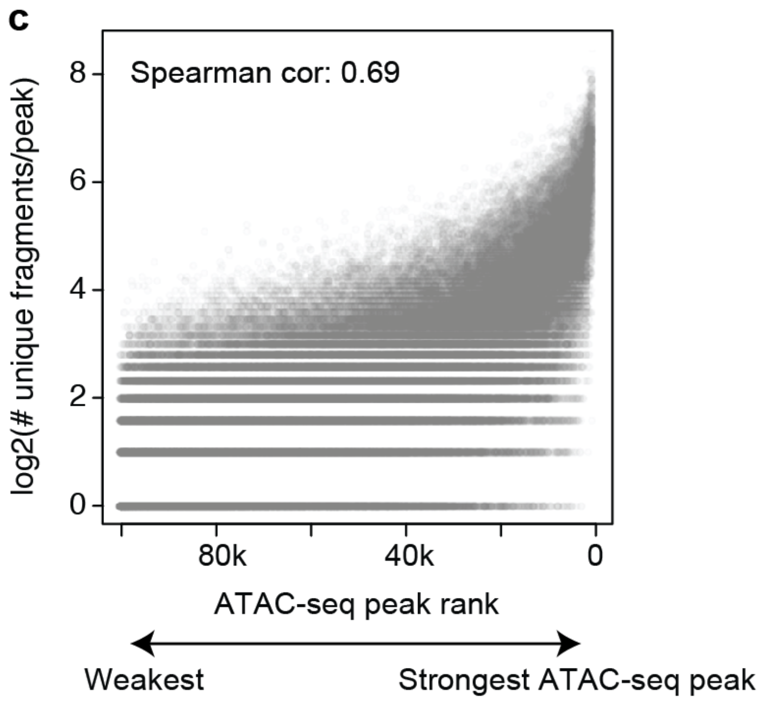

Supplemental Fig. S1c: Positive relationship between ATAC-seq peak strength and number of unique HDRA fragments. Discrete bands at bottom of scatterplot represent peaks with 1-6 unique fragments.

4. One potential concern of the HiDRA assay is that the results might be biased by the relative accessibility of each region, which does not necessarily correlate with the strength of enhancer activity. For instances, a highly accessible region will be overrepresented in the library and therefore provide a more reliable result than a less accessible region. One way to assess this potential bias will be to compute the % of active regions in function of chromatin accessibility (obtained from the ATAC-seq signal).

The reviewer raises an important issue in the field of genomics, where features (e.g. ChIP-seq peaks or gene transcripts) with greater read coverage have more statistical power for discovering differential activity. This issue generally does not impact enhancer reporter assays that rely on *in vitro* synthesis (e.g. MPRAs), as these assays have a very limited input library complexity, leading all fragments to be well-represented.

In our assay, the input library complexity is much higher than traditional MPRA. This provides major benefits over MPRA-style *in vitro* synthesis-based assays, notably more regions are tested in one experiment, and regions are covered by more unique fragments. However, the trade-off of high input complexity is that true enhancers with very weak coverage may be missed due to a lack of power. In our manuscript, we mitigate this trade-off by first grouping partially-overlapping fragments into “fragment groups”. These fragment groups have higher coverage than individual fragments and thus increase statistical power to detect reporter activity.

As we expect based on literature from differential gene expression analysis (e.g. PMIDs 20979621 and 25516281), true active fragment groups with greater library coverage are more likely to be called as significantly active. Our dataset follows the prior literature on this point, and we observe a strong positive relationship between higher library representation (x-axis of **panel a** below) and likelihood of being called as active (y-axis). This trend also persists when considering only regulatory elements (enhancers & active TSS, **panel b**).

Importantly, the quantified activity of a fragment (i.e. the RNA/DNA ratio) is not biased by DNA abundance. Across all fragment groups, we notice a slight positive relationship between DNA abundance and RNA/DNA ratio (**panel c**), but we believe this arises because regulatory elements (e.g. enhancer/promoters) have higher overall DNA abundance. When we consider only ChromHMM-defined active enhancer and active TSS elements, we see no relationship between DNA abundance and fold-change (**panel d**), indicating that the quantified activity of a fragment in our assay is not biased by its representation in the DNA library.

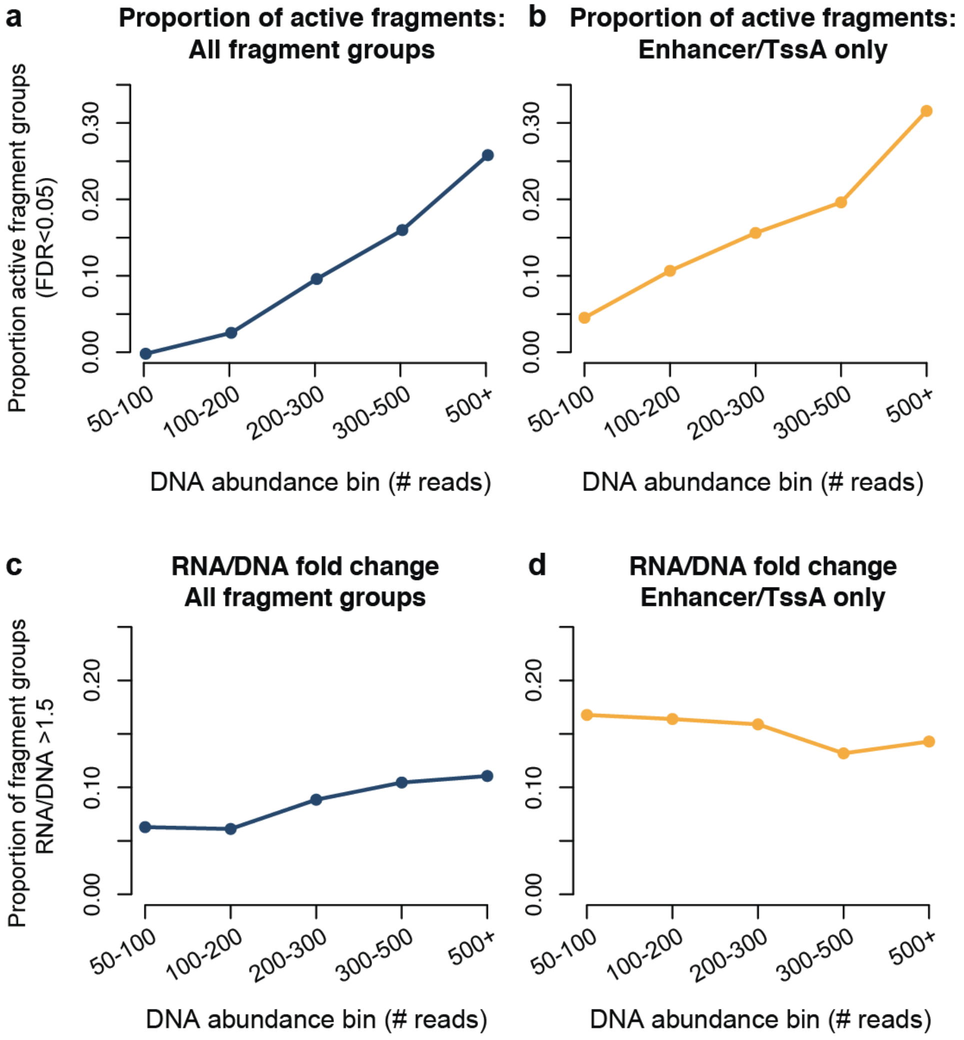

5. As the authors shows in figure 1b, the fragment size of the HiDRA library display a bi-modal distribution (as expected). It will be of potential interest to assess the relative enhancer activity between the small and large fragments. Are active fragments preferentially found within the smaller or larger sizes? For a given driver element, is the length of the fragment somehow affecting the enhancer strength?

To test this suggestion, we re-analyzed our data to call active fragments without grouping closely overlapping fragments together into fragment groups, as we had presented in the manuscript. We present the results below:

When considering all fragments, we observe an oscillating length-dependent effect of fragment length on reporter activity (**panel a, below**). The original characterization of ATAC-seq patterns found that peaks of the distribution are enriched for repressive heterochromatin, while the dips are enriched for activating enhancers and promoter elements (Fig. 2b from Buenrostro *et al.* Nature Methods 2013). Consistent with this, we observe that the oscillating pattern from **panel a** matches the oscillating proportion of enhancer/TSS-derived fragments (**panel b, below**).

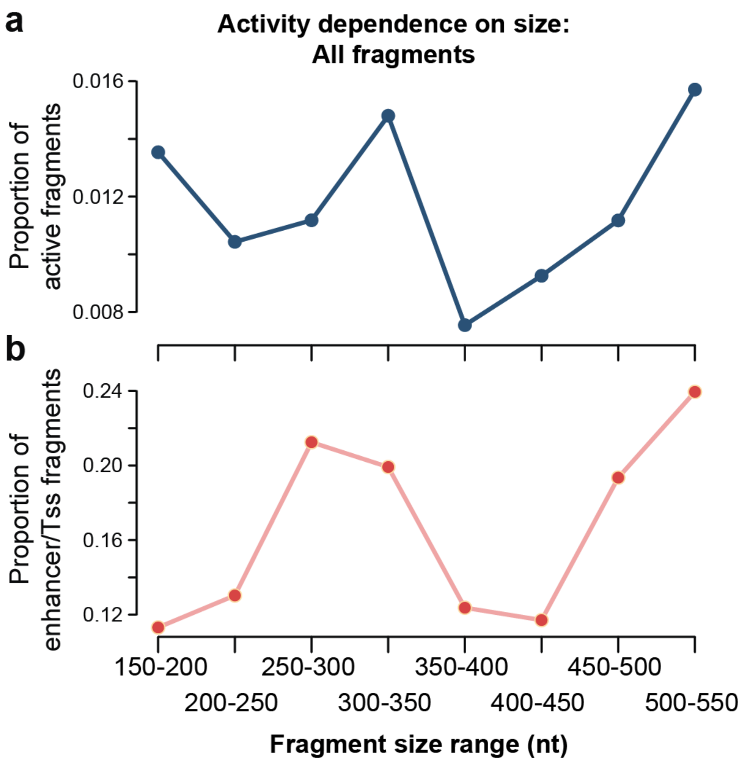

Figure legend: (a), Proportion of active fragments as a function of fragment size. (**b**), Proportion of fragments from endogenous ChromHMM-defined active enhancer or active TSS regions as a function of fragment size.

We also followed the suggestion of the reviewer to assess reporter activity conditioned on the presence of driver elements. As expected, fragments that contain driver elements are substantially more likely to be called as significantly active (16-fold compared to all fragments, **panel c** below). We also observe that the oscillating pattern observed above is attenuated, and that smaller fragments containing driver elements are more likely to be activating than larger fragments (**panel d**). We speculate this latter effect is due to either of two mechanisms: (i) after conditioning on an active driver element, an increased fragment length increases probability of overlapping a repressive element, or (ii) enhancer activity is context-dependent, and addition of extra activating regulatory sites can reduce activity. Indeed, MPRA studies on synthetic regulatory elements have shown that the addition of activating TF binding sites can weaken expression (e.g. PMID 27965290).

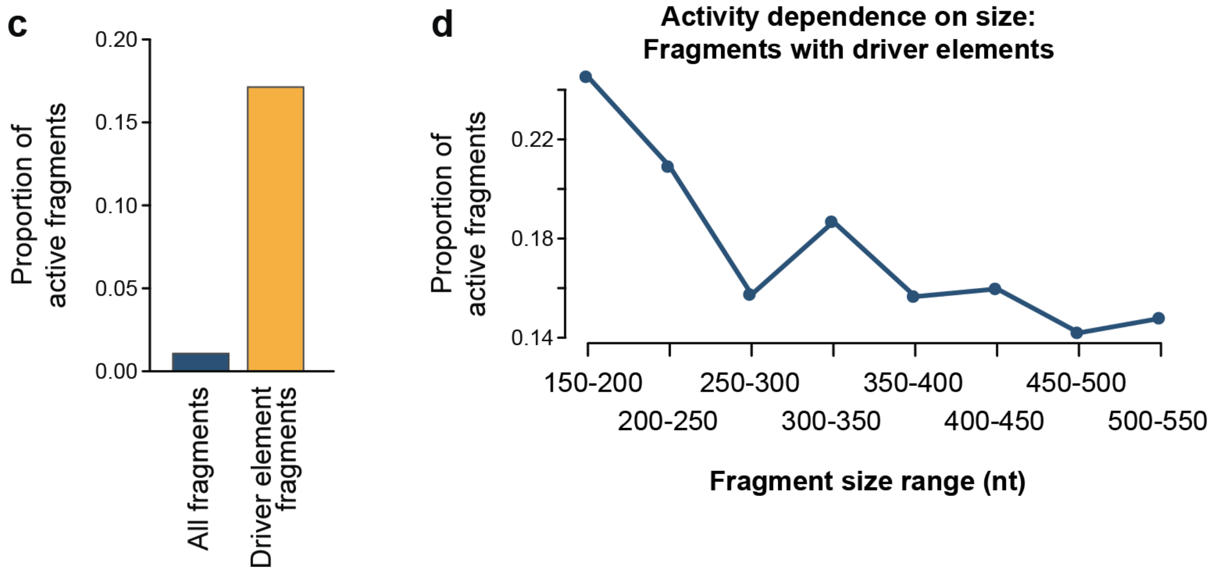

Figure legend: (c), Fragments overlapping SHARPR2 driver elements are 16-fold more likely to drive activity compared to all tested

fragments. **(d)**, Shorter fragments overlapping driver elements are more likely to drive activity compared to longer driver-containing fragments.

6. Given the recent finding that many core promoters display enhancer activity in high-throughput reporter assays (e.g. Nat Genet 49(7): 1073-1081; Nat Methods 15(2): 141-149; Nature 518(7540): 556-559; Genome Res 26(8): 1023-1033), it will be interesting to precisely know what percentage of the regions overlapping TSS of coding genes actually display enhancer activity (instead of using the chromatin state definitions of promoters and TSS-flanking regions, which are ambiguous) and how this percentage compare with the TSS-distal regions.

To address this comment, we compared the proportion of fragment groups overlapping coding gene TSSs ("TSS proximal", within 2kb of annotated TSS) to TSS distal (>2kb from TSS). We find that TSS proximal fragments are ~4X more likely to display enhancer activity than the set of all TSS distal fragments (4.8% vs. 1.1%, panel below).

For comparison, fragment groups overlapping ChromHMM-defined enhancer or TssA elements both display greater enhancer activity than TSS proximal fragments (6.0% and 10.5% vs. 4.8%). While TSS proximal regions and ChromHMM-annotated TssA elements may instinctively appear to contain similar sets of regions, we believe that ChromHMM-annotated TssA elements have substantially higher enhancer activity for two reasons: (i) ChromHMM annotations are more precise: the +/- 2kb window around an annotated TSS will include many inactive regions, while ChromHMM is restricted by active histone modifications, and (ii) ChromHMM annotations are specifically present at active promoters, whereas the TSS proximal regions are defined for all genes (active and inactive) regardless of expression.

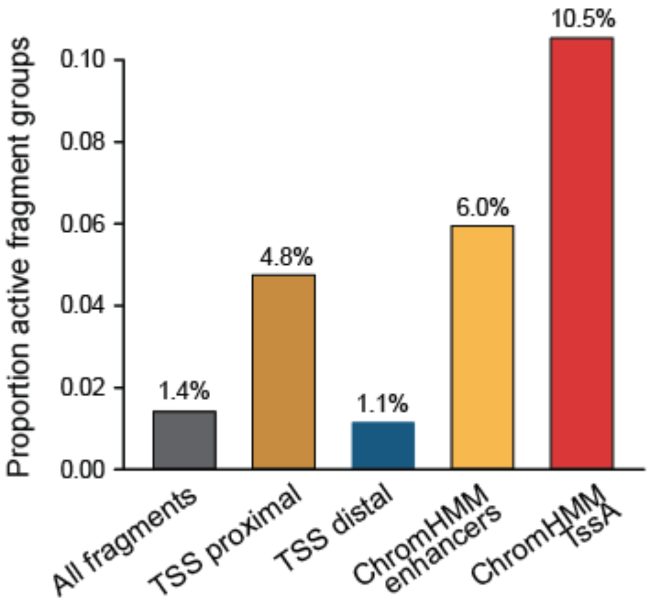

Legend: Proportion of active fragment groups for different classes of genomic elements.

7. Their findings that some active elements originate from heterochromatin-associated states should be discuss in the light of two recent whole genome reporter assays (Nat Methods 15(2): 141-149; Genome Biol 18(1): 219) reporting similar findings.

We have added these two references in the section, and re-write the sentence as:

> In fact, consistent with recent studies[24,25], approximately half of active HDRA regions (52%) showed endogenous epigenomic signatures characteristic of repressed and inactive chromatin states, including Quiescent, Repressed Polycomb, Weak Repressed Polycomb, and Heterochromatin. (lines 183-185)

8. Related to the above issue, it is surprising that active regions are detected within heterochromatin regions as these are not expected to contain ATAC peaks and therefore should not be present in the HiDRA library. Could be possible that these "active regions" come from background regions with low coverage and therefore less accurate calling? In any case this issue should be discussed.

We thank the reviewer for raising this point and apologize for not explaining more clearly in the manuscript. Regarding the first point where heterochromatin fragments should not be in the HDRA library, we wish to point out that the ATAC-seq transposition reaction fragments the entire genome. The Tn5 transposase used in ATAC-seq is in fact taken from Illumina Nextera kits where it is used to prepare libraries for whole-genome sequencing. The utility of Tn5 for open chromatin mapping comes from its preferential insertion within regions of open chromatin (Buenrostro *et al.* 2013). Therefore, under the specific conditions of the ATAC-seq reaction (e.g. limiting amount of Tn5 transposase, short reaction time), this preference can be leveraged to enrich for open chromatin fragments. However, the majority of fragments from an ATAC-seq experiment are still derived from endogenously inactive chromatin regions (e.g. see left column of Fig. 3 in manuscript).

ATAC-seq and ChIP-seq signal tracks presented in publications often give the impression that reads are derived almost exclusively from open chromatin peaks, however this is a consequence of the signal processing steps applied. The y-axis on these signal tracks often represents either fold-change vs. no-IP input (for ChIP-seq), or the -log(p-value) likelihood of a signal, which reduces the signal from fragments outside of peaks. Finally, other groups have also observed that most reads are not derived from peaks when they consider all reads from an epigenome mapping experiment. For example, Vockley et al. (PMID 27565349) notes that only 6% of fragments from a glucocorticoid receptor ChIP pulldown mapped within called GR peaks ("*Of the cloned fragments, 6% mapped to the 27,432 called GBSs*.", page 2 of Vockley *et al.*, left-hand column).

To address the reviewer's second point, we do not believe that active regions from heterochromatin are a false positive signal from low DNA input, for the following three reasons. First, the DESeq2 differential activity algorithm requires a higher burden of differential activity for fragments with low input DNA, reducing the false positive rate (e.g. PMID 20979621). Second, we observe that active heterochromatin fragments are enriched for TF ChIP-seq peaks compared to a background of all heterochromatin regions (**Fig. 3c**), suggesting some of these regions are functionally important in the cell. Finally, after matching for DNA library representation, heterochromatin fragments show higher rates of enhancer activity compared to Polycomb-repressed and Quiescent-derived fragments (**panel below**). If the signal from heterochromatin were entirely artificial, we would expect no difference in activity patterns between fragments derived from these three chromatin states.

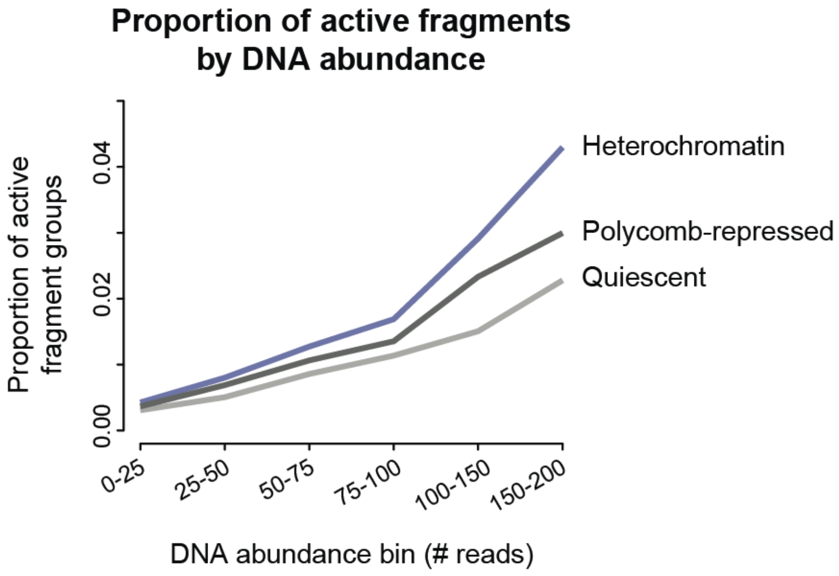

Panel: Heterochromatin-derived fragment groups are more likely to be active compared to Polycomb-repressed and Quiescent-derived fragment groups, after controlling for DNA abundance in library.

9. The identification of driver elements is one of the key aspects of the HiDRA assay. However, the functional relevance of these driver elements should be tested by classical reporter assays where the 'driver" region is either tested alone or deleted from the whole enhancer region.

We thank both Reviewer 1 and Reviewer 3 for suggesting additional experimental validation for our driver element predictions using perturbation assays. We now include such experimental validation of reporter assays with the sequence of the 'driver' region shuffled, and we show the results strongly support our conclusions (see point #7 below). We used MPRA-based validation, as recommended by Reviewer 3, as this more directly matches the experimental procedures utilized here.

In addition to these shuffled nucleotide perturbations, we also evaluate the effect of single-nucleotide genetic perturbations in our predicted driver regions (point #4 below), and we compare our driver region inferences with activity scores from the Sharpr-MPRA study. In total, now present seven lines of evidence to support the functional relevance of driver elements, including data from three separate MPRA experiments. We present these below:

1) (*present in original submission*) Driver elements are enriched for relevant motifs corresponding to TFs active in GM12878, compared to a background set of all tiled regions (used as input to SHARPR2 to discover the driver elements). This was originally presented as **Fig. 4c** and **4d** and is copied below.

2) (*present in original submission*) Driver elements are significantly more likely to be evolutionarily conserved compared to randomly selected elements of equal size within tiled regions used as input to SHARPR2 to discover the driver elements. This indicates that driver elements are significantly more likely to be functionally important and therefore under purifying natural selection. This was originally presented as **Fig. 4e** and is copied below.

3) (*present in original submission*) Driver elements are significantly more likely to overlap fine-mapped GWAS SNPs associated with autoimmune diseases, compared to randomly selected elements of equal size within tiled regions. This indicates that genetic variants within driver elements are more likely to be functionally relevant and therefore be associated with autoimmune disease risk. This was originally presented as **Fig. 5a** and **5b** and is copied below.

4) (*newly added as Fig. 4f*) Consistent with our results showing that driver elements are enriched for fine-mapped autoimmune SNPs (Fig. 5a), we observe that SNPs overlapping driver elements exhibit a significantly stronger allelic skew by MPRA (difference in MPRA reporter activity between the two alleles) compared to two background sets: SNPs in tiled regions used to discover the driver elements, and the entire set of SNPs tested from the genome. This indicates that SNPs within driver regions are more likely to disrupt enhancer activity than those outside drivers, indicating that driver elements are important for enhancer activity.

5) (*newly added as Supplemental Fig. S11a*) We shifted the position of driver elements both upstream and downstream, and we find that these adjacent regions have weaker evolutionary conservation than true driver elements. This suggests driver elements are positioned precisely at regions of functional importance that are under purifying natural selection.

6) (*newly added as Supplemental Fig. S11b*) We compared driver elements identified in our study with enhancer functional importance scores calculated by Ernst *et al.* 2016 (Nature Biotechnology). Ernst (2016) presented the original Sharpr-MPRA algorithm for high-resolution mapping of regulatory nucleotides, and comparing against this study offers an independent validation of our driver elements using an MPRA-based platform. We observe:
    a) Driver elements from our study have higher Sharpr-MPRA activity scores compared to adjacent regions. This agrees with corresponding evolutionary conservation analyses, and indicates that driver elements are positioned at the region of functional importance
    b) This enrichment of Sharpr-MPRA activity is observed in comparisons with both cell types tested by Ernst *et al.* (2016): K562 and HepG2
    c) Consistent with similarity between cell types, our driver elements (identified in GM12878

lymphoblastoid cells) have higher activity in K562 (chronic myelogenous leukemia line) than HepG2 (hepatocellular carcinoma line)

d) Only HDRA driver elements that carry a TF motif active in either K562 or HepG2 show activity in the corresponding cell type, providing a mechanistic explanation for the similarities in activity.

7) (*newly added as Supplemental Fig. S11c*) We predict that disrupting the nucleotide sequence in driver elements will reduce or eliminate enhancer activity. Kheradpour *et al.* (2013) used MPRA to randomly shuffle the sequence of a set of regulatory motifs, and tested activity of wild-type and shuffled sequences in both K562 and HepG2. Of Kheradpour et al.'s tested elements, four showed activity in either K562 or HepG2 and also overlapped driver elements predicted in our study. Disrupting motifs all four driver elements led to significant decreases in enhancer activity in either K562, HepG2 or both cell lines. This result indicates that experimentally disrupting a driver element leads to a reduction or complete loss of enhancer activity.

Figure panels corresponding to these seven lines of evidence are presented in order below:

Figure for evidence #1 (present in original submission as Figs. 4c and 4d):

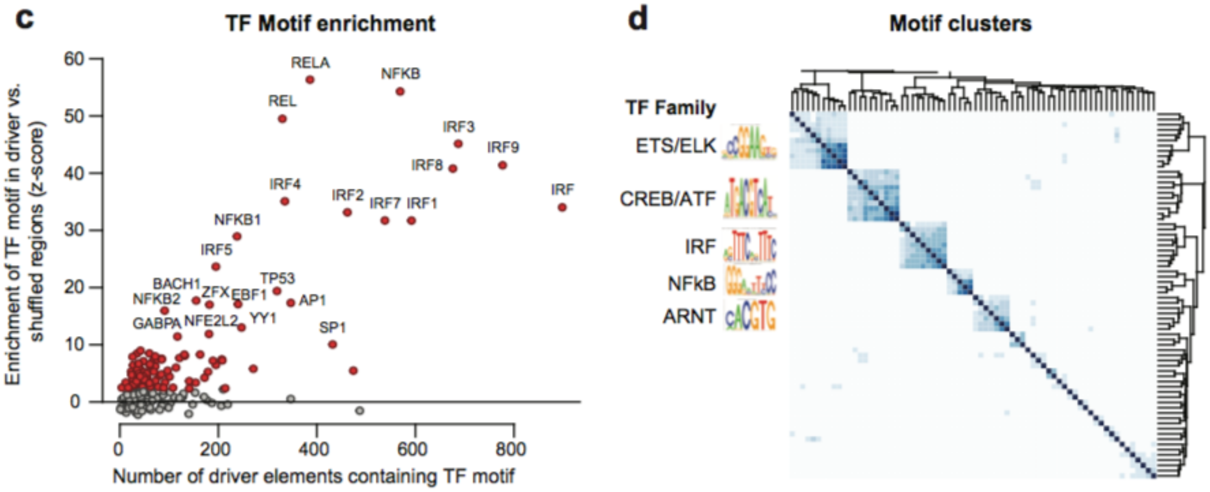

Figure for evidence #2 (present in original submission as Fig. 4e):

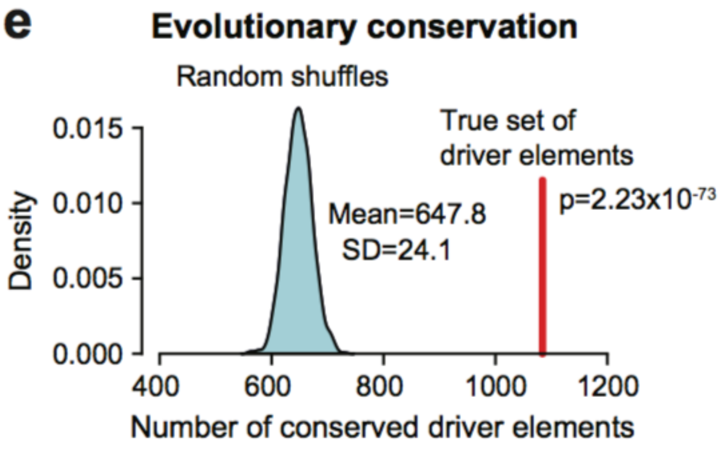

Figure for evidence #3 (present in original submission as Fig. 5a):

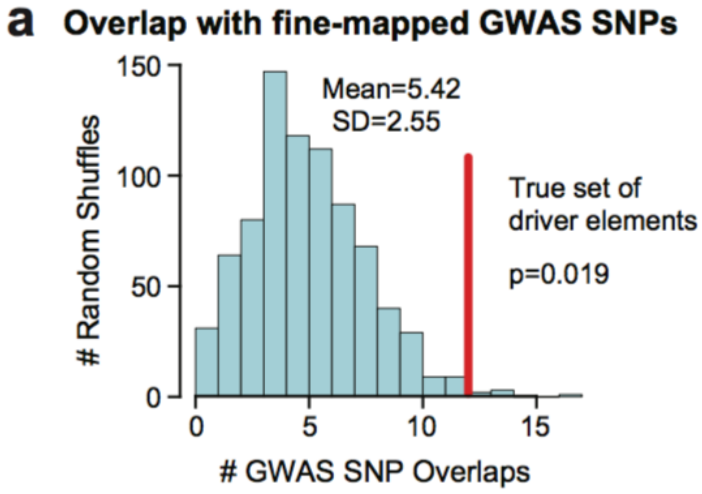

Figure for evidence #4 (newly added as Fig. 4f):

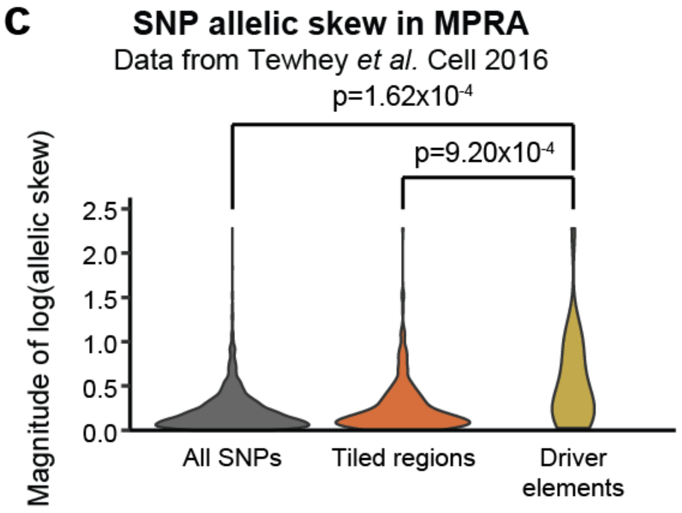

Figure for evidence #5 (newly added as Fig. S11a):

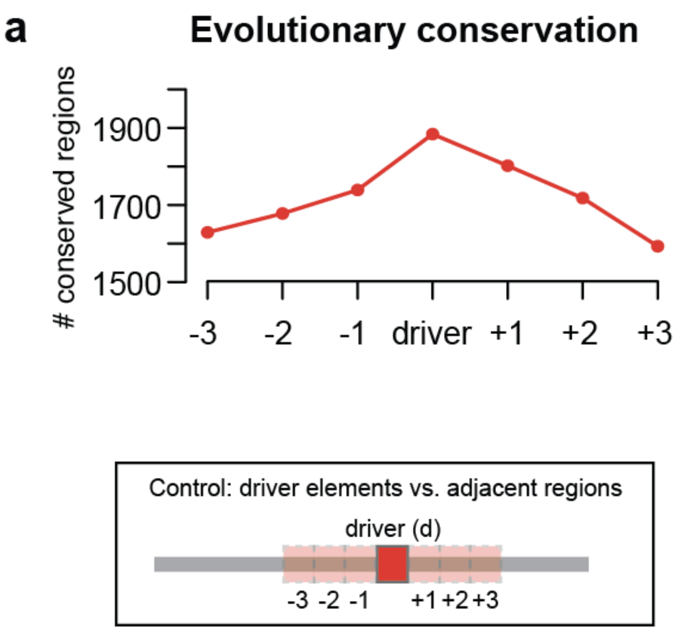

Figure for evidence #6 (newly added as Fig. S11b):

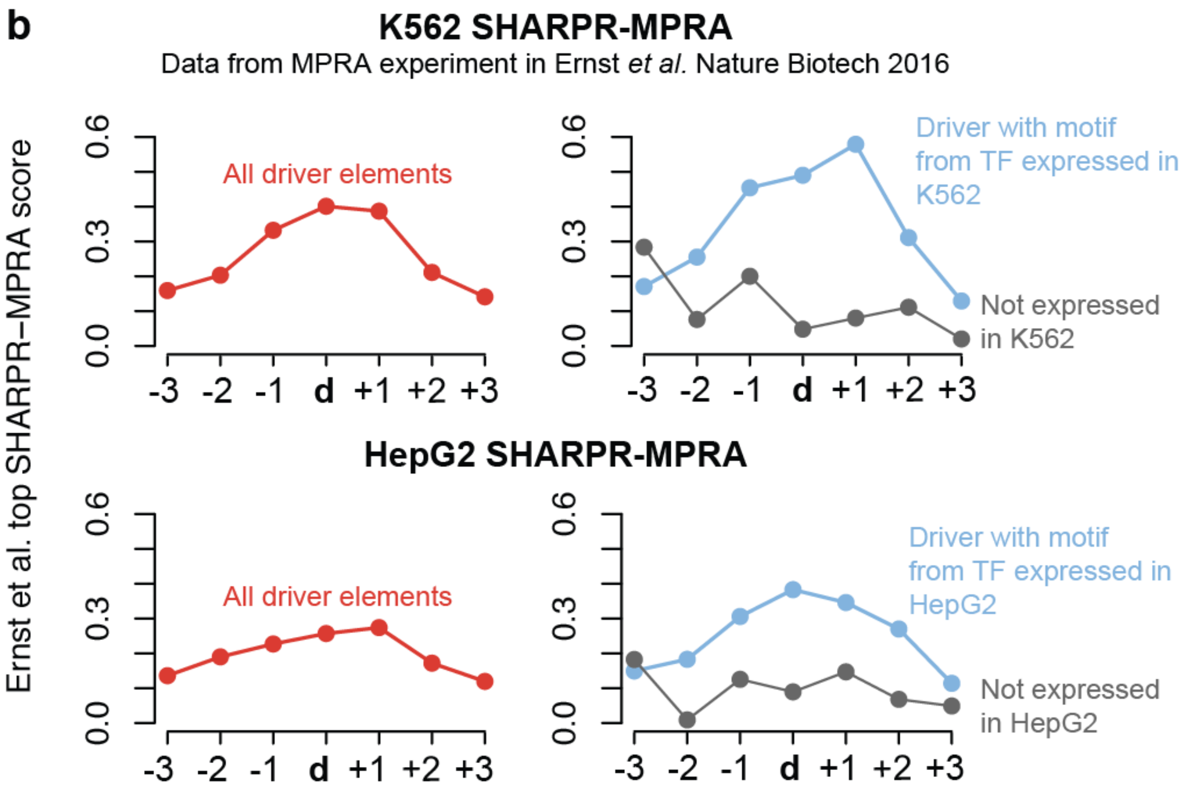

Figure for evidence #7 (newly added as Fig. S11c): Values in cells correspond to activity of MPRA element containing true sequence vs. MPRA element containing shuffled sequence, followed by p-value of the difference across replicates.

c **Shuffled elements by MPRA**

Data from MPRA experiment in Kheradpour *et al.* Genome Research 2013

Driver element (nt)	MPRA shuffled element (nt)	Mutual overlap	K562 median scores (p-value)	HepG2 median scores (p-value)
chr12:76371849-76371904 (56nt)	chr12:76371893-76371906 (14nt)	12nt	1.59 vs. 1.39 (p=0.62)	4.22 vs. 1.66 ($p=1.48\times10^{-9}$)
chr5:149185564-149185589 (26nt)	chr5:149185566-149185576 (11nt)	11nt	0.79 vs. -0.32 ($p=8.70\times10^{-3}$)	1.44 vs. 0.58 ($p=1.32\times10^{-4}$)
chr5:54040333-54040401 (69nt)	chr5:54040375-54040385 (11nt)	11nt	1.35 vs. 0.75 (p=0.029)	1.21 vs. 0.32 (p=3.40x10-3)
chr11:48028283-48028351 (69nt)	chr11:48028311-48028328 (17nt)	17nt	1.27 vs. -0.09 ($p=1.71\times10^{-3}$)	Not active in HepG2

10. The example of the SNP rs2382817 (Figure 6) is awkward. Indeed, the risk allele (A) is associated with increased enhancer activity, but lower expression of the associated gene (TMBIM1). Because this SNP is present in the GM12878 cell line, the authors could directly assess the allele-specific expression of the gene. In any case, this apparent contradiction should be explained.

We believe there are two main explanations for this apparent contradiction: (i) rs2382817 has additional eQTL targets (e.g. PNKD, APRC2, GPBAR1 are significant targets of rs2382817 in other tissues. In particular, the genotype-dependent expression pattern of GPBAR1 varies depending on tissue). It is possible that rs2382817 contributes to the regulation of another nearby gene. (ii) The regulatory element containing rs2382817 is repressive for TMBIM1 expression at the endogenous locus, but activating at the reporter level. The repressive “enhancer” element scenario has been experimentally demonstrated in the literature. For example, Claussnitzer *et al.* (PMID 24439387) showed that the rs4684847 risk allele leads to greater PRRX1 binding and repressed PPARG2 expression CRISPR/Cas9 editing (Fig 4 and Fig. S4 of Claussnitzer *et al.*). Gao *et al.* (PMID 29205154) recently showed that deletion of the 135-bp region in an ATAC-seq peak surrounding rs9920291 (associated with height by GWAS) lead to increased activity of the nearby CHSY1 gene (Fig. 6 of Gao *et al.*). However, given that we are unable to distinguish between these possibilities in our current manuscript, we have removed the text and figure panel on rs2382817 acting as an eQTL for TMBIM1 to avoid confusion.

11. The authors should provide the list of the 880 allelic regions along with the relative allele activity as a supplementary dataset.

We now provide the list of 880 allelic regions as a Supplementary Dataset.

12. The list of active fragments and driver elements should also be provided as a supplementary dataset (in addition to the UCSC link).

We now provide these two lists, and a list of tiled regions used to discover driver elements, in BED format as Supplementary Datasets.

13. In the Methods section, the size selections performed by gel excision and AMPure beads during library preparation should be indicated.

We apologize for this oversight, we appended the Methods section to read:

> “Size selection of ATAC-seq fragments was performed by gel excision using a razor blade to select fragments between 150-500nt.”

and

> “PCR products were cleaned up using two rounds of AMPure bead selection (0.8X ratio of

beads to input) to size-select against small (<150nt) fragments".

14. During the library construction a random 4nt P7 barcode is added. It is not clear whether this barcode is actually used and whether it helps to remove PCR artefacts or to assess consistency of the results.

For the core HDRA assay to perform high-resolution dissection of regulatory activity, the random 4nt barcode is not necessary (but can be helpful to distinguish unique copies of the same genomic fragment for replication). We use the random 4nt P7 barcode for SNP allelic imbalance analyses, where we use a unique identifier of fragment position (chr:start-end) and 4nt P7 barcode to link long reads for fragment genotype to short reads for fragment abundance (see Fig. **6a**, copied below).

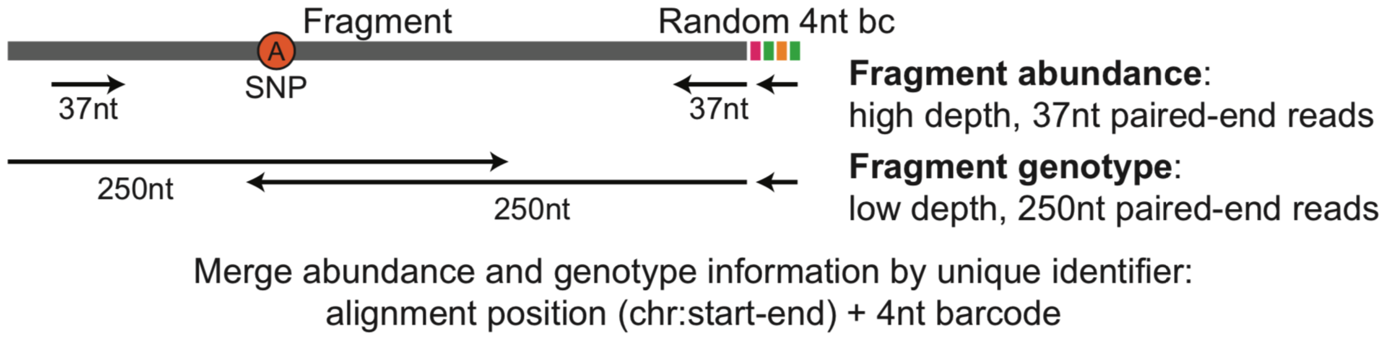

15. Typo: line 330: "enabled"

We have fixed this typo and we thank the reviewer for catching this.

Reviewer #2 (Remarks to the Author):

I previously reviewed this manuscript for another NPG journal. I have therefore compared this submission with the previous one, and concluded that it is essentially the same paper I reviewed before, and that the authors resubmitted it with minimal changes, most of them in the Discussion, which is shortened. I will therefore repeat my previous comments:

The manuscript by Wang et al describes a method for high-throughput interrogation of enhancer activity of a pre-selected subset of human genomic sequences. The authors limit the assayed sequence space by using ATAC-seq to enrich for open-chromatin regions, which contain most (but not all) known regulatory elements, and assay around 7 million DNA fragments in total. This richly covers the open chromatin regions, enabling fine mapping of key functional parts of the elements and mutations that affect their function.

- One fact that the authors are insufficiently upfront about is that HiDRA is basically a variant of STARR-seq (Arnold et al Science 2013) - indeed, the plasmid used for the assay was obtained from Alexander Stark, whose laboratory developed STARR-seq.

We thank the reviewer for pointing out that the distinction between our assay and STARR-seq was not made sufficiently clear in the text. HDRA falls between two families of methods seeking to profile regulatory elements: MPRA-based methods, and STARR-seq based methods:

- STARR-seq was developed for **discovering** enhancers *de novo*. In the initial paper describing STARR-seq, the authors compare STARR-seq to ChIP-seq and DHS-seq, two other methods used for the systematic discovery of putative regulatory elements (e.g. "Here, we present STARR-seq, which complements ChIP-seq and DHS-seq as the third principal method to study transcriptional regulatory elements in entire genomes.", Arnold et al, Science 2013).
- By contrast, MPRA-seq methods were developed for the "**systematic dissection**" and "**functional analysis**" of **candidate** enhancer regions, which were predicted by other methods (Melnikov *et al.* Nature Biotechnology 2012).

Between these two families of methods, we believe that HDRA aligns closely with MPRA-based methods, rather than the STARR-seq family, even though we use the STARR-seq vector to test the activity of DNA fragments. HDRA is the only method, along with Sharpr-MPRA, that allows high-throughput dissection of putative regulatory regions in a massively parallel high-throughput way. For this reason, we named our assay the "High-Definition Reporter Assay" (HDRA) to highlight its similarity to the "Massively Parallel Reporter Assay" (MPRA). In our manuscript, we have tried to give credit in our paper to both STARR-seq-based methods (e.g. lines 21-22, 25-28, 60, 382-392, 442-445) and MPRA-based methods (e.g. lines 17-25, 230-232, 372-381), rather than focusing solely on STARR-seq, as both the motivation and the accomplishments of our method stems on both families of methods.

Further, in our revised manuscript, we add two additional paragraphs in the discussion that (i) highlight the point that HDRA is more conceptually aligned with MPRA, and (ii) highlight the differences between HDRA and STARR-seq. We copy the two paragraphs below:

> (*Comparison vs. MPRA, lines 372-381*)
> "Currently, the only other method that enables the high-resolution dissection of thousands of putative regulatory regions is Sharpr-MPRA[13]. Both Sharpr-MPRA and HDRA seek to map regulatory regions at high resolution, but they differ in key several respects: Sharpr-MPRA selects regions based on prior computational predictions, requiring microarray-based synthesis. This *in vitro* synthesis step limits the number of nucleotides and regions tested by Sharpr-MPRA (4.6Mb in 15,720 regions for Sharpr-MPRA vs. 46.3Mb in 31,813 regions for HDRA). Moreover, Sharpr-MPRA uses fixed 5-bp increments (vs. random increments for HDRA, stemming from the transposase fragmentation pattern), 145-bp fragments (vs. random lengths for HDRA, 335-bp on average), and Sharpr-MPRA tests elements upstream of the TSS using 3'UTR barcodes (vs. testing elements downstream, thus enabling them to serve as their own barcodes)."
>
> (*Comparison vs. STARR-seq, lines 382-392*)

> "The HDRA approach has many benefits compared to regular STARR-seq. HDRA preferentially tests the activity of fragments derived from open chromatin, and the HDRA library we developed achieves 130-220X more highly covered regulatory elements than random genome fragmentation (**Fig. 1c**). This facilitates both higher confidence mapping of regulatory elements, which will have a greater number of unique fragments, and facilitates the high-resolution mapping of regulatory elements by comparing the relative activity of partially-overlapping fragments. This latter application using our newly developed SHARPR2 algorithm permits the genome-wide mapping of high-resolution driver elements, and would not be possible with regular STARR-seq unless extremely complex fragment libraries are used (at least 15 or 20-fold more complex than this current library). HDRA can therefore be feasibly and readily applied across a wide range of tissues for high-resolution mapping without the limitations that regular STARR-seq would entail (e.g. >10^9 cells per tissue, and high depth of sequencing to quantify a library with ~20-fold more fragments)."

For STARR-seq in human cells, there is an apparently unresolved methodological issue. In Drosophila, there seem to be two classes of enhancers ("developmental" and "housekeeping", Zabidi et al Nature 2015) which are revealed by using different promoter types in the reporter construct. In human, those two classes have not been resolved because the human STARR-seq plasmid prefers to use the CpG-rich bacterial ORI sequence as promoter instead of researcher-chosen ones (https://www.biorxiv.org/content/early/2017/07/17/164590). The authors of this paper do not comment on possible consequences of this for the results presented in this paper and their interpretation.

We thank the reviewer for raising this point. In the existing manuscript, we discussed the recent paper by Muerdter *et al.* and in particular how using a backbone without the minimal SCP1 promoter (i.e. relying only on the bacterial ORI sequence recommended by Muerdter *et al.*) is of value for future studies, specifically for improving the signal-to-noise ratio. We copy the text from the manuscript below:

> "Several improvements are possible on the existing HDRA experimental approach. In addition to the minimal SCP1 promoter, the STARR-seq vector has a second active promoter at the bacterial ORI site, whose transcripts are both more abundant and have stronger signal-to-noise ratios at regulatory elements[25]. Thus, to improve detection of modestly-active enhancer elements, future studies may forgo the SCP1 promoter altogether and utilize the ORI site exclusively." (lines 406-410)

The reviewer also raises an interesting point regarding Zabidi *et al.* that we believe is separate from the technical aspects of the Muerdter *et al.* paper. Specifically, Zabidi *et al.* observed that different promoters in *Drosophila* STARR-seq experiments can discover different sets of enhancer elements, including "developmental" and "housekeeping" enhancers. Assuming this division of enhancer sub-classes also exists in mammals, we believe the original human STARR-seq plasmid (which we use in our manuscript) would in fact be preferable to the new plasmid recommended by Muerdter *et al.*. As Muerdter *et al.* show (e.g. Fig. 1a of their paper), 27% and 59% of transcripts are produced from the SCP1 and ORI promoters in the original human STARR-seq plasmid, respectively. In contrast, removal of SCP1 (in the new plasmid) leads to all transcripts being produced from the CpG-rich ORI promoter. Thus, the diversity of promoter usage in our study should in theory increase the set of enhancers that can be discovered, as shown by Zabidi *et al.*.

We also strongly agree with the reviewer regarding the broader point raised by Zabidi *et al.*, where it is important for future studies to perform HDRA experiments using a variety of different promoters to increase the detection ability of enhancer sub-classes that may appear invisible to assays using a single promoter sequence. To highlight this to future readers, we have added a statement to the Discussion discussing this:

> "Zabidi et al. demonstrated that different promoters in Drosophila STARR-seq experiments uncovers different classes of enhancer elements (e.g. developmental vs. housekeeping). Based on Muerdter *et al.*, the pSTARR-seq_human plasmid employed here drives transcriptional initiation from two promoter elements: the SCP1 synthetic core promoter and the bacterial plasmid origin of replication, suggesting that the original pSTARR-seq_human plasmid may identify more active enhancer elements than newer STARR-seq plasmid backbones lacking SCP1. Future HDRA studies can also increase the diversity of promoter sequences tested to improve the detection of subclasses of enhancer elements." (lines 410-417)

- The principal difference with respect to the original STARR-seq protocol, which assayed the entire Drosophila genome in an unbiased manner, is the pre-selection of most likely regulatory sequences using ATAC-seq. This idea itself is not particularly novel - Vockley et al (Cell 2015) described an approach they called ChIP STARR-seq in which they performed STARR-seq assay on regions enriched for glucocorticoid receptor binding.

We thank the reviewer for this comment and the opportunity to highlight the differences between our study and Vockley *et al.*. There are three critical differences:

1. Most importantly, Vockley *et al.* does not achieve high-throughput dissection of regulatory elements. The ability to perform genome-wide high-resolution mapping of driver elements is the key advance presented by our manuscript. We believe this improvement in resolution we present (from peak-level to almost single nucleotide-level) represents a significant advancement that will influence all subsequent high-throughput enhancer reporter studies that prepare reporter libraries using capture or enrichment-based approaches.
2. ChIP-based capture of input fragments is limited to one or a few transcription factors at a time. As any tissue or cell line expresses hundreds of distinct transcription factors (many without high-quality antibodies), the ChIP-STARR-seq approach is best reserved for a specific biological hypothesis (e.g. the response of the gluticorticoid receptor to dexamethasone, as investigated by Vockley *et al.*), rather than for the global mapping of regulatory elements that we perform in our manuscript.
3. Theoretically, ChIP-STARR-seq could be used with H3K27ac or H3K4me1 to allow for genome-wide mapping of regulatory elements, although no-one has demonstrated this. However, we do not believe this allow for high-throughput dissection of regulatory elements, as the histone marks reside (by definition) on nucleosome-marked segments, rather than in the inter-nucleosomal regions where TF binding sites reside.

We sought to emphasize these differences throughout our manuscript, referring to ChIP-STARR-seq or the general STARR-seq method in following passages:

1) (*existing manuscript*) "Genome-fragmentation approaches (e.g. STARR-seq[16], Cap-STARR-seq[17,18], ChIP-STARR[19]) use DNA fragments collected or captured from genomic DNA." (lines 21-22)
2) (*existing manuscript*) "ChIP-based capture is limited in only one or few transcription factors at a time". (lines 27-28)
3) (*existing manuscript*) "Finally, the SHARPR2 high-resolution mapping algorithm developed here can be applied to perform high-resolution mapping of genomic regions for high-complexity libraries with sufficient fragment density (e.g. testing of individual large regions using bacterial artificial chromosome clones with "BAC-STARR-seq"[16], or high-resolution mapping of transcription factor binding sites with ChIP-STARR-seq[19])." (lines 442-445)
4) (*newly added*) A discussion of the differences between HDRA and STARR-seq, including highlighting the key ability to perform high-resolution dissection of regulatory elements (lines 382-392)

- The ATAC-seq library is dominated by the fragments that overlap predicted promoter sequences, and many of them are among the fragments giving the strongest signal enrichment in the final HiDRA signal. According to the default interpretation of the STARR-seq assay, not questioned by the authors, this would mean that promoter sequences can act as enhancers for the reporter gene promoter when placed in the gene's 3' UTR. This would be an interesting observation, which has not been convincingly demonstrated before. However, since no full-length reporter transcripts are sequenced, how can the authors be sure that placing a promoter-containing fragment in 3' UTR does not results in its - promoter activity? In other words, can the authors exclude the possibility that this results in short non-coding transcripts that start at the cloned promoter (instead of at the reporter gene promoter) and extend into the part of the reporter gene 3' of the cloning site (which is the part targeted by primer for reverse transcription)?

When designing the cloning strategy and primer sequences, we did consider this possibility, but concluded that transcription from an internal TSS present on the inserted fragment will not impact the HDRA readings.

Specifically, transcripts generated from an internal TSS will lack a necessary PCR primer site and will not be PCR amplified and therefore cannot be sequenced. The diagram below visually illustrates this by showing fragments transcribed from the normal promoter ("Transcription from upstream TSS") vs. those from an internal 3' UTR promoter ("Transcription from internal TSS"):

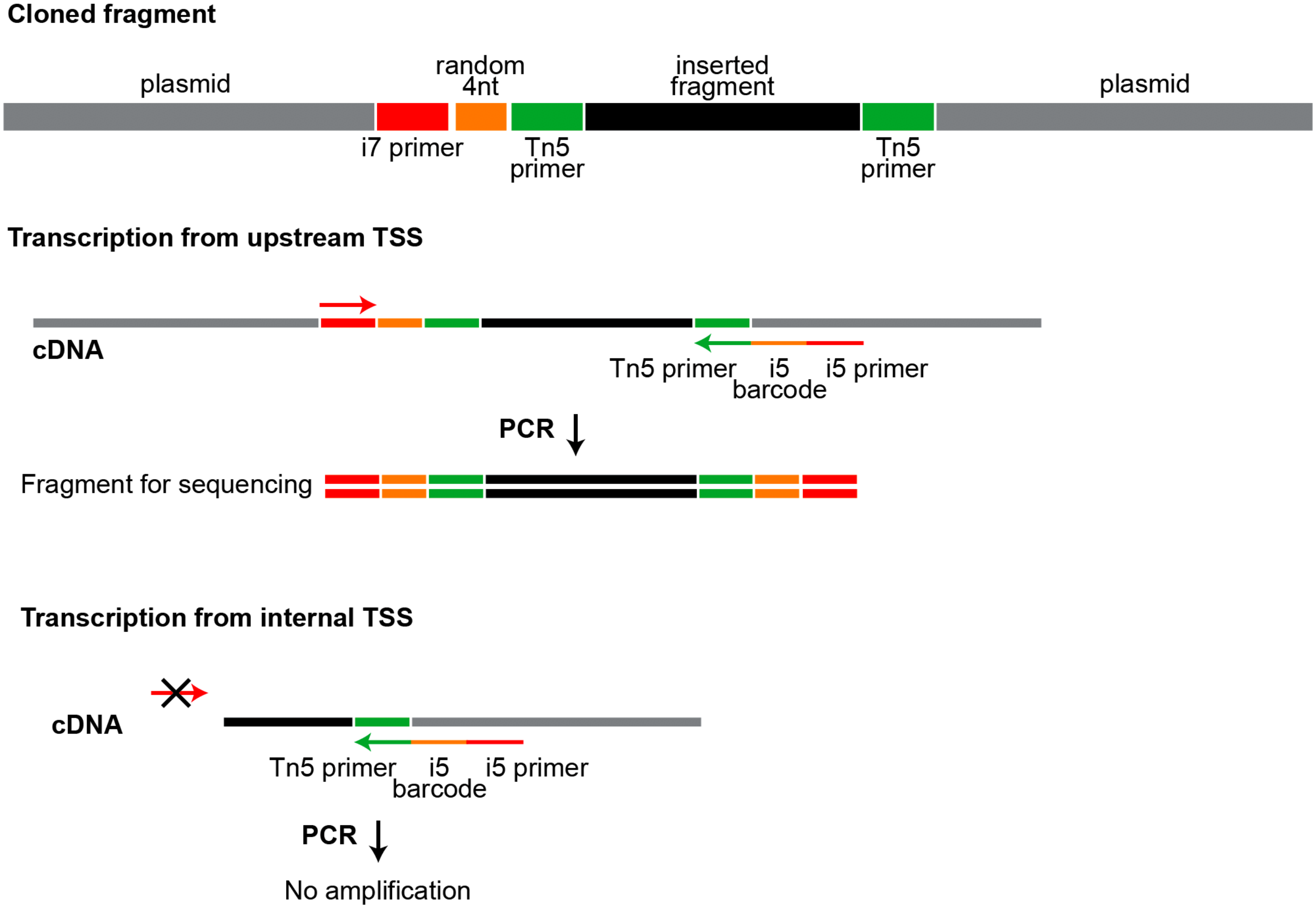

- In Figure 4, the most active region seems to be the least accessible. Is it because the transcription factor binding shields the sequence from the transposes, or because the DNA input is enriched for mononucleosome-length fragments?

The reviewer makes an important observation, namely that chromatin accessibility by ATAC-seq does not necessarily correlate with regulatory activity. We also agree with the reviewer that the "dip" likely corresponds to previously reported "TF footprints" that have been applied to identify TF binding sites (e.g. PMIDs 19305407, 25409825 and 25297728).

- The motif and GWAS signal analysis give unsurprising results compared what the analysis of ATAC-seq regions themselves would yield, given what is known about the GM12878 cells.

We agree with the reviewer about the importance of using the correct background to calculate TF motif enrichments. As we discuss in the text, the background set we use for both analyses is the set of all "tiled regions". These are the ~32,000 regions that we used as the input to the SHARPR2 algorithm for driver element discovery. To assess significance of signals (e.g. for motif & GWAS analysis), we shuffled the position of the driver elements within these tiled regions, and not within the entire genome. We mention in the text that this background set is used, and that it is highly enriched for GM12878 regulatory regions:

"Compared to a background of all tiled regions, which are specifically enriched for GM12878

regulatory regions, we found that predicted driver nucleotides were significantly more enriched for regulatory motifs than shuffled controls (obtained by randomly shuffling driver element positions within the same set of tiled regions with at least 10 unique HDRA fragments)." (lines 258-261)

and

"Even though driver nucleotides only cover 0.032% of the genome, we found 12 cases where they overlap fine-mapped SNPs associated with 21 immune-related traits[31] predicted to be causal (~5 expected by chance inside tiled regions, p=0.012 vs. random shuffling, Fig. 5a)." (lines 303-306)

- (cca lines 93-100 in the manuscript) Is the comparison of RNA signal with non-transfected DNA input library the right thing to do? Transfection efficiency can be significantly different for different DNA fragments, depending on both their size and nucleotide composition. Many human promoters are very GC and CpG rich, unlike the majority of enhancers and a minority of tissue-specific promoters. This may introduce bias if untransfected library is used as baseline. If it is not possible to sequence the transfected DNA library, some proxy checks should be required (e.g. whether longer fragments or more GC rich fragments give systematically higher RNA signal).

The reviewer raises an important point regarding the proper control to use for identifying active fragments. Our choice of comparing against the non-transfected DNA library was based on past studies, such as the STARR-seq manuscript (Arnold *et al.* 2012, Science), that report no difference between transfected & untransfected libraries.

To further guard against possible length-dependent biases, we split our input set by different length bins and had called active fragments in each bin separately. We provide text and a figure from the manuscript describing this, and showing that the median log(RNA/DNA) ratio is unchanged across different fragment lengths:

"As we compare RNA signal to the non-transfected DNA library, we controlled for the possible length-dependent biases in transfection efficiency of plasmids by splitting fragment groups into separate bins of 100nt (100-200nt, 200-300nt, etc.) and used DEseq2 (v1.10.1) to identify FDR<0.05 significantly up-regulated fragment groups in each bin." (Methods section)

Additionally, in the supplemental information, we show that there is no bias in activity by read length and reproduce this below:

"The average and the variance of the signals are constant across the length of HiDRA fragments after normalization (Materials and Methods, Supplemental Figure S12)."

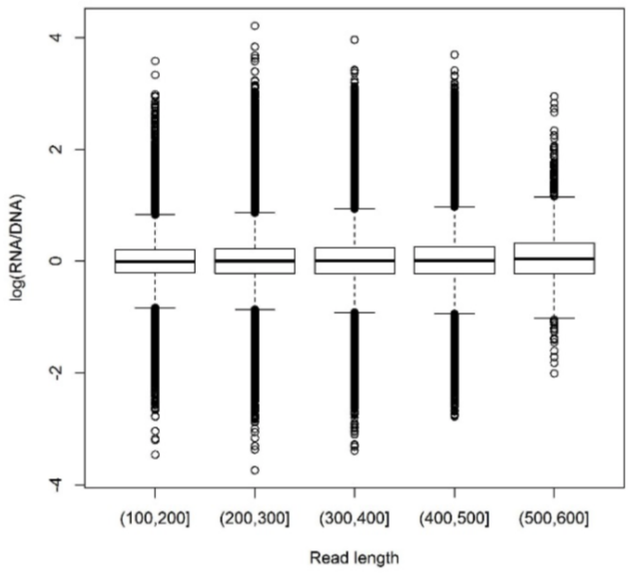

Supplemental Figure S12: log(RNA/DNA) ratio vs. fragment read length

Regarding GC content, we do not believe that the GC content of the fragment will substantially affect transfection efficiency, as the size of the inserted fragment is much smaller than the STARR-seq backbone (which is constant between fragments). The STARR-seq backbone we use is ~3.5kb in length with approximately ~50% GC content. Therefore, the difference in total GC content between a plasmid carrying a 500nt fragment with 30% GC content, and a 500nt fragment with 70% GC content would therefore be only 3% ([3500*0.5 + 500*0.3]/4000 vs. [3500*0.5 + 500*0.7]/4000). Most differences in GC will not be so extreme, and we therefore do not believe GC content will meaningfully impact transfection efficiency.

Additionally, as the reviewer notes, different classes of genomic element have varying GC contents (e.g. promoters generally have high GC). Thus, we believe that a systematic analysis of GC content against reporter activity to assess GC-dependent transfection biases would be uninformative, as the analysis would be heavily confounded by both the genomic origin of the fragment, as well as transcription factor motif composition, which is also dependent on fragment GC content.

- While the correlation of HiDRA signal with the luciferase assays of known enhancers is strong, the fact that heterochromatin regions are modestly enriched in HiDRA signal and that e.g. Polycomb-repressed regions are depleted means two things: first, that some fragments in the plasmid are more accessible/exposed than in their native chromatin context. This is not unexpected, but does lead to false positives if the signal is taken as a measure of what actually happens in the genome. Secondly, and for me more interesting, some DNA fragments, such as Polycomb-repressed fragments, apparently contain enough information to attract Polycomb repression even in the plasmid; I cannot see other reason why they should be further depleted relative to input. These issues should be commented on in the paper.

The reviewer raises an interesting and, to our knowledge, unresolved point regarding whether episomal plasmid constructs have chromatin modifications. Bintu et al. (Science 2016, PMID 26912859) demonstrated that an episomal “human artificial chromosome” (HAC) vector is capable of having chromatin modifications, however HACs are often 2-10Mb in length, approximately 1000X larger than the plasmids used in this study.

Regarding the depletion of Polycomb-repressed & Quiescent regions in fragments with enhancer activity, we believe this is largely a consequence of enrichment of active fragments for other regions (e.g. enhancers, promoters). If some classes of regions are over-represented in active regions, then the proportion of regions without activity (e.g. Polycomb-repressed fragments) will be depleted as a consequence. We mention this explanation briefly in the results section of our manuscript:

> “In contrast, Quiescent and Polycomb-repressed chromatin states showed a 2-fold relative depletion for HDRA active elements, accounting for the enrichments found in other states.”

(lines 157-159)

As we discuss in the text, the discrepancy between heterochromatin (no depletion in high activity fragments) and Polycomb-repressed & Quiescent regions (strong depletion) suggests that heterochromatin may contain sequences that are normally repressed but become active outside of the native chromatin context. We believe this represents an intriguing area of further study. As recommended by the reviewer, we include a statement on the possibility of chromatin modifications on the plasmid:

> “The depletion of Polycomb-repressed chromatin states may reflect Polycomb repression on the episomal plasmid.” (lines 159-160)

Reviewer #3 (Remarks to the Author):

The manuscript by Wang et al. describe the use of an episomal reporter assays to directly identify regions from open chromatin that are capable of driving reporter gene expression. The authors accomplish by using the novel approach of inserting an ATAC-seq library, captured from a lymphoblastoid cell line, into a STARR-seq vector and testing activity using a slightly modified STARR protocol. This approach is advantageous due to the use of ATAC-seq as a starting material which can be more cost effective compared to using a whole genome or BAC based library or synthesized oligos. By selecting only ATAC-seq fragments the authors are enriching for elements thought to drive regulatory activity while maintaining a "whole-genome" approach. This allows a large number of elements to be evaluated by the assay providing a comprehensive survey of where episomal assays are capable of characterizing regulatory activity and where the assay underperforms. This knowledge, the strength and limitations of reporter assays, is lacking and dearly needed as the assay gains in popularity. Finally, the authors further extend their assay to recover heterozygote alleles allowing them to evaluate allelic differences in regulatory activity.

The paper is well written, the analysis is comprehensive and thoughtfully executed. The approach presented here is novel within the family STARR based assays and one that will likely represent a significant proportion of STARR-seq assays going forward. This work, the first to my knowledge that uses ATAC-seq, will serve as a valuable blueprint for future studies. Most of my comments are either clarifications that would help the flow of the paper or additional analysis I would be curious to see and would further strengthen an already strong study. I only have several concerns that I would consider critical.

Critical comments:

-A sticking point for me is the new name (HiDRA) the authors provide for a preexisting assay. I would strongly recommend the authors adhere to the conventional naming and describe their assay as "atac-STARR" or "ca-STARR" (chromatin accessible) coupled with an analysis using SHARPR2. An additional acronym would unnecessarily fragment the field and add confusion to those new to the field.

We appreciate the reviewer's comment regarding the name of our assay. As we explain below, we believe our high-resolution mapping assay is more similar in function to MPRA (developed for systematic dissection of regulatory elements) than STARR-seq (developed for discovery of regulatory elements). We have therefore changed the acronym of our assay from "HiDRA" to "HDRA" to emphasize its similarity to MPRA. This MPRA-style naming scheme follows other high-throughput reporter assays, including the GPRA (Gigantic Parallel Reporter Assay, doi.org/10.1101/224907).

Detailed explanation:

HDRA falls between two families of methods seeking to profile regulatory elements: MPRA-based methods, and STARR-seq based methods.

- STARR-seq was developed for **discovering** enhancers *de novo*. In the initial paper describing STARR-seq, the authors compare STARR-seq to ChIP-seq and DHS-seq, two other methods used for the systematic discovery of putative regulatory elements (e.g. “Here, we present STARR-seq, which complements ChIP-seq and DHS-seq as the third principal method to study transcriptional regulatory elements in entire genomes.”, Arnold et al, Science 2013).
- By contrast, MPRA-seq methods were developed for the “**systematic dissection**” and “**functional analysis**” of **candidate** enhancer regions, which were predicted by other methods (Melnikov *et al.* Nature Biotechnology 2012).

Between these two families of methods, we believe that HDRA aligns closely with MPRA-based methods, rather than the STARR-seq family, even though we use the STARR-seq vector to test the activity of DNA fragments. HDRA is the only method, along with Sharpr-MPRA, that allows high-throughput dissection of putative regulatory regions in a massively parallel high-throughput way. For this reason, we named our assay the “High-Definition Reporter Assay” (HDRA) to highlight its similarity to the “Massively Parallel Reporter Assay” (MPRA). In our manuscript, we have tried to give credit in our paper to both STARR-seq-based methods (e.g. lines 21-22, 25-28, 60, 382-392, 442-445) and MPRA-based methods (e.g. lines 17-25, 230-232, 372-381), rather than focusing solely on STARR-seq, as both the motivation and the accomplishments of our method stems on both families of methods.

In our revised manuscript, we add two additional paragraphs in the discussion that (i) highlight the point that HDRA is more conceptually aligned with MPRA, and (ii) highlight the differences between HDRA and STARR-seq. We copy the two paragraphs below:

> (Comparison vs. MPRA, lines 372-381)
> "Currently, the only other method that enables the high-resolution dissection of thousands of putative regulatory regions is Sharpr-MPRA[13]. Both Sharpr-MPRA and HDRA seek to map regulatory regions at high resolution, but they differ in key several respects: Sharpr-MPRA selects regions based on prior computational predictions, requiring microarray-based synthesis. This *in vitro* synthesis step limits the number of nucleotides and regions tested by Sharpr-MPRA (4.6Mb in 15,720 regions for Sharpr-MPRA vs. 46.3Mb in 31,813 regions for HDRA). Moreover, Sharpr-MPRA uses fixed 5-bp increments (vs. random increments for HDRA, stemming from the transposase fragmentation pattern), 145-bp fragments (vs. random lengths for HDRA, 335-bp on average), and Sharpr-MPRA tests elements upstream of the TSS using 3'UTR barcodes (vs. testing elements downstream, thus enabling them to serve as their own barcodes)."
>
> (Comparison vs. STARR-seq, lines 382-392)
> "The HDRA approach has many benefits compared to regular STARR-seq. HDRA preferentially tests the activity of fragments derived from open chromatin, and the HDRA library we developed achieves 130-220X more highly covered regulatory elements than random genome fragmentation (**Fig. 1c**). This facilitates both higher confidence mapping of regulatory elements, which will have a greater number of unique fragments, and facilitates the high-resolution mapping of regulatory elements by comparing the relative activity of partially-overlapping fragments. This latter application using our newly developed SHARPR2 algorithm permits the genome-wide mapping of high-resolution driver elements, and would not be possible with regular STARR-seq unless extremely complex fragment libraries are used (at least 15 or 20-fold more complex than this current library). HDRA can therefore be feasibly and readily applied across a wide range of tissues for high-resolution mapping without the limitations that regular STARR-seq would entail (e.g. $>10^9$ cells per tissue, and high depth of sequencing to quantify a library with ~20-fold more fragments)."

-Lines 63-66: The authors describe pricing differences between capture-based reporter assays (STARR-seq) and methods that use oligo synthesis as the input source. In its current form, the comparison only accounts for library preparation and not the entire experiment. To perform a proper comparison, one needs to consider both sequencing costs (paired and single-end) as well as uniformity of the libraries which impacts the sequencing depth required for adequate coverage. Price comparisons are always tricky; I would recommend removing it completely, significant simplification of the statement or provide a detailed breakdown of the budget in the supplemental which includes labor, sequencing and all reagent costs.

We thank the reviewer for this comment and reasoning, and we have removed the statement on cost from the text.

-Lines 250-255: Specific claims are made regarding the ability to map regions down to the individual nucleotides responsible for activity. There are several things I would like to see to support the observations and claims by the authors. (i) It would be nice to have either an MPRA (using synthesized oligos) or individual luciferase assays of several regions with and without the active regions identified by the SHARPR2. This will demonstrate how well SHARPR2 can pinpoint the active region and more importantly the proportion of signal that region contributes to the overall activity. In other words, are the SHARPR regions responsible for all of the signal or contribute in an additive (or non-additive) manner in conjuncture with additional undetected regions? Without this it is difficult to make the claim on line 222-223 that driver elements are slightly larger than regulatory motifs. It is unclear if this is a biological observation or a limitation of the assay and analysis.

We thank both Reviewer 1 and Reviewer 3 for suggesting additional experimental validation for our driver element predictions using perturbation assays. We now include such experimental validation of reporter assays with the sequence of the 'driver' region shuffled, and we show the results strongly support our conclusions (see point #7 below). We used MPRA-based validation, as recommended by Reviewer 3, as this more directly matches the experimental procedures utilized here.

In addition to these shuffled nucleotide perturbations, we also evaluate the effect of single-nucleotide genetic perturbations in our predicted driver regions (point #4 below), and we compare our driver region inferences with activity scores from the Sharpr-MPRA study. In total, now present seven lines of evidence to support the functional relevance of driver elements, including data from three separate MPRA experiments. We present these below:

8) (*present in original submission*) Driver elements are enriched for relevant motifs corresponding to TFs active in GM12878, compared to a background set of all tiled regions (used as input to SHARPR2 to discover the driver elements). This was originally presented as **Fig. 4c** and **4d** and is copied below.

9) (*present in original submission*) Driver elements are significantly more likely to be evolutionarily conserved compared to randomly selected elements of equal size within tiled regions used as input to SHARPR2 to discover the driver elements. This indicates that driver elements are significantly more likely to be functionally important and therefore under purifying natural selection. This was originally presented as **Fig. 4e** and is copied below.

10) (*present in original submission*) Driver elements are significantly more likely to overlap fine-mapped GWAS SNPs associated with autoimmune diseases, compared to randomly selected elements of equal size within tiled regions. This indicates that genetic variants within driver elements are more likely to be functionally relevant and therefore be associated with autoimmune disease risk. This was originally presented as **Fig. 5a** and **5b** and is copied below.

11) (*newly added as Fig. 4f*) Consistent with our results showing that driver elements are enriched for fine-mapped autoimmune SNPs (Fig. 5a), we observe that SNPs overlapping driver elements exhibit a significantly stronger allelic skew by MPRA (difference in MPRA reporter activity between the two alleles) compared to two background sets: SNPs in tiled regions used to discover the driver elements, and the entire set of SNPs tested from the genome. This indicates that SNPs within driver regions are more likely to disrupt enhancer activity than those outside drivers, indicating that driver elements are important for enhancer activity.

12) (*newly added as Supplemental Fig. S11a*) We shifted the position of driver elements both upstream and downstream, and we find that these adjacent regions have weaker evolutionary conservation than true driver elements. This suggests driver elements are positioned precisely at regions of functional importance that are under purifying natural selection.

13) (*newly added as Supplemental Fig. S11b*) We compared driver elements identified in our study with enhancer functional importance scores calculated by Ernst *et al.* 2016 (Nature Biotechnology). Ernst (2016) presented the original Sharpr-MPRA algorithm for high-resolution mapping of regulatory nucleotides, and comparing against this study offers an independent validation of our driver elements using an MPRA-based platform. We observe:
    a) Driver elements from our study have higher Sharpr-MPRA activity scores compared to adjacent regions. This agrees with corresponding evolutionary conservation analyses, and indicates that driver elements are positioned at the region of functional importance
    b) This enrichment of Sharpr-MPRA activity is observed in comparisons with both cell types tested by Ernst *et al.* (2016): K562 and HepG2
    c) Consistent with similarity between cell types, our driver elements (identified in GM12878 lymphoblastoid cells) have higher activity in K562 (chronic myelogenous leukemia line) than HepG2 (hepatocellular carcinoma line)
    d) Only HDRA driver elements that carry a TF motif active in either K562 or HepG2 show activity in the corresponding cell type, providing a mechanistic explanation for the similarities in activity.

14) (*newly added as Supplemental Fig. S11c*) We predict that disrupting the nucleotide sequence in driver elements will reduce or eliminate enhancer activity. Kheradpour *et al.* (2013) used MPRA to randomly shuffle the sequence of a set of regulatory motifs, and tested activity of wild-type and shuffled sequences in both K562 and HepG2. Of Kheradpour et al.'s tested elements, four showed activity in either K562 or HepG2 and also overlapped driver elements predicted in our study. Disrupting motifs all

four driver elements led to significant decreases in enhancer activity in either K562, HepG2 or both cell lines. This result indicates that experimentally disrupting a driver element leads to a reduction or complete loss of enhancer activity.

Figure panels corresponding to these seven lines of evidence are presented in order below:

Figure for evidence #1 (present in original submission as Figs. 4c and 4d):

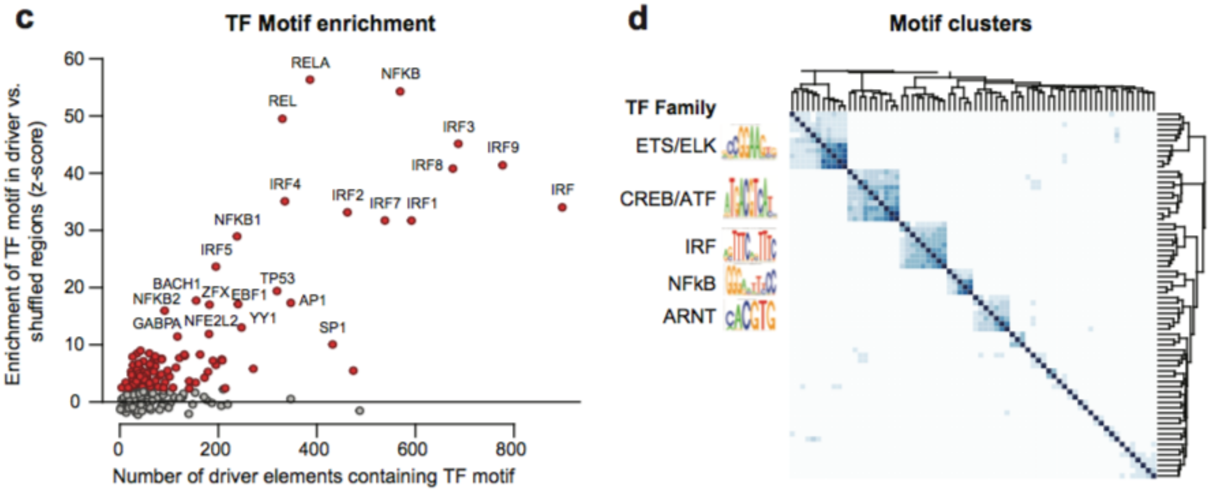

Figure for evidence #2 (present in original submission as Fig. 4e):

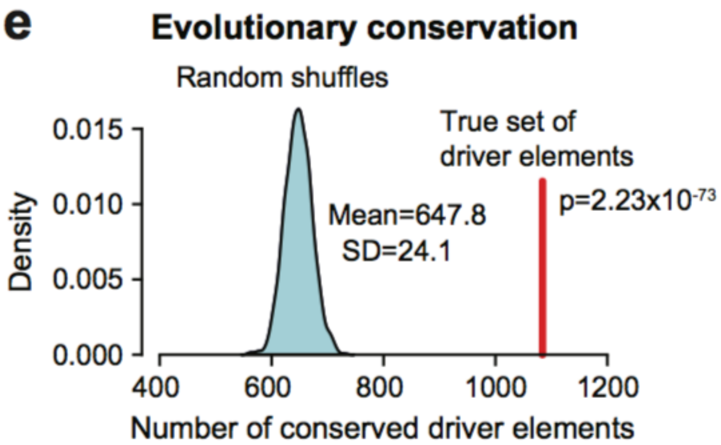

Figure for evidence #3 (present in original submission as Fig. 5a):

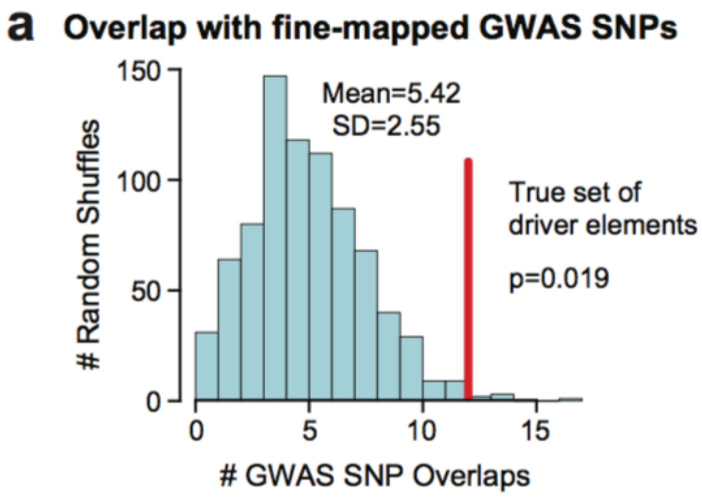

Figure for evidence #4 (newly added as Fig. 4f):

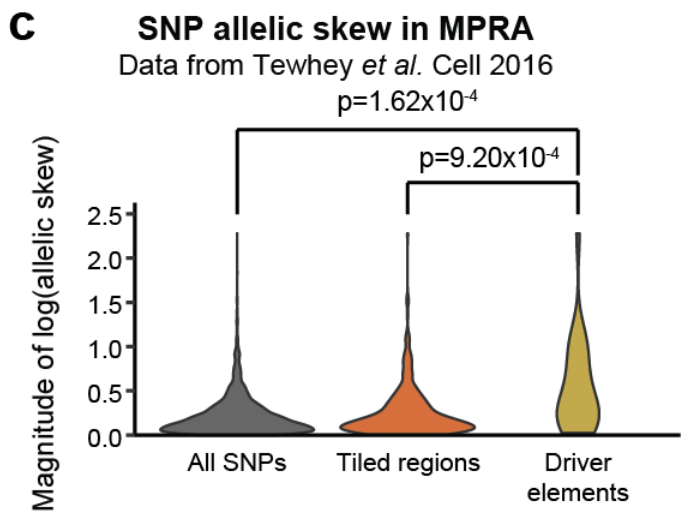

Figure for evidence #5 (newly added as Fig. S11a):

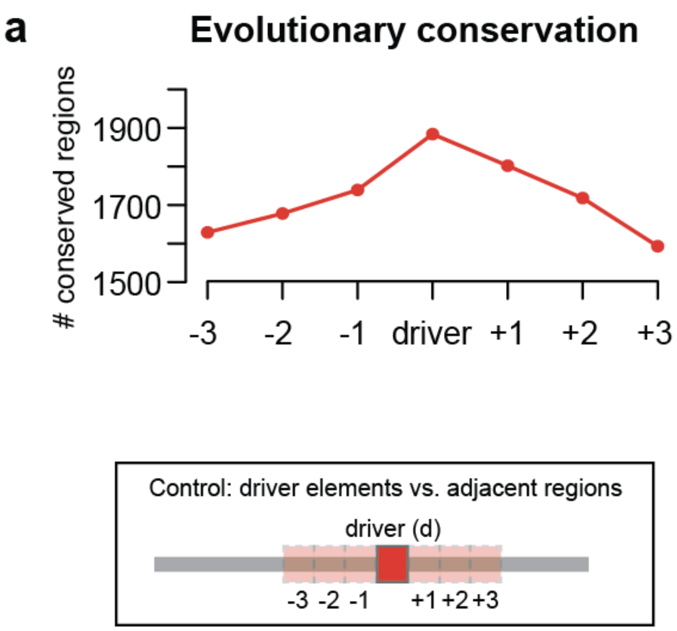

Figure for evidence #6 (newly added as Fig. S11b):

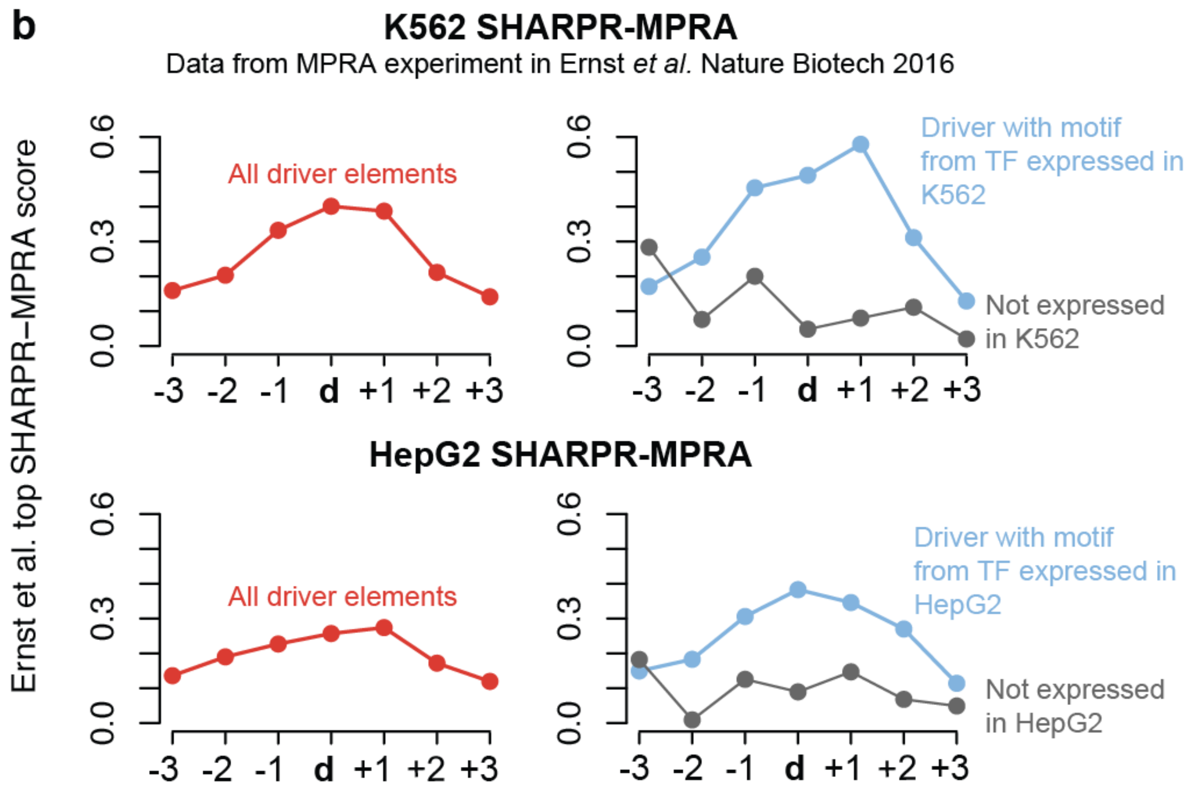

Figure for evidence #7 (newly added as Fig. S11c): Values in cells correspond to activity of MPRA element containing true sequence vs. MPRA element containing shuffled sequence, followed by p-value of the difference across replicates.

c

Shuffled elements by MPRA

Data from MPRA experiment in Kheradpour *et al.* Genome Research 2013

Driver element (nt)	MPRA shuffled element (nt)	Mutual overlap	K562 median scores (p-value)	HepG2 median scores (p-value)
chr12:76371849-76371904 (56nt)	chr12:76371893-76371906 (14nt)	12nt	1.59 vs. 1.39 (p=0.62)	4.22 vs. 1.66 ($p=1.48x10^{-9}$)
chr5:149185564-149185589 (26nt)	chr5:149185566-149185576 (11nt)	11nt	0.79 vs. -0.32 ($p=8.70x10^{-3}$)	1.44 vs. 0.58 ($p=1.32x10^{-4}$)
chr5:54040333-54040401 (69nt)	chr5:54040375-54040385 (11nt)	11nt	1.35 vs. 0.75 (p=0.029)	1.21 vs. 0.32 (p=3.40x10-3)
chr11:48028283-48028351 (69nt)	chr11:48028311-48028328 (17nt)	17nt	1.27 vs. -0.09 ($p=1.71x10^{-3}$)	Not active in HepG2

Finally, regarding the reviewer's point on driver element size vs. motif size, as we did not perform the additional experiments to explicitly test the 18nt driver size (compared to 5, 10, 15nt), we have modified the manuscript text to also mention that the 18nt size may be due to properties of the assay:

> "Regions tiled by fewer fragments (10-20) showed lower resolution (~50nt), but resolution only increased to ~18nt with higher fragment density, suggesting the minimum size of driver elements detectable by the HDRA assay that result in regulatory activity is only slightly longer than individual regulatory motifs." (lines 252-254)

Minor comments and extensions:

-How was the library size of 9.7M estimated? In addition, it would also be helpful to have table in the supplemental describing how many ATAC-seq reads were lost for each filter (e.g. mapping score, mitochondria, etc).

The 9.7M library size corresponds to the number of unique fragments defined by genomic position (chromosome, start and end coordinates of the fragment) after paired-end sequencing and filtering steps. As recommended by the reviewer, we also include a flow chart showing the percentage of reads lost to each filter. The largest loss occurs when removing mtDNA fragments, which is a well-known issue with ATAC-seq (e.g. PMIDs 24097267, 28550296), and the reason we included a mtDNA-CRISPR treatment step.

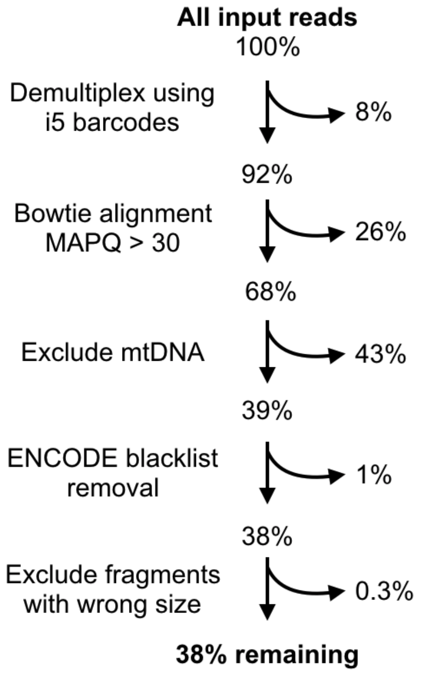

Supplemental Fig. S13: Proportion of reads lost by each processing filter for the HDRA library.

-Figure 1b, it is unclear if this figure is displaying the library from Buenrostro et al. or the HiDRA library. If it is the prior it should be changed to show the fragment distribution for the HiDRA library.

We apologize for the confusion - Figure 1b refers to the HDRA library, and we have added a heading to this panel to clarify this.

-Figure 1c, if space permits in the figure legend please briefly state where the enhancer and TSS states are taken from (chromHMM?).

We now specify in the legend of Fig. 1c that both the Active enhancer and Active TSS states are defined by ChromHMM.

-Paragraph at line 145: I found the observation of higher activity of enhancers relative to promoters to be interesting and one that could be unpacked a bit more in the paper. Instead of looking at enrichment of TF sites (Fig S4b) are there specific TFs that contribute to the higher activity observed in enhancers or is there a greater density of TFs? It would seem that you have enough data here to tease this observation apart.

We looked into this and observed that highly active fragments (top 25% RNA/DNA ratio) in both active TSS and enhancer regions are enriched for a variety of TF motifs (including NF-KB) compared to lowly expressed active fragments (bottom 25% RNA/DNA ratio, Supplemental **Fig . S4f**). Surprisingly, the highly active fragments do not have a greater density of TFs (Supplemental **Fig. S4e**). We counted the total number of nucleotides that fell within an annotated motif, and observed no difference between active TSS elements with high and low activity (p=0.51, Mann-Whitney U test). For enhancer elements, the most highly active elements had significantly fewer motif nucleotides than lowly active elements (21.3% vs. 24.6% of nucleotides, $p=4.21 \times 10^{-7}$, Mann-Whitney U test). Our results suggest that motif composition rather than number contribute to activity.

e

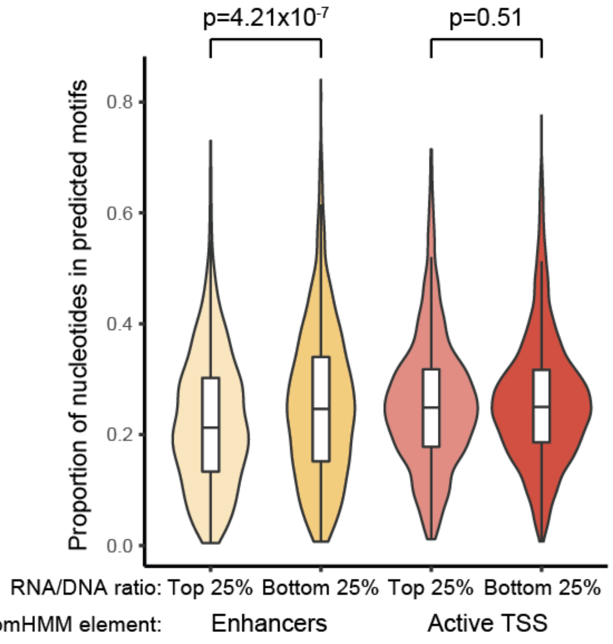

f

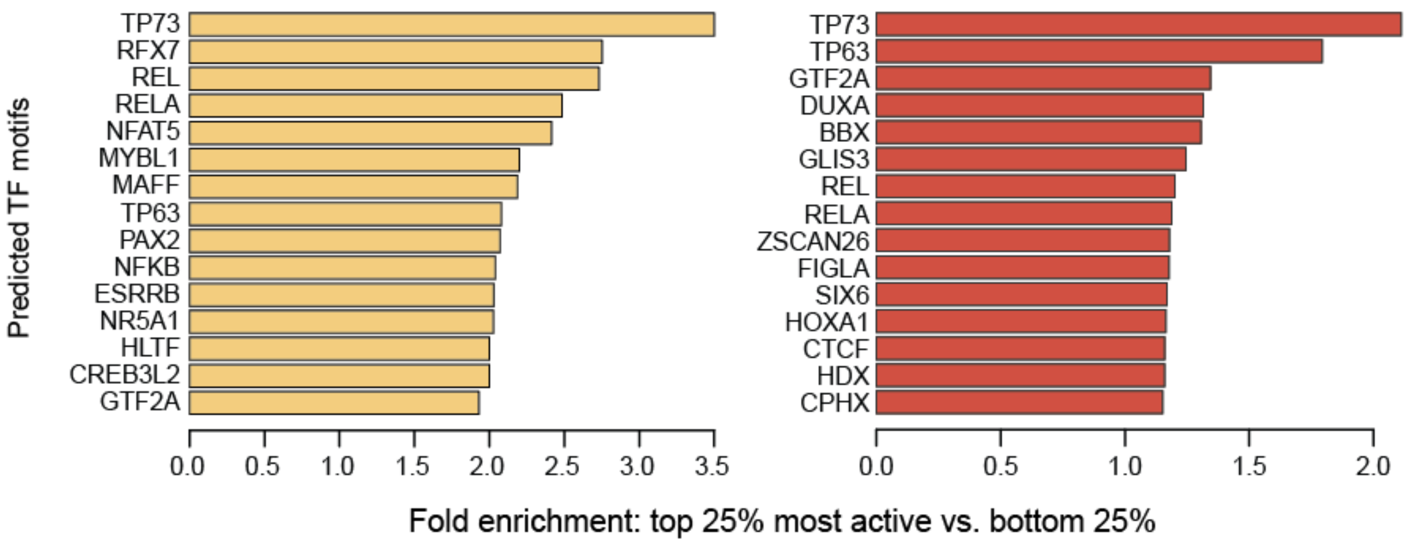

Supplemental Figure S4: (e) Proportion of nucleotides inside motifs for highly and lowly active fragments in enhancers and active TSS regions. **(f)** Enriched motifs in highly active enhancer (left) and active TSS (right) regions.

-Line 175: Can you provide a supplemental figure describing the lack of overlap of enriched TFs found in active elements annotated as enhancers and promoters? What elements are enhancer and promoter specific?

We thank the reviewer for this suggestion. We identified motifs most enriched in enhancers and active TSS state regions and added this to Supplemental Figure S4. The major motif group enriched in enhancers, but not promoters, are NF-KB-family motifs (**Supplemental Fig. S4c** below). We also include a Venn diagram comparing the top 50 motif enrichments between the two states, finding that the majority of enriched motifs are not overlapping (**Supplemental Fig. S4d**).

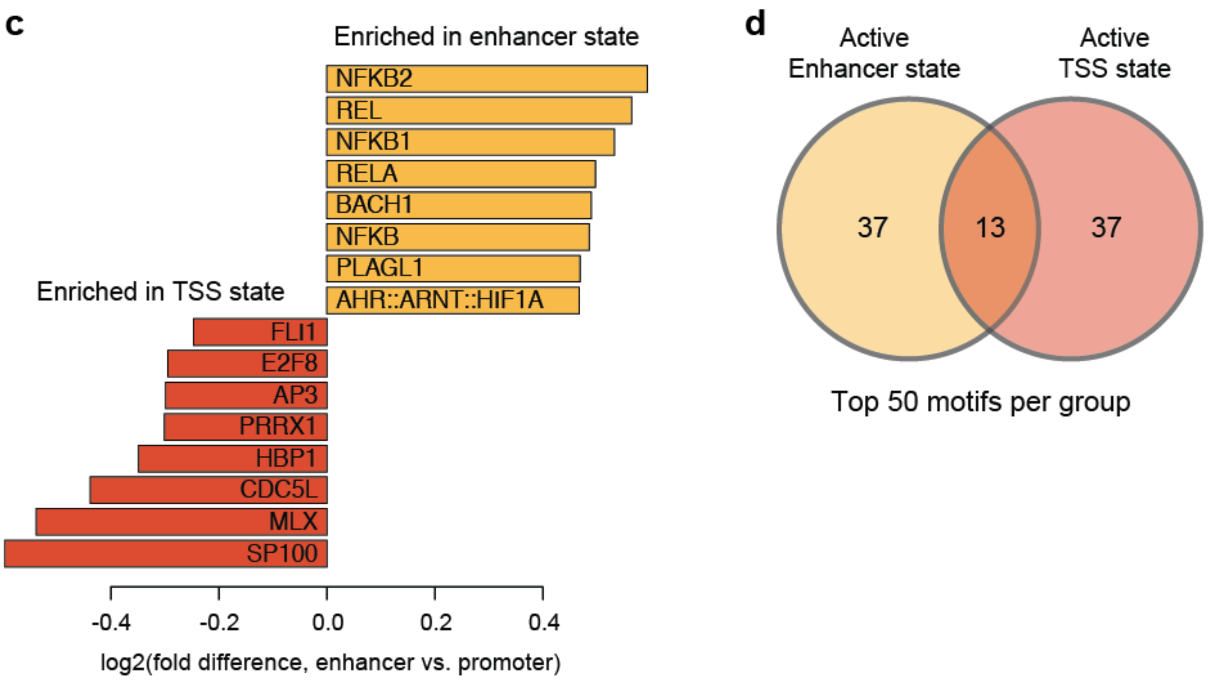

Supplemental Figure S4: (c) Top motifs that are differentially enriched in enhancer vs. active TSS states. (d) Top motifs enriched in enhancer and active TSS states are largely distinct from each other

-Paragraph at line 195: I would suggest carefully reviewing terminology in this paragraph. The use of in vivo at line 199 should probably read "in its chromosomal context". The use of occupancy in line 201 is suggestive of a physical interaction.

We thank the reviewer for this comment and have made the following changes to the text:

> "We reasoned that these regions would contain sequence motifs of TFs active in GM12878, but that these sequence motifs would be less likely to be bound in their endogenous chromatin context, compared to motifs in active states." (lines 199-201)

> "Indeed, we found that active HDRA regions from endogenously-inactive chromatin states showed similar enrichments in regulatory motif sequence coverage to that of enhancer and promoter chromatin states" (lines 201-203)

-Paragraph at line 206: Is there any difference in the LTRs between enhancer/promoter and the quiescent/heterochromatin regions that could explain the observed activity differences? For example, are the quiescent LTRs younger and thus less divergent?

To address this question, we focused on LTRs overlapping active regulatory elements, and calculated the proportion of these LTRs from each category present in genomes of eight other vertebrates: chimpanzee, macaque, dog, cow, mouse, rat and zebrafish. Most human LTRs in active regions are present in two other species (chimpanzee and macaque, see figure below). We observe no significant difference in LTR presence across species between activating chromatin states (enhancer & promoter) vs. quiescent/Polycomb-repressed states (p=0.22, Mann-Whitney U test). However, we do observe that activating LTRs from heterochromatin are slightly younger than either enhancer/promoter states or quiescent/Polycomb-repressed states ($p=2.0\times10^{-6}$ and $p=1.3\times10^{-7}$, respectively, Mann-Whitney U test).

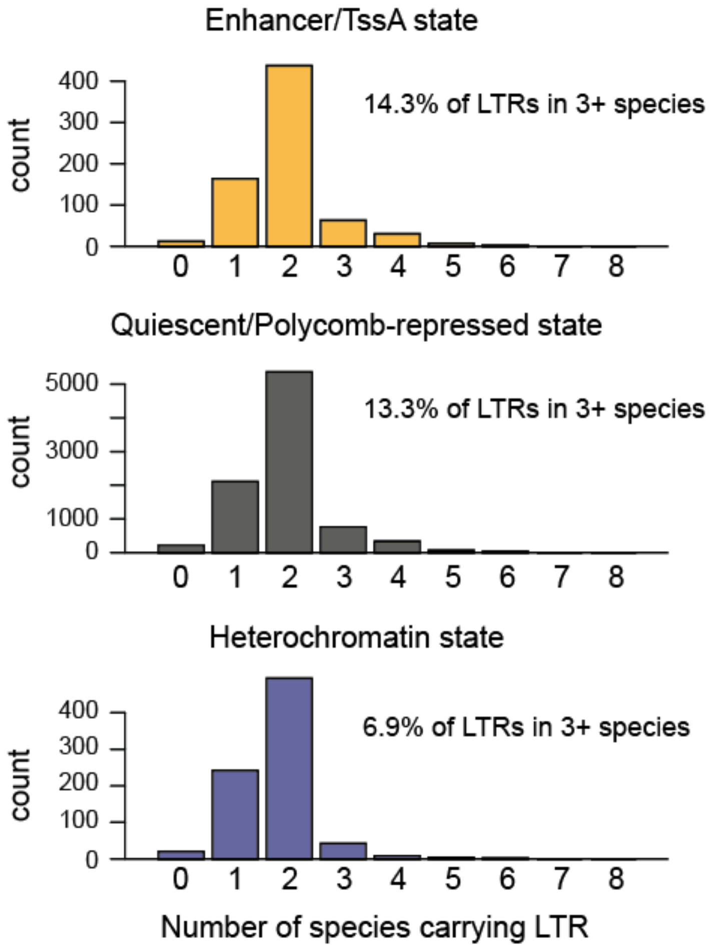

-For the driver elements identified by SHARPR2, where are they positioned within the ATAC-seq peaks? (e.g. Is there a trend for the active region to be centered at the summit of the peak)

We assessed the position of driver elements within tiled regions, and as the reviewer predicts, we observe that driver elements are more likely to be positioned near the center of the tiled region. We believe this is due to ATAC-seq's preference for creating fragments that are centered at inter-nucleosomal "dips", which are well-documented to be enriched for TF binding sites (e.g. PMIDs 19305407, 25409825 and 25297728), and thus will be enriched for driver elements.

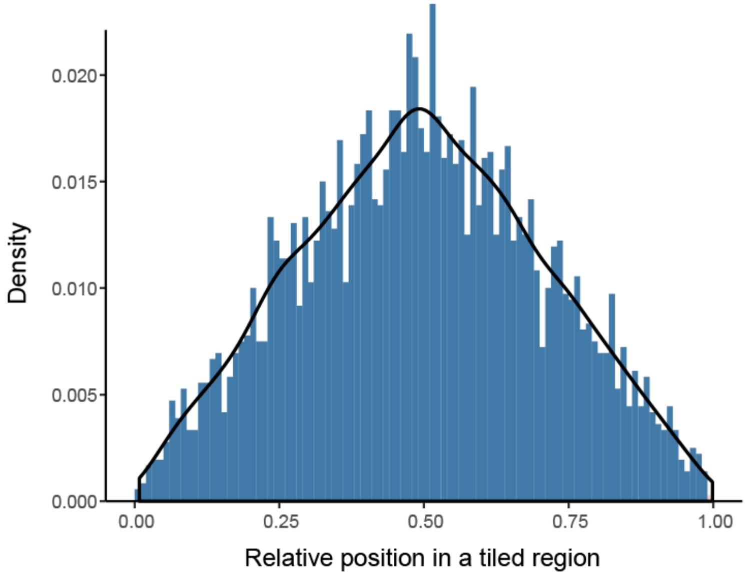

-Paragraph at line 300: This is a wonderful extension of the dataset but one I fear is underpowered for any general conclusions. What is the depth of coverage for the 16,000 filtered variants? There doesn't appear to be a coverage filter applied to this set. If you were to only take the alleles that were well powered to detect a 20-50% allelic skew how many 'allelic' SNPs are identified?

We thank the reviewer for his/her kind comments on this application, and we apologize for this oversight. In addition to other filters described (e.g. fragment ends away from driver elements), we require a minimum depth of coverage of 20 reads for both the reference and alternate alleles for every variant tested. We have added this statement to the manuscript. Regarding statistical power, we are not sure how to estimate this for the QuASAR-MPRA algorithm we used. Power calculations can be performed if allelic skew was detected assuming a binomial distribution, however many papers have shown that use of a binomial distribution is inappropriate and subject to high overdispersion, leading to high false positive rates.

The reviewer's general point on power is well-taken, and we agree that statistical power will be an issue for this extension of the HDRA assay. In the original manuscript, this section starts with a statement emphasizing the proof-of-concept and exploratory nature of this analysis:

> "In a proof-of-concept analysis to assess the ability of HDRA to detect allelic activity, ... " (line 330)

The discussion section in the original submission also comments on future improvements to the HDRA assay for detecting allelic activity, including to increase statistical power:

> "At the library construction stage, capture probes can be used to further increase coverage at known polymorphic SNPs of interest, thus increasing fine-mapping resolution, facilitating comparison of fragments with alternate alleles but matching start and end positions, and increasing statistical power to detect differential activity between risk and non-risk alleles." (lines 425-428).

In the revised manuscript, we have also changed the results section to more strongly emphasize the possible confounders and the issue with statistical power (which can be addressed with the experimental improvements listed above):

> "Detection of allelic activity with random fragmentation is subject to confounders, as fragments carrying the maternal or paternal allele of a SNP may also differ at their start and end positions, which may result in activity differences independent of SNP effects (Supplemental **Fig. S12**). To minimize such effects, we only compared fragments with 90% mutual overlap, and with driver

elements at least 25nt from fragment ends. Additionally, statistical power to detect allelic differences may be limited for many SNPs. We also only consider SNPs that have >20 read coverage for both fragments (reference and non-reference alleles)." (lines 333-338)

-What is the relationship between chromatin accessible QTLs (caQTLs), as measured by allelic skew in your plasmid libraries or ATAC-seq library, and allelic skew measured by RNA. Is there a trend where caQTLs tend to show a greater proportion of expression difference between alleles and if so is the direction of effect shared between the caQTL and STARR-seq signal?

We thank the reviewer for this suggestion. Unfortunately the number of SNPs we can test for allelic skew by HDRA is low (~16,000 SNPs in the genome after filtering), and only a small proportion show uncorrected nominal statistical significance (880 with raw $p<0.05$), so the overlap with any caQTL study is low. For example, we considered caQTLs identified by Banovich *et al.* (2018, PMID: 29208628), however only 123 caQTLs identified by Banovich *et al*. were tested for allelic skew in our study. We find only 3 of the 123 caQTL SNPs showing $p<0.05$ allelic skew in our study, but given the low rate of detection for SNPs with allelic skew, this difference is non-significant ($p=0.16$, Fisher's exact test). This low rate of overlap with caQTL studies was the motivation for comparing our results against CENTIPEDE "effect" SNPs (Moyerbrailean *et al*., 2016), as these effects are computationally predicted, and so the score is available for every SNP in the genome.

Reviewer #1 (Remarks to the Author):

The authors have satisfactorily addressed most of my concern and remarks.

I still have few minors remarks:

1) Typo in Supplemental Figure S8: “20+ fragments” should be “30+ fragments”

2) Supplemental Figure S1b: the identity of the three type of regions should be indicated in the figure or the legend

3) The provided Supplementary Dataset files are not labelled neither indicated in the main text

Reviewer #2 (Remarks to the Author):

The authors have responded adequately to most of my original comments. I have only one remaining comment:

- The authors claim that the fact that the i7 primer is upstream of the cloned fragments prevents PCR amplification of transcripts that would initiate from the inserted promoter fragment itself. This would be true if the inserted promoter fragments could only initiate transcription from inside their own sequence. However there is still a small possibility that a cloned promoter sequence whose transcription initiation is broad/dispersed (which is most housekeeping and some developmental promoters) could initiate from a broad region of >100bp. The key promoter elements of housekeeping promoters may be downstream (e.g. +1 nucleosome positioning signal) and since the i7 primer + 4bp + Tn5 primer are less than 60 bp total, some initiation may theoretically reach upstream of them. It is better to check using existing CAGE data.

Reviewer #3 (Remarks to the Author):

I would like to thank the authors for their detailed and thoughtful reply. My initial review contained two points of criticism. (i) Concern regarding a lack of validation for driver elements and (ii) the name and description of the experimental system used. Below are my brief thoughts to both points after taking into consideration the revised manuscript and the authors response to the reviewers.

Driver elements: The use of three additional datasets to assess the validity of the driver elements identified using HRDA significantly strengthened their conclusions. It would be helpful to include specifics regarding the overlap with Sharpr-MPRA results. (lines 272-283). For example, how many elements overlapped between the two libraries and how well did segments called as drivers replicate between studies. Also, for figure S11a and S11b it would be helpful to describe what +1,+2 and +3 is (are they windows, bp etc?).

The overlap with the Kheradpour dataset is the weakest of the three new comparisons. I have concerns the logic here is circular. My understanding of the Kheradpour dataset is the regions selected for testing were chosen because they included likely driver elements and furthermore the scrambled regions were the predicted driver motifs. Identifying elements from HDRA that overlap the Kheradpour dataset would significantly overestimate replication given the selection criteria for Kheradpour is biased. I think any overlap here is misleading and should probably be removed in its entirety.

Assay description and name: In their response the authors describe HDRA as an MPRA assay and not part of the STARR family of assays. I think this characterization is wholly incorrect. The distinguishing mark of a STARR assay is the placement of the sequence element to be tested into the 3' UTR. As the other reviewers have pointed out the use of STARR-seq to test subsets of the genome have been well documented and published. However, I disagree with the other reviewers that this perceived lack of novelty minimizes the manuscript in any way. The new framework this study provides, coupling an ATAC-STARR-seq assay (which in itself is novel) with SHARPR2 is a powerful approach and could become a standard for how the assay is used and analyzed. For this reason, I am not concerned with the novelty here. However, I disagree with the authors' reasoning for naming the assay HDRA (GPRA is another great example of unnecessary fragmentation of terminology). I find the content of the manuscript much more balanced than their reply and take issue with only the discussion paragraph starting at line 382. The authors state:

"the HDRA approach has many benefits compared to STARR-seq. HDRA preferentially tests the activity of fragments derived from open chromatin, and the HDRA library we developed achieves 130-220X more highly covered regulatory elements than random genome fragmentation."

This miss-characterizes STARR-seq as only a whole genome approach and ignores colleagues who have published other variants of STARR-seq using captured fragments. I would strongly recommend changing the first two sentences and the last sentence of this paragraph.

Response to Reviewers

We wish to thank the reviewers for their positive comments on our revised manuscript. To summarize our response to the comments:

1. Reviewer #1 was happy with our revision, and pointed out a few typos and labelling issues, which we have now addressed.
2. Reviewer #2 raised the possibility that fragments called as active in our assay have broad/dispersed upstream promoter activity, instead of enhancer activity. We have now addressed this issue using CAGE-seq data, and find that this issue affects at most 0.04% of fragments. We now discuss this issue in our Supplemental Information section that discusses future improvements on STARR-seq-based assays.
3. Reviewer #3 suggested including more information on the comparison between driver elements identified in our study and Sharpr-MPRA, and removing the comparison with the Kheradpour *et al.* study, both of which we have carried out. Based on their recommendation, we also now refer to the experimental method as ATAC-STARR-seq, and the computational method as SHARPR-RE (for random endpoints), and we only use HiDRA to refer to the combined experimental and computational method (SHARPR-RE-ATAC-STARR-seq). We have changed the acronym to be “High-resolution Dissection of Regulatory Activity” (from “High-definition Reporter Assay”), thus clarifying that the acronym refers to the combined method, not to the assay, which we call ATAC-STARR-seq. We have also re-drawn Figure 1a to clarify the unique aspects of the methodology.

We want to thank the reviewers again for their helpful comments and suggestions on our revised manuscript as well as on our original manuscript. As with our previous Response to Reviewers letter:

- We list reviewer comments in black font
- We list our response to comments in blue font
- We quote text from our original submission in green font
- We quote newly added or edited text in red font

Reviewers' comments:

Reviewer #1 (Remarks to the Author):

The authors have satisfactorily addressed most of my concern and remarks.

I still have few minors remarks:

1) Typo in Supplemental Figure S8: “20+ fragments” should be “30+ fragments”
2) Supplemental Figure S1b: the identity of the three type of regions should be indicated in the figure or the legend
3) The provided Supplementary Dataset files are not labelled neither indicated in the main text

We thank the reviewer for pointing out these oversights. We have fixed these in the revised version of the manuscript. Regarding the Supplementary Dataset files, we have embedded these in the main text and also listed these in the URLs and Data Availability section.

Reviewer #2 (Remarks to the Author):

The authors have responded adequately to most of my original comments. I have only one remaining comment:

- The authors claim that the fact that the i7 primer is upstream of the cloned fragments prevents PCR amplification of transcripts that would initiate from the inserted promoter fragment itself. This would be true if the inserted promoter fragments could only initiate transcription from inside their own sequence. However there is still a small possibility that a cloned promoter sequence whose transcription initiation is broad/dispersed (which is most housekeeping and some developmental promoters) could initiate from a broad region of >100bp. The key promoter elements of housekeeping promoters may be downstream (e.g. +1 nucleosome positioning signal) and since the i7 primer + 4bp + Tn5 primer are less than 60 bp total, some initiation may theoretically reach upstream of them. It is better to check using existing CAGE data.

We thank the reviewer for raising the possibility that inserted fragments may be initiating transcription outside of its own sequence. As suggested by the reviewer, we used existing CAGE-seq data to explore the prevalence of this phenomenon in our experiment. We downloaded a list of CAGE-seq peaks from the FANTOM consortium (peaks from any human tissue) and identified cases where a CAGE-seq peak is in the proximal 250nt upstream or downstream from a ChromHMM-defined promoter element ("TssA" chromatin state in any tissue. Of these CAGE-seq peaks, we excluded peaks that are inside another different Tss element (these peaks are likely due to initiation within the other Tss element instead of broad/dispersed initiation), and also filtered to select only CAGE-seq peaks nearby TssA elements with significant activity in our HiDRA experiments. In total, we found 26 active HiDRA regions (out of ~66,000 active regions, or approximately 0.04%) that are consistent with this scenario, suggesting this is not a major confounding factor in our experiment.

However, we believe the scenario raised by the reviewer is intriguing and could possibly occur more often in other STARR-seq assays (i.e. not ATAC-STARR, but other variations that use different plasmid designs or test fragments preferentially derived from other classes of genomic elements). For other groups that might use our experimental protocol to guide development of subsequent high-throughput enhancer assays based on the STARR-seq backbone, we have added a brief discussion of this scenario in the "**Notes for Methods**" section of the Supplemental Information. We have pasted this below:

"11) The experimental approach we used to sequence the RNA output could conceivably also include false positive results from promoters that have broad, dispersed initiation (i.e. initiation on the STARR-seq plasmid occurs immediately upstream of the inserted fragment, rather than the minimal promoter). In our dataset, promoters that appear to have the ability to promote transcription initiation in a broad upstream region (based on CAGE-seq data from the FANTOM consortium) do not have substantial activity (26 possible cases with FDR<0.05 out of ~66,000

active regions), however this possibility should be considered for future studies using this experimental approach." (Supplemental Information, Notes for Methods section)

Reviewer #3 (Remarks to the Author):

I would like to thank the authors for their detailed and thoughtful reply. My initial review contained two points of criticism. (i) Concern regarding a lack of validation for driver elements and (ii) the name and description of the experimental system used. Below are my brief thoughts to both points after taking into consideration the revised manuscript and the authors response to the reviewers.

Driver elements: The use of three additional datasets to assess the validity of the driver elements identified using HRDA significantly strengthened their conclusions. It would be helpful to include specifics regarding the overlap with Sharpr-MPRA results. (lines 272-283). For example, how many elements overlapped between the two libraries and how well did segments called as drivers replicate between studies. Also, for figure S11a and S11b it would be helpful to describe what +1,+2 and +3 is (are they windows, bp etc?).

We thank the reviewer for their positive comments on the additional validation datasets for driver elements. We apologize for the lack of clarity on the points raised regarding the comparison to the Sharpr-MPRA dataset. In our revised manuscript, we include information on the number of driver elements identified in our study that was tested by the Sharpr-MPRA paper (pasted below).

> "We found that HiDRA driver elements are much more likely to show Sharpr-MPRA activity than these shifted segments. Sharpr-MPRA scores peaked for HiDRA driver elements, and were lower in flanking regions (256 regions tested in both HepG2 and K562, Supplemental Fig. S11b)." (lines 283-285)

The original Sharpr algorithm by Ernst *et al.* does not include the functionality to call discrete driver elements, instead only providing a continuous, quantitative activity score. This is a feature that is only present in the new SHARPR-RE algorithm, which includes the ability to identify driver elements at various FDR cut-offs. Thus, we have only compared these elements in their activity scores. We clarify this in the text, which is copied below:

> "As the original SHARPR algorithm does not include the functionality to call discrete driver elements, we compared Sharpr-MPRA activity scores within driver elements identified in this study compared to equally-sized segments shifted upstream and downstream." (lines 280-283)

Additionally, in Figures S11a and S11b, we now describe the window shifting approach in greater detail:

> "+/- 1,2,3 values represent control windows of equal size to the driver element shifted upstream (-values) and downstream (+ values) by the length of the driver element." (Legend for Figure S11)

The overlap with the Kheradpour dataset is the weakest of the three new comparisons. I have concerns the logic here is circular. My understanding of the Kheradpour dataset is the regions selected for testing were chosen because they included likely driver elements and furthermore the scrambled regions were the predicted

driver motifs. Identifying elements from HiDRA that overlap the Kheradpour dataset would significantly overestimate replication given the selection criteria for Kheradpour is biased. I think any overlap here is misleading and should probably be removed in its entirety.

We thank the reviewer for this comment and reasoning and have removed the comparison with the Kheradpour dataset from the manuscript.

Assay description and name: In their response the authors describe HiDRA as an MPRA assay and not part of the STARR family of assays. I think this characterization is wholly incorrect. The distinguishing mark of a STARR assay is the placement of the sequence element to be tested into the 3' UTR. As the other reviewers have pointed out the use of STARR-seq to test subsets of the genome have been well documented and published. However, I disagree with the other reviewers that this perceived lack of novelty minimizes the manuscript in any way. The new framework this study provides, coupling an ATAC-STARR-seq assay (which in itself is novel) with SHARPR2 is a powerful approach and could become a standard for how the assay is used and analyzed. For this reason, I am not concerned with the novelty here. However, I disagree with the authors' reasoning for naming the assay HiDRA (GPRA is another great example of unnecessary fragmentation of terminology). I find the content of the manuscript much more balanced than their reply and take issue with only the discussion paragraph starting at line 382. The authors state:

"the HiDRA approach has many benefits compared to STARR-seq. HiDRA preferentially tests the activity of fragments derived from open chromatin, and the HiDRA library we developed achieves 130-220X more highly covered regulatory elements than random genome fragmentation."

This miss-characterizes STARR-seq as only a whole genome approach and ignores colleagues who have published other variants of STARR-seq using captured fragments. I would strongly recommend changing the first two sentences and the last sentence of this paragraph.

We thank the reviewer for this comment and for his/her positive comments on the novelty and potential future impact of our study. As recommended by the reviewer, we now describe the HiDRA assay as a combination of ATAC-STARR-seq and SHARPR-RE. As we now describe in the Discussion, we refer to this joint assay (i.e. SHARPR-RE-ATAC-STARR-seq) as HiDRA for simplicity:

We also change the acronym HiDRA to “High-resolution Dissection of Regulatory Activity” (from High-Definition Reporter Assay), thus making it clear that the acronym refers to the combined ATAC-STARR-seq + SHARPR-RE experimental+computational methodology.

> “In the experimental component of HiDRA, we capture regions of open chromatin, size selecting them, and transfect them downstream of transcription start in an episomal reporter construct, thus driving their own transcription and serving as their own barcodes (ATAC-STARR-seq). By concentrating the signal on open-chromatin regions, HiDRA enables high-resolution inference of driver nucleotides within these regions using a machine-learning model (SHARPR-RE), by exploiting subtle differences in the reporter activity driven by partially-overlapping tested fragments. We refer to the combined SHARPR-RE-ATAC-STARR-seq method as “High-resolution Dissection of Regulatory Activity”, or HiDRA.” (lines 365-371)

For the section quoted by the reviewer, we have edited the text to clarify that the experimental steps in HiDRA (i.e. ATAC-STARR) has major advantages over the regular genome-wide STARR-seq approach without fragment selection:

> "The ATAC-STARR-seq step in HiDRA has many benefits compared to regular STARR-seq without fragment selection. ATAC-STARR-seq preferentially tests the activity of fragments derived from open chromatin, and the HiDRA library we developed achieves 130-220X more highly covered regulatory elements than random genome fragmentation" (lines 395-398)

Finally, we also changed the abstract and introduction sections to emphasize that HiDRA is a combination of ATAC-STARR-seq and SHARPR-RE inference of driver nucleotides:

Abstract:

> "Experimentally, we test ~7 million accessible DNA fragments in a single experiment, by coupling accessible chromatin extraction with self-transcribing episomal reporters (ATAC-STARR-seq). By design, fragments are highly overlapping in densely-sampled accessible regions, enabling us to pinpoint driver regulatory nucleotides by exploiting differences in activity between partially-overlapping fragments using a machine learning model (SHARPR-RE)." (Abstract)

Introduction:

> "Briefly, we first extract accessible DNA regions using ATAC-seq, size-select for constructs 150-500nt long, and incorporate them in self-reporting episomal constructs (ATAC-STARR-seq), by insertion in the 3' untranslated region (3' UTR) of reporter genes, thus enabling them to drive their own transcription and serve as their own barcodes, providing a quantitative readout of their activity. We then exploit the dense sampling of accessible regions and the partially-overlapping nature of tested fragments for high-resolution inferences using a machine-learning approach (SHARPR-RE)." (lines 35-41)

Reviewer #2 (Remarks to the Author):

The authors have adequately addressed my last outstanding concern.

Reviewer #3 (Remarks to the Author):

The authors have fully addressed all of my comments.
